# Finding continuity and discontinuity in fish schools via integrated information theory

**DOI:** 10.1371/journal.pone.0229573

**Published:** 2020-02-27

**Authors:** Takayuki Niizato, Kotaro Sakamoto, Yoh-ichi Mototake, Hisashi Murakami, Takenori Tomaru, Tomotaro Hoshika, Toshiki Fukushima

**Affiliations:** 1 Faculty of Engineering, Information and Systems, University of Tsukuba, Tsukuba, Ibaraki, Japan; 2 University of Tsukuba, Leading Graduate School Doctoral Program in Human Biology, Tsukuba, Japan; 3 The Institute of Statistical Mathematics, Tachikawa, Japan; 4 University of Tokyo, Research Center for Advanced Science and Technology, Tokyo, Japan; 5 Department of Computer Science and Engineering, Toyohashi University of Technology, Toyohashi, Japan; Arizona State University & Santa Fe Institute, UNITED STATES

## Abstract

Collective behaviours are known to be the result of diverse dynamics and are sometimes likened to living systems. Although many studies have revealed the dynamics of various collective behaviours, their main focus has been on the information processing performed by the collective, not on interactions within the collective. For example, the qualitative difference between three and four elements in a system has rarely been investigated. Tononi et al. proposed integrated information theory (IIT) to measure the degree of consciousness Φ. IIT postulates that the amount of information loss caused by the minimum information partition is equivalent to the degree of information integration in the system. This measure is not only useful for estimating the degree of consciousness but can also be applied to more general network systems. Here, we obtained two main results from the application of IIT (in particular, IIT 3.0) to the analysis of real fish schools (*Plecoglossus altivelis*). First, we observed that the discontinuity on 〈Φ(*N*)〉 distributions emerges for a school of four or more fish. This transition was not observed by measuring the mutual information or the sum of the transfer entropy. We also analysed the IIT on Boids simulations with respect to different coupling strengths; however, the results of the Boids model were found to be quite different from those of real fish. Second, we found a correlation between this discontinuity and the emergence of leadership. We discriminate leadership in this paper from its traditional meaning (e.g. defined by transfer entropy) because IIT-induced leadership refers not to group behaviour, as in other methods, but the degree of autonomy (i.e. group integrity). These results suggest that integrated information Φ can reveal the emergence of a new type of leadership which cannot be observed using other measures.

## Introduction

Collective behaviours in nature are emergent properties produced through the local interactions of self-organising individuals. Such behaviours include swarming [1–6], fish schooling [7–11], bird flocking [12–17], or high-level cognitive functions arising from ‘bottom-up’ neural networks [18–21]. These systems of many interacting elements can achieve optimal information processing capabilities when poised at the critical boundary separating chaos from order [22–24]. Several analyses have demonstrated individuals responding swiftly as a collective to changing environments [14–16] and that a group can achieve relatively good decision-making [25–27]. Conflicts among individuals do not necessarily lead to group disruption and can instead provide valuable insight into what collective responses are effective [28, 29]. The unity of collective behaviours remains an unsolved question of nature [30–32], because the interactions are hidden, whereas the resultant actions are observable.

Self-organised criticality (SOC) is a valuable metaphor for interpreting complex collective behaviours. Flexibility and robustness can be achieved collectively when a group is in the intermediate state between order and disorder [33–38]. For instance, the external perturbations of collective systems in SOC models optimise the effective correlation range of each individual to efficiently transfer information [13–16]. However, the SOC models are unreliable for small groups, because individual interactions are less likely to be homogeneous [39, 40]. In fish, for example, groups of two and three appear to exhibit differential between-individual interactions [41, 42]. Therefore, many researchers have examined information transfer (or causal relationships) among individuals in small groups [43–45], often employing local transfer entropy (TE) [46–51]. For example, the transfer of misinformation can happen in schools with five fish when the whole group changes direction [45]. Animals could potentially use active information storage to predict the timing of nontrivial information transfer [52, 53]. However, despite capturing some ‘what the system does’ aspects of collective behaviour systems (that is,‘happening’: the actual behaviour), SOC models provide little insight on ‘what the system is’ (or ‘being’: the potential dynamical complexity). A detailed discussion on the difference between ‘being’ and ‘happening’ can be found in [54] and [55].

In contrast, integrated information theory (IIT) may be a good metric for ‘what the system is’. Proposed by Tononi and Sporns [56, 57] in 2001, IIT was originally developed to quantify consciousness from brain activity [58, 59]. In principle, the core concept of IIT is to define integrated information as the degree of information loss (or increase in uncertainty [60]) caused by a given partition of the system [59]. Integrated information is designed to quantify the holistic amount of cause–effect power possessed by a system that goes beyond and above the sum of its parts. The latest version of IIT (IIT 3.0 [61]) postulates that an arbitrary subset of elements of the system is a ‘mechanism’ if its intrinsic cause–effect power, i.e. the ability to determine the future and constrain the past states of other arbitrary parts (‘purviews’), is irreducible to the separate and independent actions of parts of the mechanism over parts of the purview. This irreducibility is measured in terms of integrated information *φ*. Iterated at the system level, the bipartitions are applied to the set of all irreducible mechanisms and their purviews, which together form the systems’s ‘conceptual structure’. The minimal distance between intact conceptual structure and the conceptual structure under the partition is defined as the *integrated information* (denoted by Φ) of the system. A system is integrated, even if the least disrupting bipartition of the system, which is called the ‘minimum information partition’ (MIP) and partitions the system into two disconnected halves, would imply a loss of information in the causal power of the system (the partition is unidirectional and thus only cuts the connections from one half to the other but not the other way around). Instead of assessing whether a system is unified into a coherent whole by analysing its behaviour under regular conditions, IIT proposes that the forces integrating the behaviour of the system are better captured by observing its behaviour under perturbations. Integrated information formalises the interventionist notion of characterising the causal influences among the components of the system [62, 63]. This formalism is believed to better capture the intrinsic causal structure of the system.

Recent studies have suggested that measures based on the general ideas of IIT can capture various states of lost consciousness, such as dreamless sleep [64], general anaesthesia [65], or vegetative states [66]. Some studies have suggested that integrated information could act as an order parameter of complex systems, similar to the generalised Ising model [67], coupled oscillators [68], and coupled mapping [69]. The magnitude of IIT 2.0 integrated information and the susceptibility of IIT 3.0 integrated information *σ*(Φ) peaks at critical points.

IIT has several versions [58–61, 70–72, 73] (Computational comparison of these versions of IIT was made by Mediano [74]). For example, there is a fundamental difference between IIT 2.0 [58, 59] and 3.0 [61]: one uses Riemann geometry and the other uses Wasserstein geometry. While both IIT 2.0 and 3.0 can capture phase transitions of the system, the differences occur in the behaviour at the criticality: Φ in IIT 3.0 does not necessarily peak at the critical point (the susceptibility of Φ peaks at the critical point) whereas *φ* in IIT 2.0 works as an order parameter. Note that Ito [75, 76] has pointed out that there are some intimate relations between the second law of information thermodynamics and IIT in terms of a projection onto a local reversible manifold. These structural resemblances suggest the possibility of unifying the concept of non-equilibrium thermodynamics and IIT 2.0. The extension of this idea to IIT 3.0 is still unknown.

Integrated information theory 3.0 is also resonant with complex systems because it posits that the whole cannot be reduced into its parts; the components produce synergetic information that would be lost upon separation [77, 78]. Complex biological systems are also irreducible owing to their intrinsic causal structures [79]. When applying IIT to these systems, Φ can be a measure of an autonomous system’s wholeness [77], capturing intrinsic causal structures [80] while also acting as its order parameter. As an initial step to modelling collective animal behaviour, some researchers have used IIT to interpret classifications of cellular automatons [54], animats [81], neuron-astrocyte ensembles [82], and Boolean networks [83]. For instance, the averaged max Φ values of major complexes for five to six automata cells correlated well with their complexity (e.g. class III and IV rules), despite the small cell-set number [54]. The behaviours of five and six automata cells are hard to discriminate on the basis of the behaviours of their constituent cells and, in general, the behaviours of small numbers of cellular automata are very similar with each other in terms of an external observer. They also showed that all rules of class IV have all orders of concepts (i.e. irreducible subsets in the system), unlike other classes. In addition to this study, many recent studies have found the connexion among IIT, group interaction, autonomy, criticality, edge of chaos, and a dynamical system (see [69, 84–87]). Linking IIT to collective behaviour seems very natural in this respect. Assuming that biological systems such as schools of fish evolved to reside in a critical state because of its advantages such as fast information transfer, the concept of network controllability using values of Φ may also provide meaningful insight into collective animal behaviour.

The example of cellular automata illuminates the meaning of intrinsic properties in IIT. IIT reveals the differences among systems arising from different intrinsic causal structures, rather than considering differences based on external behaviour. That is why we stated that previous approaches (e.g. TE) capture not ‘what the system is’ but ‘what the system does’. This enables us to investigate the difference in collective behaviour in terms of the intrinsic causal structure. In this paper, we investigate the following: whether the number of agents in a system affects its intrinsic properties. In other words, if the group size changes, we would like to know what remains the same (continuous) and what changes (discontinuous) in the group. In addition, we consider whether there are any new factors introduced that were not present before. These kinds of questions are rarely asked in animal collective behaviour, but one study suggests that schools of three fish and schools of two fish have different kinds of interactions [41, 42]. Another suggests that the search strategies of fish in groups of different sizes are essentially different when they are in an unfamiliar environment [11]. However, all these studies constrain the number of individuals in the group to three or less and their methods are difficult to generalise to larger groups. Furthermore, these methods never indicate any differences in terms of a group’s intrinsic causal structure.

In this paper, we apply IIT (in particular, IIT 3.0 using PyPhi [61, 72]) to schools of two to five fish (*Plecoglossus altivelis*) and show the intrinsic differences between these groups. We found that there is a kind of continuity and discontinuity with respect to school size. The main finding is that there is a discontinuity between three- and four-fish schools, which is a difference that has not received much attention previously. Interestingly, the difference between these two systems corresponds to the existence of leadership (more precisely, reducing the visual field for a fish’s recognition introduces the existence of the leadership). Furthermore, our results are never replicated by this discontinuity between three and four fish in a Boids-type model for the same conditions.

## Results

### Application of IIT 3.0 to fish school analysis

**Fig 1:**
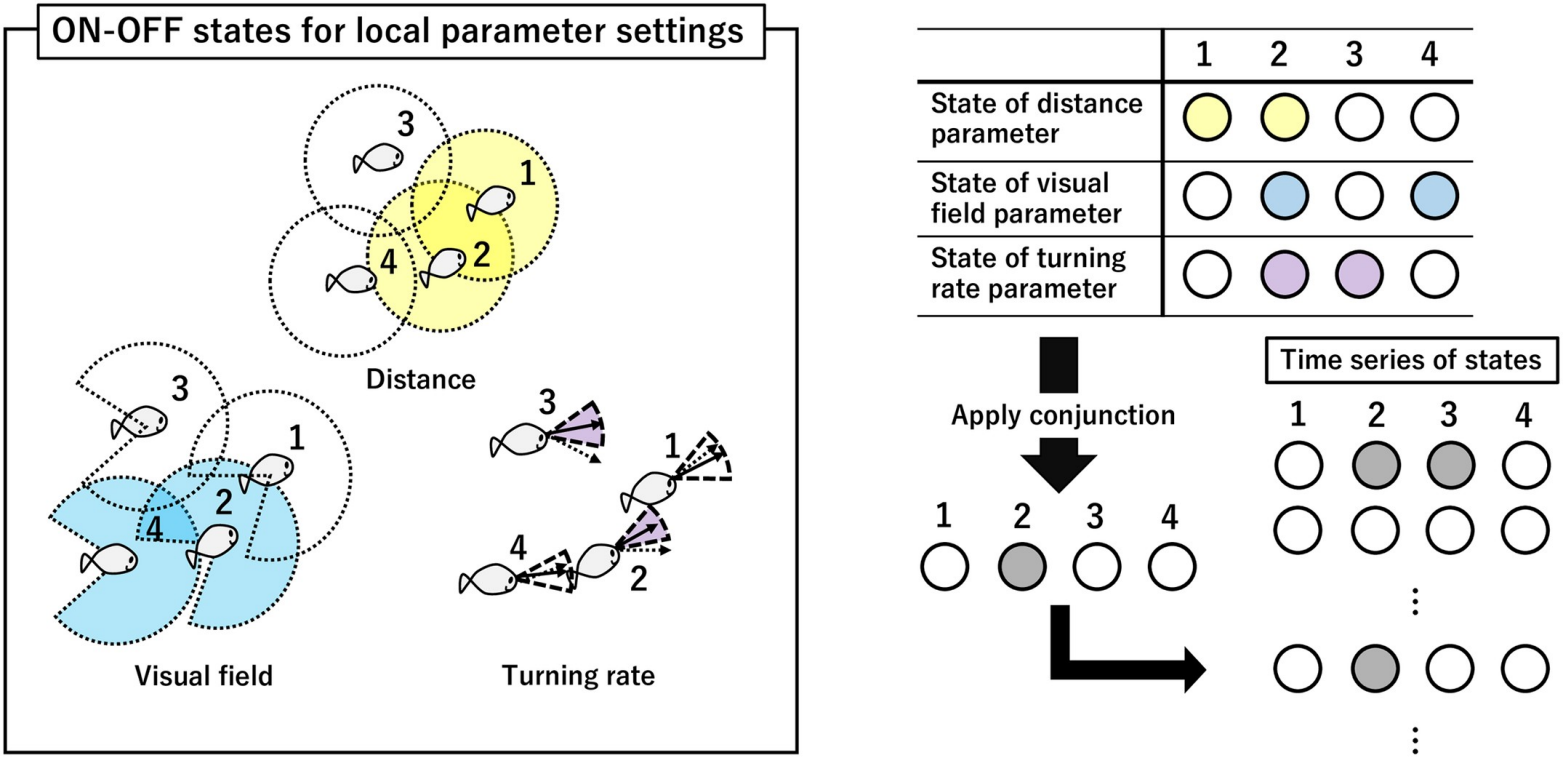
ON and OFF states for local parameter settings. Three parameters determine a school’s state (yellow: effective distance, blue: effective visual field, and purple: turning rate). Colour indicates the ON state.

We tracked the trajectories of *ayu* fish schools of *N* = 2, *N* = 4, and *N* = 5 with three samples each, and *N* = 3 with four samples (10–15 minutes recording length; see Materials and methods: Experimental settings for details). We then used these trajectories for analysis. To apply IIT 3.0, we define the ON and OFF states of an individual in a fish school. In this paper, an ON state means some interaction will occur in a given context. Note that in the Boids model [8, 88, 89], at least three kinds of interactions are needed: (i) interaction radius (distance), (ii) visual field, and (iii) turning rate (see Fig 1). (i) For interaction radius, if two individuals are within a certain radius, the states of both individuals are ON (some information transfer will occur between them). This is a symmetric distance interaction. (ii) A visual field interaction means the individual is in the ON state when some other agents are within its visual field. This allows us to consider asymmetrical relations (in contrast to the symmetric distance condition). (iii) The turning rate interaction is one in which a direction change above a certain angle puts the individual in the ON state. This ON state transfers information to other agents in the next time step, so the interaction between individuals is a delayed one. The rate of direction change is a very important measure for collective behaviour, empirically and theoretically [8, 88, 89].

The first two parameters (’effect distance’ and ‘effective visual field’ interactions) represent the interaction radius with the agent’s blind spot (in later sections, we often refer to the environment outside of the visual field as the blind spot). The third parameter ‘turning rate’ represents the result of that interaction. Therefore, the final state is the conjunction of the states of all three parameters: distance, visual field, and turning rate (as detailed below). This representation reflects the commonly used interactions in a flocking model.

Note that the central interest of this paper is not the fixed individual’s perceptual abilities (e.g. the fixed visual field) but the individual’s effective perceptions under the dynamical interactions [13]. To state this more precisely, the interaction radius and the visual field should be interpreted as the ‘effective’ interaction radius and the ‘effective’ visual field.

To determine each fish state, the continuous trajectories of fish schools are quantised into ‘Ising-like’ binary time series. Biologically, such discretisation makes sense in terms of individual’s contact, corresponding to the ON/OFF state, which is a good measure for a fish’s communication in the school. Murakami et al. suggest that each fish’s Levy walk behaviour in a *Plecoglossus altivelis* school enhances the efficient group communication through mutual contacts [10]. Therefore, it is reasonable to assume mutual contacts in the group reflect biological meaning to the group.

In this paper, we assume a fish always evaluates these three parameters simultaneously. Hence, we take their conjunction (i.e. AND:{0, 1}^3^ → {0, 1}. For the triple (1, 1, 1), AND(1, 1, 1) is 1; otherwise, it is 0) to produce the overall state for a fish. For instance, IF (Distance = ON, Visual field = ON, Turning rate = OFF), THEN state = OFF. Note that the use of the AND operations reflects a Boid-like interaction (interaction radius with blind spot and direction changing). If any AND is replaced with an OR, the Boids-like interaction settings become meaningless. Applying the same process to each fish at time *t*, we obtain the time series of the states of the *n* fish. Then, we can compute Φ and other values (the sum of *φ*) from the obtained time series. One time step, in this paper, is defined as 0.05 to 0.10 s. This value roughly corresponds to a fish’s reaction timescale [45].

To compute Φ, we also define the network structure in the school. In this paper, we postulate a completely connected network including self-loops (i.e. including self history). This assumption comes from the experimental fact that each fish has some contact with (or falls within the visual field of) all individuals in the group during a long series of recorded events (10–15 min). Therefore, it is natural to assume that some interactions have happened among all members. (In Table 3, we give the minimal distance that occurs during the events. The data show that all fish have a contact that is within 5 mm).

Before elaborating on our analysis, it is necessary to understand what the states ON and OFF mean for the fish. Biological information systems, such as the brain, have an explicit ON state, that is, when neurons are firing. In contrast, the ON state for each fish occurs when it interacts with its environment. Because there are various kinds of information to take into account, there is no explicit ON state for a fish school. Therefore, we computed all Φ values for all combinations of our parameter settings. Note that these combinations also contain only one or two of the three parameters. For instance, 2*π* rad given as the visual field and 0 rad/s given as the turning rate change any individual state to ON. The distance parameter alone determines individual ON/OFF states in this condition (mathematically, this is AND(*x*, 1, 1) = *x*, where *x* is ON or OFF according to the distance parameter).

### Φ values for real fish school data

#### MI, TE, and IIT 3.0

Mutual information (MI) and TE are often used to analyse dynamical systems. In this section, we explain how the Φ values of IIT 3.0 make it a unique measure compared with other well-known measures.

We compare three measures, that is, MI, TE, and Φ (IIT 3.0) under the same conditions. This comparison is possible because they share the same time series of an individual’s states (Fig 1). From these generated state sequences, we can compute the unique MI, TE, and Φ. In this paper, all analysis is restricted to the pair of parameters distance and visual field because the peak values always exist under a fixed turning rate condition of 0 rad/s. The importance of turning rate can be found over a longer time scale (see [90]). Instead, to verify that the following results are robust, we listed other time scale settings and Φ values as the main complex condition, adding to the results of other parameter settings, in the supporting figures.

Let the present and past states of the system at time *t* be given by *X*(*t*) = {*x*_1_(*t*), *x*_2_(*t*), …, *x*_*n*_(*t*)} and *X*(*t* − 1) = {*x*_1_(*t* − 1), *x*_2_(*t* − 1), …, *x*_*n*_(*t* − 1)}. Elements *x*_*i*_(*t*) and *x*_*i*_(*t* − 1) respectively represent fish *i*’s present and past states (and are 0 or 1), and *n* is the size of the fish school. Then, we have probability distribution *p*(*x*_*i*_) = *Pr*{*X* = *x*_*i*_} and Shannon entropy $H\left(X\right)=-\sum_{i=1}^{N}p\left(x_{i}\right)\text{log}(p\left(x_{i}\right))$. MI is expressed as follows:

$$\begin{array}{c} MI=I(X(t);X(t-1))=H(X(t))-H(X(t)|X(t-1)) \end{array}$$ (1)

This equation measures the shared information with the past (the states of the previous iteration, in this paper, Δ = 0.05 s) and the present collective states. The collective state is the total ON and OFF sequence for the given parameter settings. For example, in the two fish schools, the collective state consists of four states, that is, *X* = {0, 0}, {0, 1}, {1, 0}, or {1, 1}.

In contrast, the TE measures the information transfer between two agents under the condition of knowing the other agent’s history (one step before the agent’s state is Δ = 0.05 s). We define the TE as the sum of all pairwise transfer entropies.

$$\begin{array}{c} TE=\sum_{\begin{array}{c} (i,j)\in N\times N\\ i\neq j \end{array}}T_{x_{i}\left(t\right)\to x_{j}\left(t\right)}=\sum_{\begin{array}{c} (i,j)\in N\times N\\ i\neq j \end{array}}\left(H\left(x_{j}\left(t\right)|x_{j}\left(t-1\right)\right)-H\left(x_{j}\left(t\right)|x_{i}\left(t-1\right),x_{j}\left(t-1\right)\right)\right) \end{array}$$ (2)

The computation of IIT 3.0 is more complicated. For a given system in a particular state, all possible mechanisms (or subsets of system nodes in a state) that irreducibly constrain the past and future states of the system are identified. For each mechanism, all possible purviews or subsets of nodes that the mechanisms constrain are considered. For a given mechanism–purview combination, its cause–effect repertoire (CER; a probability distribution specifying how the mechanism causally constrains the past and future states of the purview). To find the irreducibility of the CER, the connexions between all permissible bipartitions of elements in the purview and the mechanism are cut; the bipartition producing the least difference is called the MIP. The irreducibility, or integrated information, *φ*, is quantified by the earth mover’s distance between the CER of the uncut mechanism and the CER of the mechanism partitioned by the MIP. A mechanism, together with the purview over which its CER is maximally irreducible and the associated *φ* value, specifies a concept, which expresses the causal role played by the mechanism within the system. The set of all concepts is called the cause–effect structure of the system. Once all irreducible mechanisms of a candidate system have been found, a similar set of operations is performed at ‘system level’ to understand at what level the set of mechanisms specified by the system are reducible to the mechanisms specified by its parts. The irreducibility of the candidate system is quantified by its conceptual integrated information Φ. This process is repeated for all candidate systems, and the candidate system that is maximally irreducible among all candidate systems is called the major complex.

**Fig 2:**
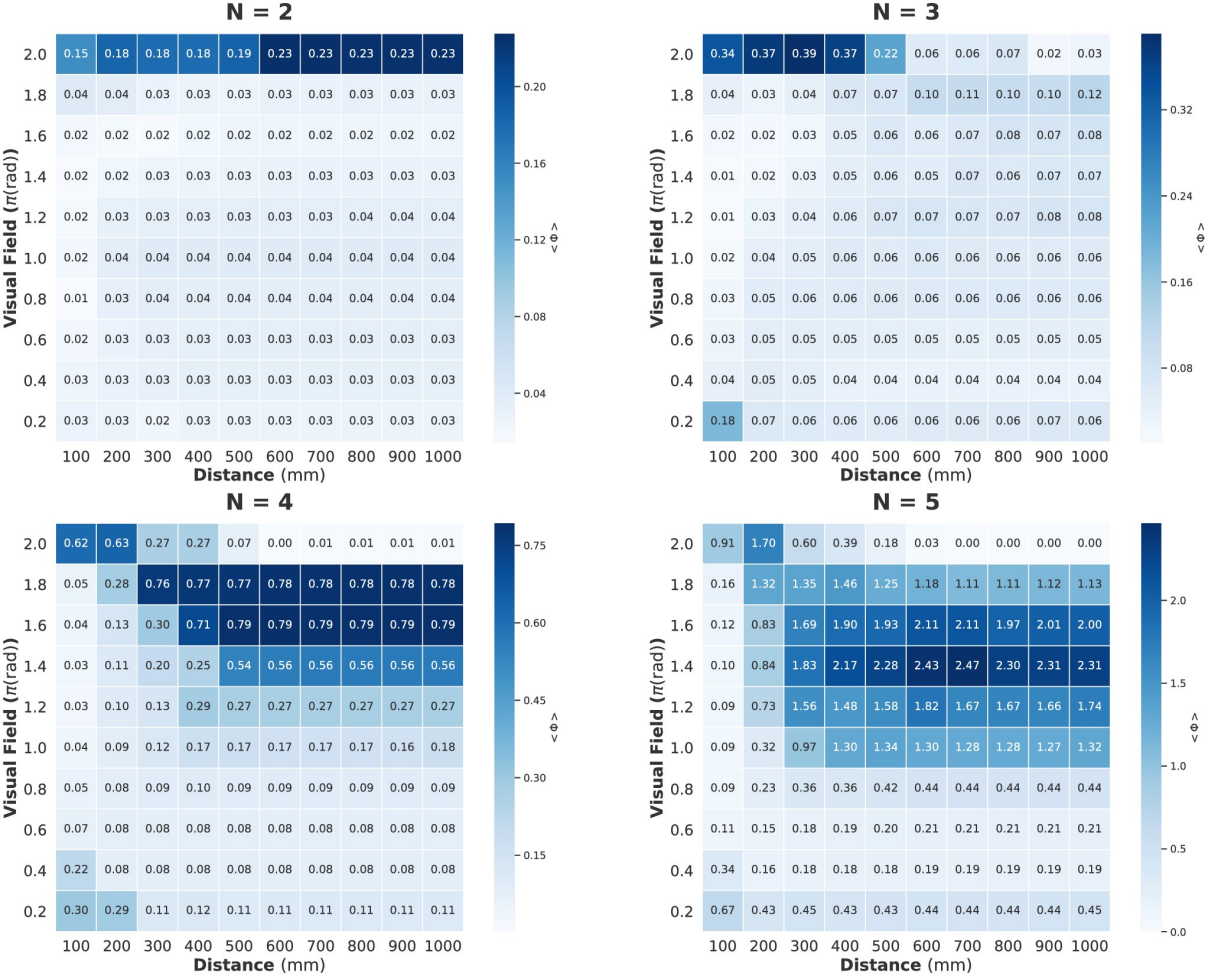
Heat maps showing the 〈Φ(*N*)〉 values for distance (horizontal axis) and visual field (vertical axis) parameter values. Each cell value corresponds to the average 〈Φ(*N*)〉 on all samples (three samples for *N* = 2, 4, 5 and four samples for *N* = 4). The value of 〈Φ(*N*)〉 for two- and three-fish schools depends only on distance, whereas it depends on both parameters in four- and five-fish schools. The time step is Δ*t* = 0.05 s.

We present a summary of IIT 3.0 in the Methods section. For readers unfamiliar with IIT 3.0, it is sufficient to interpret Φ as representing the degree of group integration. However, we have two remarks. (i) Each collective state has its own Φ. Hence, *n*-fish schools have a 2^*n*^ collective state. For example, in the case of a two-fish school in which all collective states are (0, 0), (1, 0), (0, 1), and (1, 1), we have four Φs, that is, Φ_00_, Φ_10_, Φ_01_, and Φ_11_. (ii) The average 〈Φ(*N*)〉 is that of all Φ values on all collective states, given by certain parameter settings. The values of the heat map in Fig 2 refer to the average of the mean 〈Φ〉 from all data samples. In this paper, although we computed *n*-size subsystem for *n*-fish schools, the distribution of Φ of the main complex also yields the same results S4 Fig.

**Fig 3:**
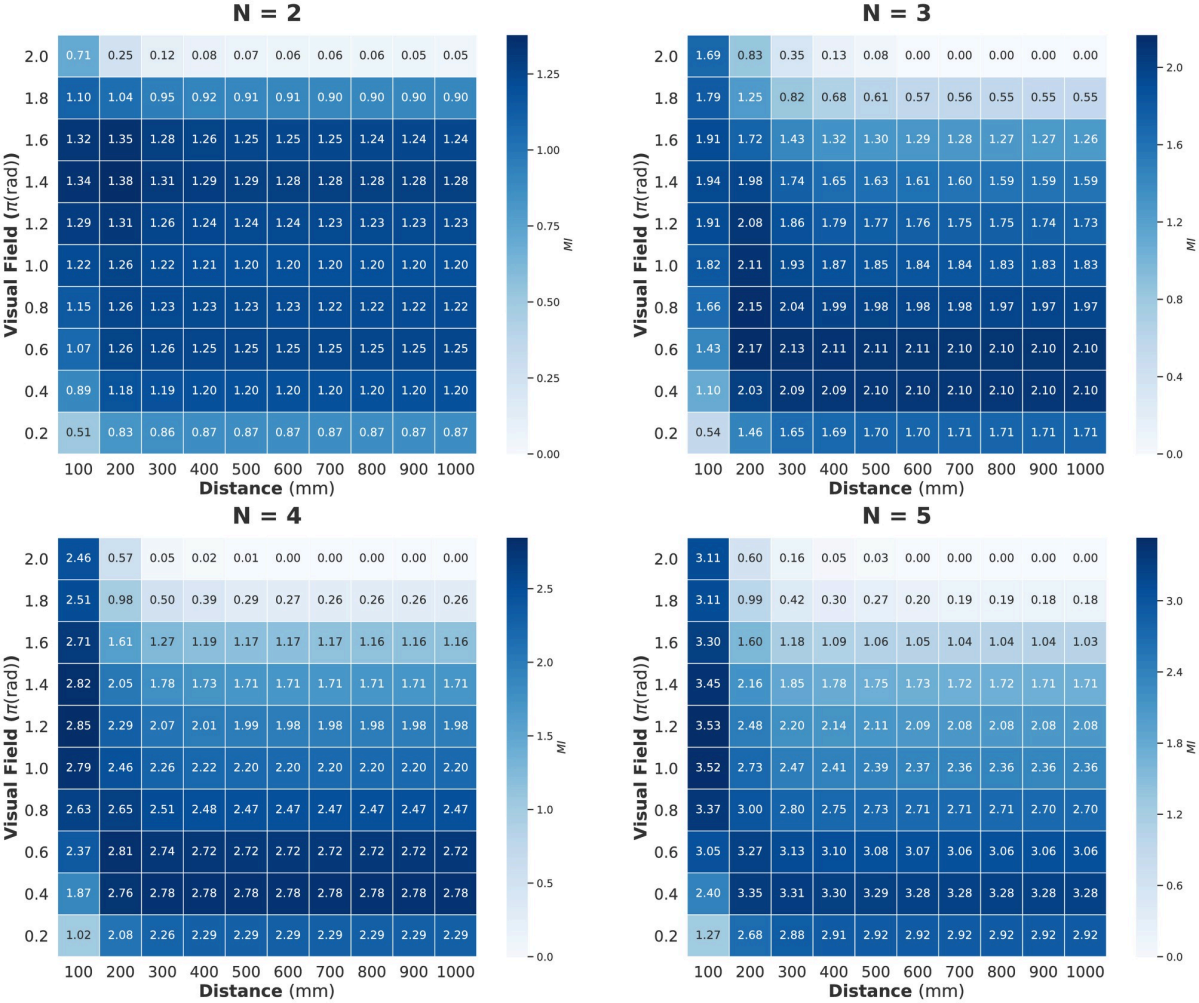
Heat map showing mutual information (MI) for distance (horizontal axis) and visual field (vertical axis) parameter values. Each cell value corresponds to the average MI on all samples. The time step is Δ*t* = 0.05 s.

**Fig 4:**
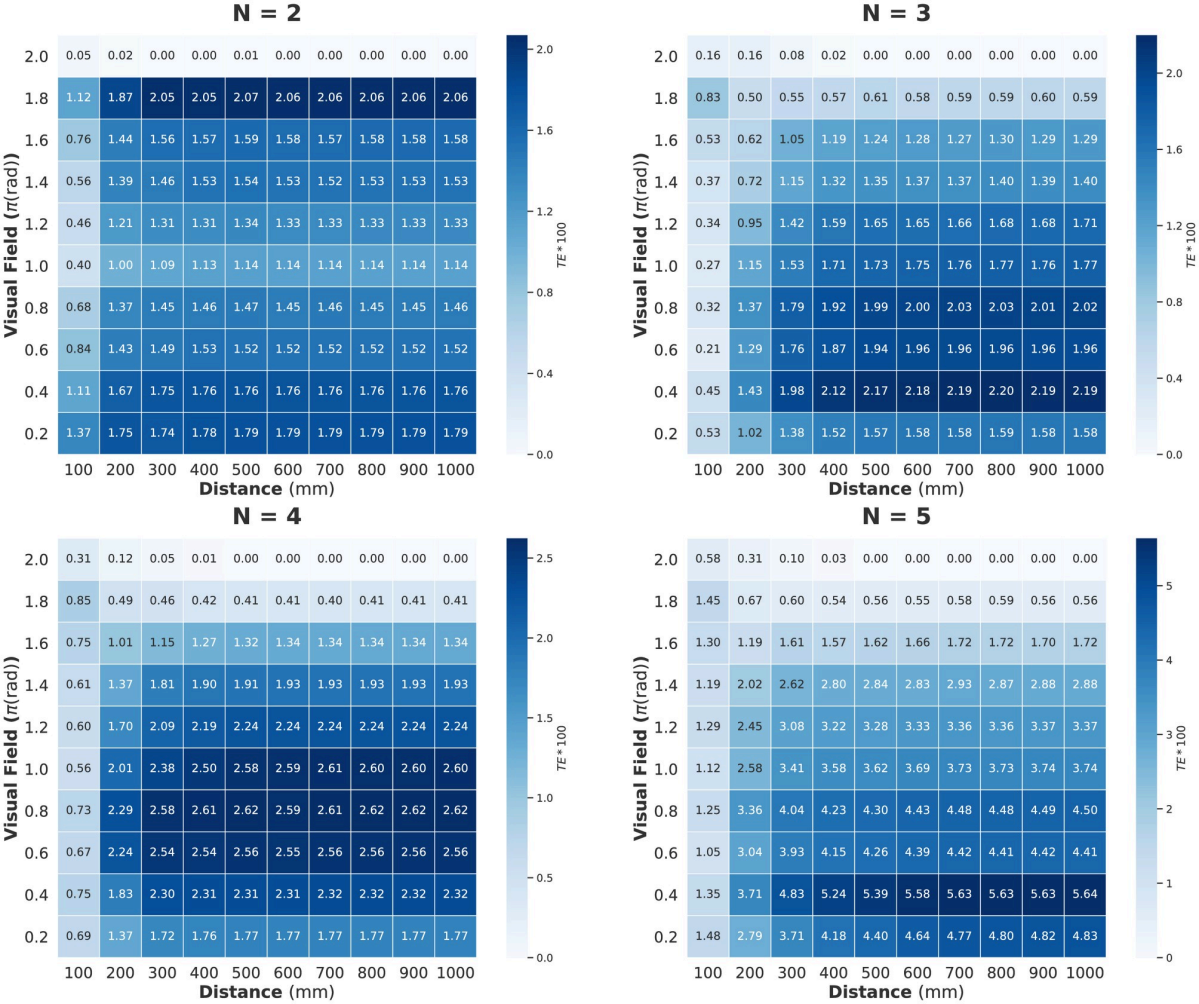
Heat maps showing the sum of transfer entropy (TE) for distance (horizontal axis) and visual field (vertical axis) parameter values. Each cell value corresponds to the average *TE* on all samples. Note that each *TE* value is multiplied by 100 for contrast). The distribution of *TE* resembles that of *MI*. In contrast, *TE* does not become larger than the observed *MI*. This is because each pairwise information transfer in large groups is less than those of small groups. The time step is Δ*t* = 0.05 s.

Figs 2, 3 and 4 show the computed values for two parameter settings (distance and visual field: turning rate is fixed at 0 rad/s); Fig 2 shows 〈Φ(*N*)〉, Fig 3 shows MI, and Fig 4 shows the sum of the TE. We can confirm the sharp contrast between 〈Φ(*N*)〉 and the other two measures. The peaks of MI and TE are mainly concentrated in the low visual field area; whereas the peaks of 〈Φ(*N*)〉 seem to have completely opposite trends (for the distribution including the turning rate, see S1 Fig. In this study, we mainly discuss the relation between these two parameter settings and 〈Φ(*N*)〉 because the 〈Φ(*N*)〉 values are not very high when the turning rate is non-zero). Interestingly, the distribution of 〈Σ*φ*〉 resembles that of MI and TE but not Φ (S2 Fig). The distribution of 〈Φ(*N*)〉 remains the same when Δ*t* is 0.1 s (S3 Fig).

A further outstanding fact is the existence of a discontinuity between three- and four-fish schools in the 〈Φ〉 distributions. Note that the term ‘discontinuity’ (resp. ‘continuity’) that we use here is the inter-relation between two distributions for the group size and is not the intra-relation for the parameter settings in the same heat map. We can confirm that a qualitative shift happens between three- and four-fish schools (see Table 1). This trend was not observed in MI and TE (and 〈Σ*φ*〉). All these facts imply that the 〈Φ(*N*)〉 in IIT 3.0 captures something unique that is not observed when the traditional MI and TE metrics are used.

**Table 1:** Statistical test for the discontinuity on Fig 2. 〈Φ(*N*)〉_*x*_: means 〈Φ(*N*)〉 from D = 100 mm to D = 1000 mm at fixed VF = *x* rad, where D is distance parameter and VF is visual field parameter. The p-values for 〈Φ(*N*)〉_1.8*π*_ and 〈Φ(*N*)〉_2.0*π*_ were computed using the Weltch t-test. Values in bold correspond to the large mean Φ values. The opposite trend of 〈Φ(*N*)〉 emerge between three- and four-fish school.

	〈Φ(*N*)〉_1.8*π*_	〈Φ(*N*)〉_2.0*π*_	p-value
***N* = 2**	0.002	**0.176**	< 10^−10^
***N* = 3**	0.030	**0.112**	< 10^−7^
***N* = 4**	**0.517**	0.124	< 10^−8^
***N* = 5**	**1.470**	0.391	< 10^−8^

**Fig 5:**
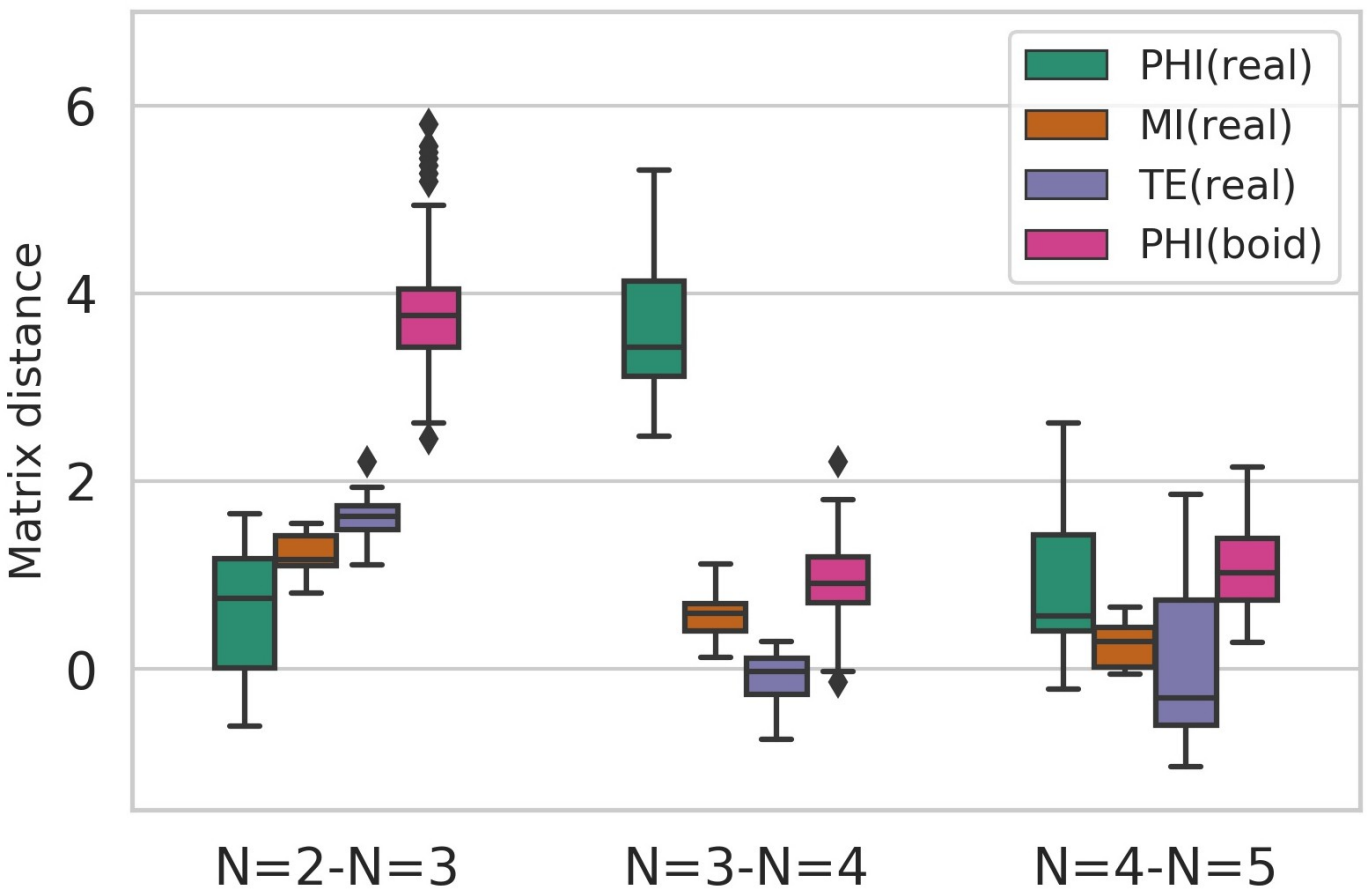
The mean matrix distance (MD) between inter-distributions subtracted by the baseline (the mean matrix distance among intra-distribution). The mean MD of PHI (real) between three- and four-fish schools is significantly larger than the other two measures and PHI (boid). PHI (boid) data is the same as the data shown in Fig 9.

Fig 5 provides a whole picture on the difference between the inter-relation of each heat map (PHI (real), MI, TE, PHI (boid)): The data of PHI (boid) are in Fig 9). The vertical axis is the mean matrix distance (MD) between the inter-distributions of different group sizes. Note that in this study, we subtracted the MD from the baseline MD. This baseline MD corresponds to the average of all intra-distribution (N = 2, N = 3, N = 4, and N = 5) matrix distances for each measure.

The mean MD of PHI (real) between N = 3 and N = 4 distribution is much higher than the other two measures (Weltch t-test: MI(*p* < 10^−10^), TE(*p* < 10^−13^) and PHI(boid)(*p* < 10^−35^). Furthermore, the mean MD of PHI (real) between N = 3 and N = 4 distributions is also higher than that between N = 2 and N = 3 (Table 1). In contrast, the other three show opposite trends, as illustrated in Fig 5. They have relatively large values for N = 2 and N = 3 than those of N = 3 and N = 4. Therefore, the graph suggests a discontinuity between three- and four-school distributions that were only confirmed in the 〈Φ(*N*)〉 value distributions of real fish.

#### Emergence of leadership: Discontinuity between real three- and four-fish schools

We consider Fig 2 in detail. The concept of IIT 3.0 implies that Φ represents the group integration. If the group integration assigns some merit for group behaviour, to verify the meaning of high integration, it would be helpful to understand the collective behaviour. As we have already noted, there is a discontinuity between three- and four-fish schools. The striking contrast seems to exist when high visual field area ranges from 1.6*π* to 2.0*π* rad. Here, 2.0*π* rad means there is no blind spot for each fish; whereas the values less than 2.0*π* rad mean that each fish has some blind spot. Taking into account this contrast, fish schools of less than four show a high group integration (high 〈Φ(*N*)〉) when their effective visual field is intact; in contrast, fish schools of four or more fish exhibit high group integration (high average 〈Φ(*N*)〉) when their effective visual field has a blind spot.

In this section, we explore this issue more deeply in terms of IIT. Focusing on whether the visual field parameter is 2*π* or not, we see the discontinuity on 〈Φ(*N*)〉 distribution as the ‘emergence of leadership’. In this paper, we assume that the leader of the group should satisfy the following properties: (i) the leader is the head position of the group (i.e. the positional leader) and (ii) there is asymmetrical information flow among the leader and the rest of members. These kinds of properties seem ubiquitous in animal collective behaviour [91–94]. We aim to show a single OFF state in the group that highly correlates with these two properties, that is, (i) a single OFF state individual that shows high correlation with the positional leader and (ii) an MIP cut between the OFF-state fish and the rest of the ON-state fish corresponds to asymmetrical information flow in the group.

**Fig 6:**
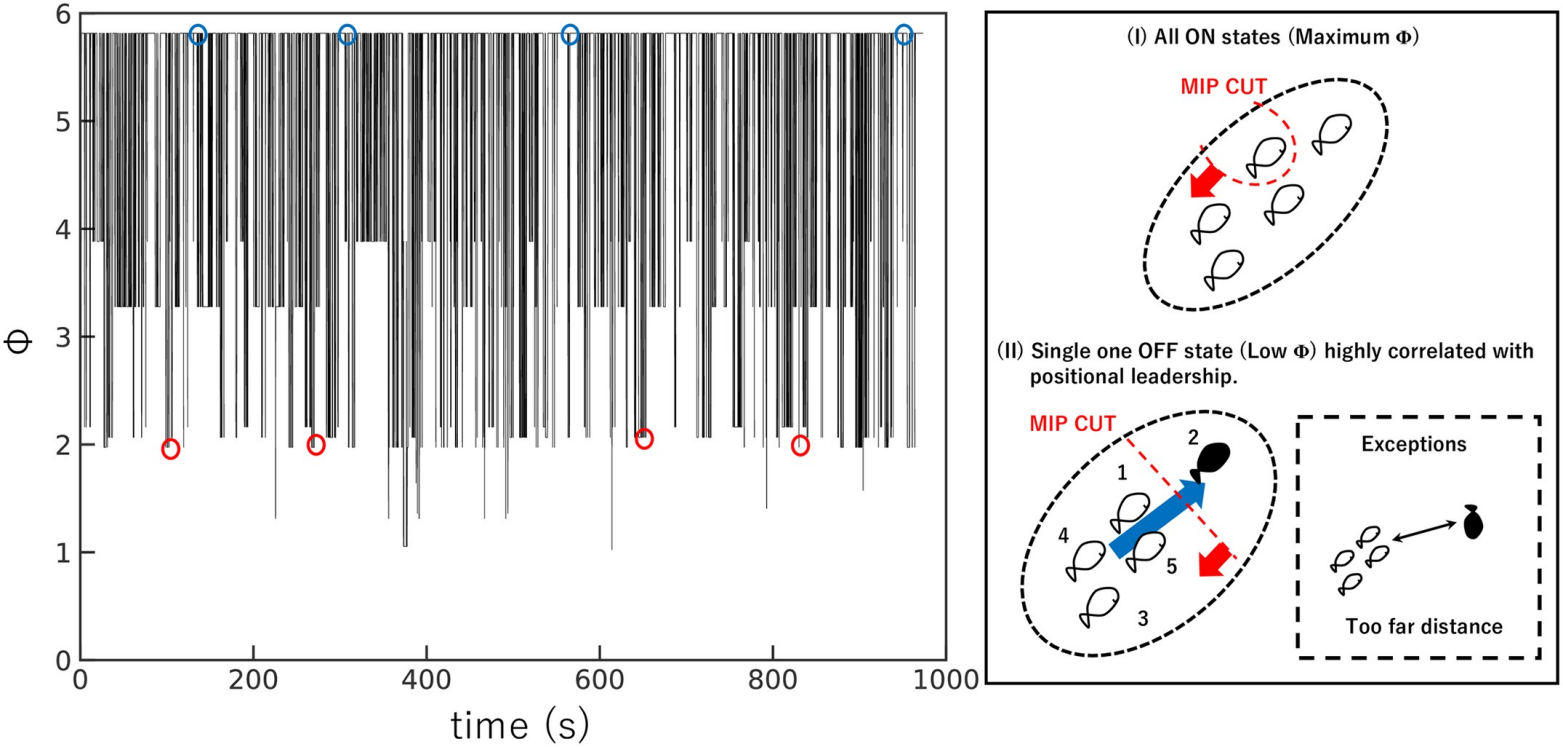
Example of a time series of Φ obtained from real fish data (*N* = 5). The reduction of Φ at various points indicates where leadership emerges in the group. The local parameter settings are distance = 700 *mm*, visual field = 1.6*π* rad, and turning rate = 0 rad/s, respectively. The illustrations on the right represent the example state of the fish (white: ON, black: OFF) and their minimum information partition (MIP). The blue circles on the left plot correspond to formation I on the right. The red circles on the left figure correspond to formation II on the right. This figure represents the cases where the positional leader (index 2) matches a single OFF state individual. The thick blue arrow is the average direction of the group. The dashed square indicates an exception, where a single OFF state individual is not the positional leader (this situation is rare: see Table 3). To enable interested readers to compute Φ using PyPhi, we list the transition probability matrix (TPM) in S1 Table.

Fig 6 is a representative example that summarises our descriptions. Fig 6 is a time series of Φ when the visual field is 1.6*π* and the distance is 700 mm. Intermittent dropping Φ values are observed. These minima correspond to instances when there is a single OFF state in the collective state, that is, 01111, 10111, and so on. In contrast, the highest Φ value corresponds to the all-ON collective state, that is, 11111. Although there are some exceptions for different parameter settings or data samples, all MIP cuts are locate between the OFF-state fish and the rest of the ON-state fish.

First, we show that a single OFF-state fish highly correlates with positional leadership (property (i)). In this study, the positional leader is defined as the individual in the first position with respect to the average group direction (see Fig 6). We can compute the rate at which single OFF-state individuals actually correspond with the positional leader. Let ***N*** and ***T*** be sets {1, 2, …, *N*} and {1, 2, …, *T*_*max*_}, respectively, where *T*_*max*_ is the maximum time for a given time step. Then, the single OFF state function is OFF_single_(*t*): ***T*** → ***N***⋃{0}. This function returns the index of the OFF-state individual when the collective state has only one OFF state; otherwise, it returns 0. The positional leadership function is P_Leader_(*t*): ***T*** → ***N***. This function selects the index of the positional leader.

$$\begin{array}{c} \text{MatchingRate}\left(\%\right)=\frac{\left|\{t|{\text{OFF}}_{\text{single}}\left(t\right)={\text{P}}_{\text{Leader}}\left(t\right),t\in T\}\right|}{\left|\{t|{\text{OFF}}_{\text{single}}\left(t\right)\in N,t\in T\}\right|}\times 100 \end{array}$$ (3)

The above equation (Eq (3)) indicates the frequency of occurrence of the OFF state individual as the positional leader. Table 2 shows that the matching rate increases substantially when the fish has a blind spot. If the fish has a maximum visual field, a single OFF-state individual does not correspond to the positional leader. In this context, it is appropriate to state that a positional leader emerges to raise the group integration on average when the group size is four or higher (an example of the actual positions for which single-OFF collective states occur are shown in S5 Fig).

**Table 2:** Φ values and match rates. The turning rate is fixed at 0 rad/s. All right-side values are average distance parameter values for all samples and range from 300 mm to 1000 mm. VF: visual field parameter; 〈Φ(*N*)〉_*S*_: mean Φ for a given set *S*, which is the set of single-OFF collective states such as {0111, 1011, 1101, 1110}; Φ_11…1_: Φ values for the all-ON collective state. The p-values for 〈Φ(*N*)〉_*S*_ and Φ_11…1_ were computed using the Wilcoxon signed-rank test. *MR*: match rate, computed by Eq (3) for a corresponding parameter set; *MR*_2*π*_: match rate computed by Eq (3) for fixed VF = 2*π*. The p-values for *MR* and *MR*_2*π*_ were computed using the Mann—Whitney U test. Values in bold correspond to the parameter settings for which the positional leader highly correlates with a single OFF-state fish in the group and where this fish also yields lower Φ values than those of Φ_11…1_.

	VF (rad)	〈Φ(*N*)〉_*S*_	Φ(*N*)_11…1_	p-value	MR (%)	MR_2*π*_ (%)	p-value
***N* = 4**	1.2*π*	0.169	0.200	< 0.001	95	13	< 0.001
	1.4*π*	0.421	0.470	> 0.1	96	13	< 0.001
	1.6*π*	0.676	0.592	< 0.001	96	13	< 0.001
	**1.8 *π***	**0.979**	**1.656**	**<0.001**	**94**	**13**	**<0.001**
	**1.9 *π***	**0.805**	**1.630**	**<0.001**	**83**	**13**	**<0.001**
***N* = 5**	1.2*π*	1.539	1.607	> 0.1	94	21	< 0.001
	**1.4 *π***	**2.884**	**3.831**	**<0.001**	**96**	**21**	**<0.001**
	**1.6 *π***	**2.918**	**4.586**	**<0.001**	**96**	**21**	**<0.001**
	**1.8 *π***	**2.199**	**4.617**	**<0.001**	**94**	**21**	**<0.001**
	**1.9 *π***	**1.614**	**3.446**	**<0.001**	**82**	**21**	**<0.001**

**Fig 7:**
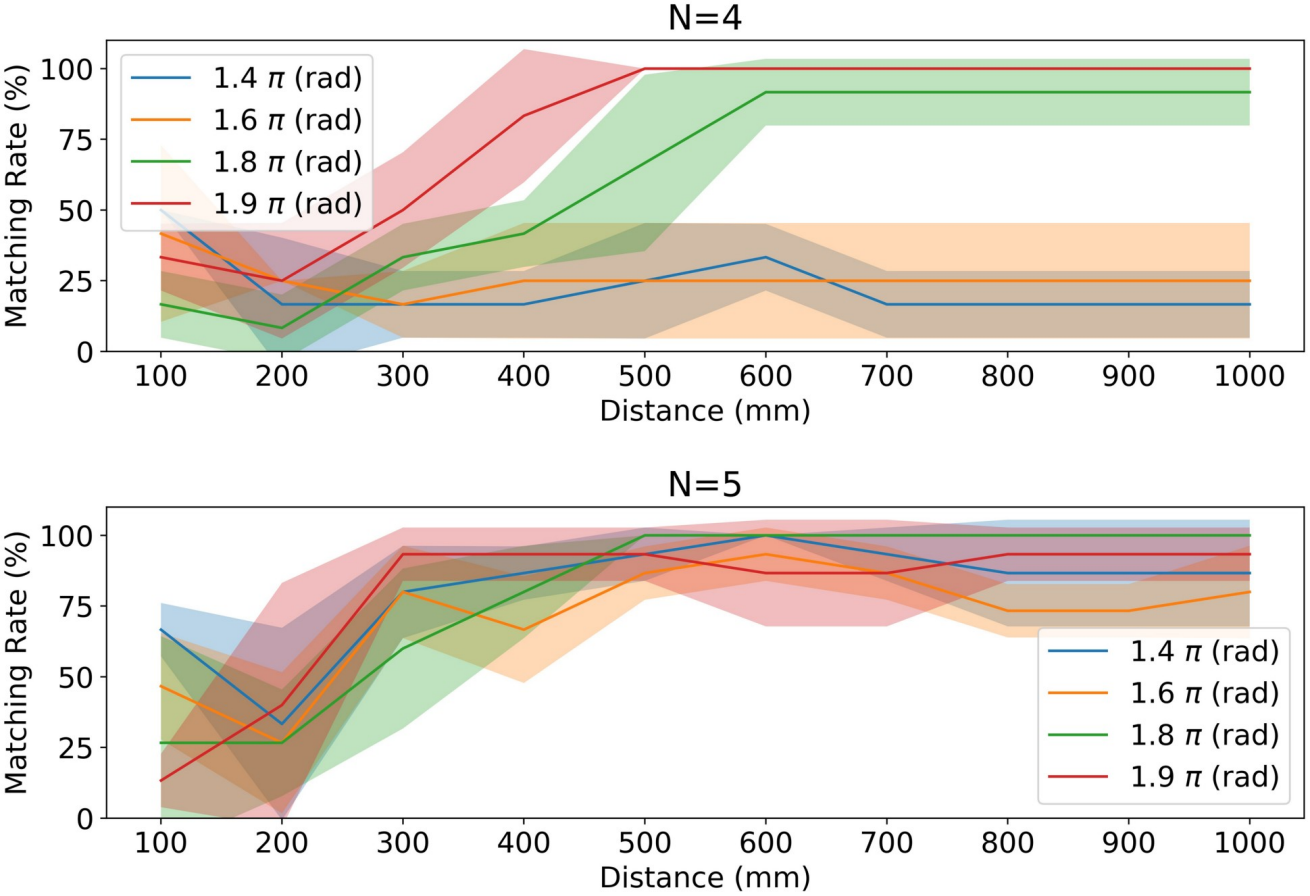
Match rates for a single OFF-state individual and the corresponding MIP cut between a single OFF state and all other ON states. We have {1} ⇏ {2, 3, 4, 5} or {2, 3, 4, 5} ⇏ {1} for the MIP cut when the collective state is 01111. The graph shows that the long-distance parameter discriminates the single OFF-state individual well according to the MIP cut for each visual field condition. Although a consistently low match rate exists for *N* = 4, these low visual field parameters are excluded by the leadership condition in Table 3. Note both graphs show an MIP cut without any self-loops because omitting them more clearly divides a single OFF-state individual and the rest of the members by an MIP cut (we have the same distribution as Fig 2 in S4 Fig). The version with self-loops is also listed in S6 Fig.

Next, we examine the information flow between a single OFF-state individual and the rest of ON-state group members in terms of MIP (property (ii)). According to the depictions in Figs 6 and 7 shows the matching rate for the MIP cut and a single OFF-state individual under almost all parameter settings (in particular, high-distance regions in Fig 7 show perfect matches). The MIP cut in the groups in Fig 7 can be expressed in PyPhi as {1} ⇏ {2, 3, 4, 5} or {2, 3, 4, 5} ⇏ {1}, where 1 is the index of a single OFF-state individual. The high correspondence of a single OFF-state individual and MIP cut locations suggests that the weakest link exists between the single OFF-state individual and the rest of the members (recall that the MIP cut lies where it causes the minimum information loss).

**Table 3:** Data summary.

*N*	Data index	Average distance (mm)	Average velocity (mm/s)	Error (S.D.)	Minimum distance (mm)	Total Time Steps
2	1	166.3	268.8	0.18	1.90	106,961
	2	90.67	271.68	0.23	0.10	99,431
	3	122.0	256.08	0.18	1.60	107,206
3	1	170.8	301.2	0.23	1.80	90,051
	2	159.1	343.2	0.14	1.83	83,654
	3	173.1	300.0	0.13	2.82	97,446
	4	132.0	240.0	0.19	1.67	93,931
4	1	164.3	270.72	0.14	1.18	106,327
	2	141.5	190.8	0.12	1.38	103,226
	3	114.9	148.56	0.38	1.83	98,126
5	1	143.8	259.92	0.28	0.79	102,895
	2	146.0	213.12	0.12	1.16	97,346
	3	143.7	259.2	0.28	1.44	92,116

S.D. = standard deviation.

To summarise our analysis, the single-OFF state individual satisfies two properties that we have listed: (i) the leader in the head position of the group as the positional leader (Table 2), (ii) there is MIP induced asymmetrical information flow among the leader and the rest of the members. The single-OFF state in high 〈Φ〉_*S*_ condition for four- and five-fish schools becomes the leader in terms of IIT (hereafter, we call this single OFF-state individual as IIT leadership). Note that this asymmetrical information flow represents a ‘feedforward’ information flow to or from the IIT leadership [61] because we have an MIP cut in both directions ({1} ⇏ {2, 3, 4, 5} or {2, 3, 4, 5} ⇏ {1}).

The different MIP cuts can be interpreted as follows. In the case of {1} ⇏ {2, 3, 4, 5}, the Φ value indicates that information flows from the rest of the members to a single OFF-state individual, but not so much from a single OFF-state individual to the others. In contrast, the reverse relation applies for {2, 3, 4, 5} ⇏ {1}. Taking into account the low Φ at a single OFF-state condition compared with all-ON state conditions (see Table 2), the information flow from or to a single OFF-state individual is weak compared with its inverse flow.

These facts lead us to consider that classification would be possible according to the two kinds of IIT leaderships using these asymmetrical information flows, i.e., one is passive leadership (the former instance, because most of the information flows from the other members) and the other is active leadership (the latter instance, because most of the information flows from the IIT leader). In the Discussion section, we provide a response to some anticipated objections to our interpretation.

#### Representative Φ values for all data samples

**Fig 8:**
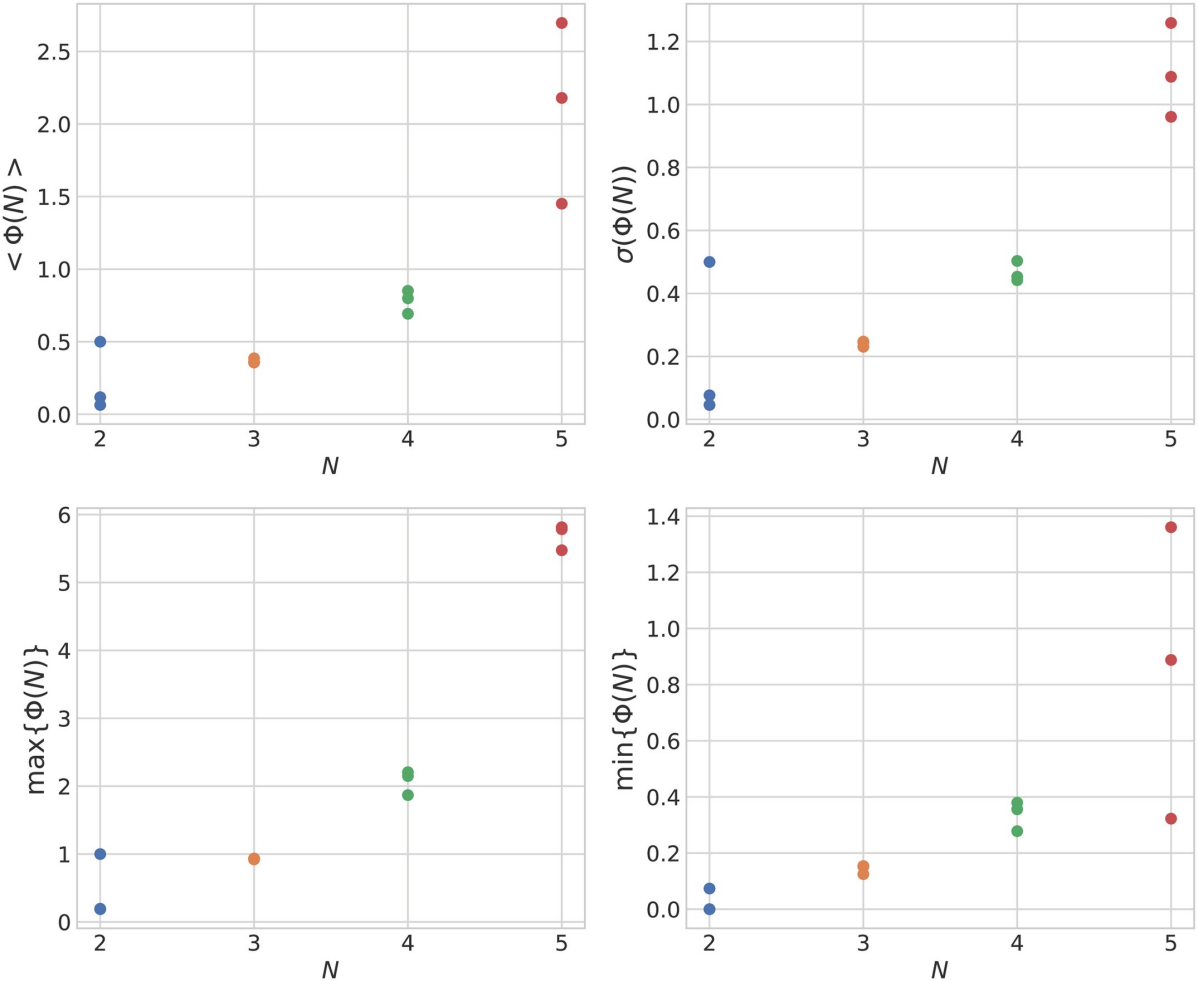
Mean values 〈Φ(*N*)〉, standard deviations *σ*(Φ(*N*)), max values max{Φ(*N*)}, and min values min{Φ(*N*)} for various fish group sizes. Groups *N* = 2, *N* = 4, and *N* = 5 have three samples and group *N* = 3 has four samples. Parameters were set using the peak values of Fig 2. The mean values and standard deviations of integrated information Φ increase as the size of the fish school (*N*) increases.

Finally, we examine the average 〈Φ(*N*)〉 values shown in Fig 2 in detail. In Fig 8, the peak cells in Fig 2 and S5 Fig (i.e. high average and high variance conditions) from each group size are as follows: (N = 2: D = 600 mm and VF = 2.0*π* rad, N = 3: D = 400 mm and VF = 2.0*π* rad, N = 4: D = 600 mm and VF = 1.8*π* rad, N = 5: D = 700 mm and VF = 1.6*π* rad. Here, D is the interaction radius and VF is the visual field. The turning rate is fixed at 0 rad/s). When increasing 〈Φ(*N*)〉, the maximum Φ also increases (recall that an *n*-fish school has 2^n^ Φs with respect to its collective states). This maximum Φ corresponds to the all-ON collective states for *N* = 4 or 5 (one example for *N* = 5 is used in Fig 6). The susceptibility is a measure of fluctuation or the response of an extensive property (such as the order parameter) to a small external perturbation that introduces some variations in the intensive property. Some researchers suspect that the susceptibility of integrated information defined by the standard deviation (or variance) of Φ relates to the given system’s autonomous properties [67, 85]. Intuitively, a high standard deviation (or variance) indicates that the system contains both low group integration states and high group integration states. We show the variance distribution in S7 Fig for the parameter settings in Fig 2. Here, we only point out that the peak of 〈Φ(*N*)〉 and variance of Φ do not always agree with each other.

### IIT 3.0 on the Boids model

For comparison, simulated trajectories based on the Boids model [88] were analysed in the same manner as the trajectories of the real fish school. The Boids model was developed by Reynolds [95] and its complex and realistic-looking behaviour of a group of agents as a whole is determined entirely by the local interactions of individual agent choices based on a set of simple rules: repulsion, alignment, and attraction. In this study, *N* agents with position vectors *x*_*i*_ and unit direction vectors *v*_*i*_ were simulated in continuous two-dimensional space (3, 000 × 2, 500) (the size of the experimental fish tank). Time was discretised into *t* computational time steps with a regular spacing Δ*t* = 0.05. When there are *n*_*r*_ agents in the neighbourhood of agent *i*, the following rules for repulsion, alignment, and attraction were applied to update the variables of the agents at each *t*:

$$\begin{array}{c} d_{r}\left(t+\Delta t\right)=-C\sum_{j\neq i}^{n_{r}}\frac{r_{ij}\left(t\right)}{|r_{ij}\left(t\right)|} \end{array}$$ (4)

$$\begin{array}{c} d_{o}\left(t+\Delta t\right)=C\sum_{j=1}^{n_{j}}\frac{v_{j}\left(t\right)}{|v_{j}\left(t\right)|} \end{array}$$ (5)

$$\begin{array}{c} d_{a}\left(t+\Delta t\right)=C\sum_{i\neq j}^{n_{a}}\frac{r_{ij}\left(t\right)}{|r_{ij}\left(t\right)|} \end{array}$$ (6)

where *n*_*r*_ = {*j* ∣ ***r***_*ij*_(*t*) ≤ *R*}, *n*_*o*_ = {*j* ∣ ***r***_*ij*_(*t*) ≤ *O*}, *n*_*a*_ = {*j* ∣ *O* ≤ ***r***_*ij*_(*t*) ≤ *A*}, and $r_{ij}=\frac{\left(x_{j}-x_{i}\right)}{\left|(x_{j}-x_{i})\right|}$ is the unit vector in the direction of neighbour *j*. The above rules were summed and averaged with additive Gaussian noise to determine the trajectories of the agents. The variables were updated synchronously. The parameters used in this simulation are shown in Table 4.

**Table 4:** Summary of model parameters.

*N*	*R*	*O*	*A*	*v*	Error (S.D.)
2	10	120	∞	11.1	0.20
3	10	120	∞	12.4	0.17
4	10	120	∞	8.47	0.21
5	10	120	∞	10.2	0.23

S.D. = standard deviation.

**Fig 9:**
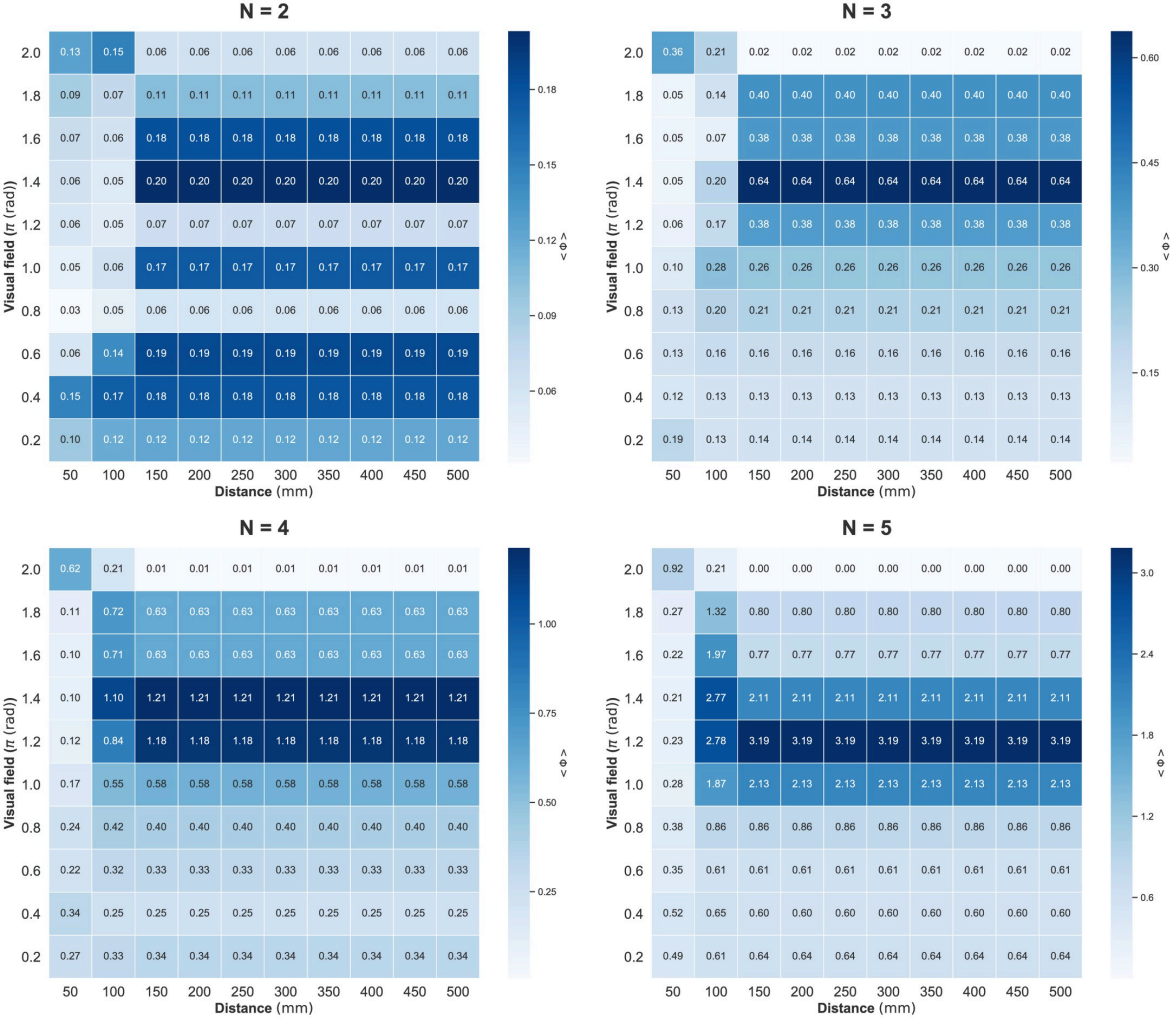
Values of mean 〈Φ〉 for the Boids model for 10 samples each. Distance vs. visual field heat map.

The trajectories of the Boids model and real fish showed the same complexity (S8 Fig); however, the 〈Φ〉 heat maps for the Boids model exhibited very different patterns (Fig 9). The values of Φ for the Boids model were generally larger across the different parameter settings and did not seem to be as sensitive to the distance parameters.

The dynamics of the Boids model and real fish appeared to be very similar; however, the Φ values of the Boids model have large variances and lacked the discontinuity in their patterns between *N* = 3 and *N* = 4 (the distribution of MI and TE are resemble to real ones S9 Fig). In addition, there were substantial differences, especially when comparing the case of *N* = 2. When *N* = 2, the Boids model yielded wide distributions of Φ; in contrast, the real *N* = 2 had very narrow and susceptible peaks.

**Fig 10:**
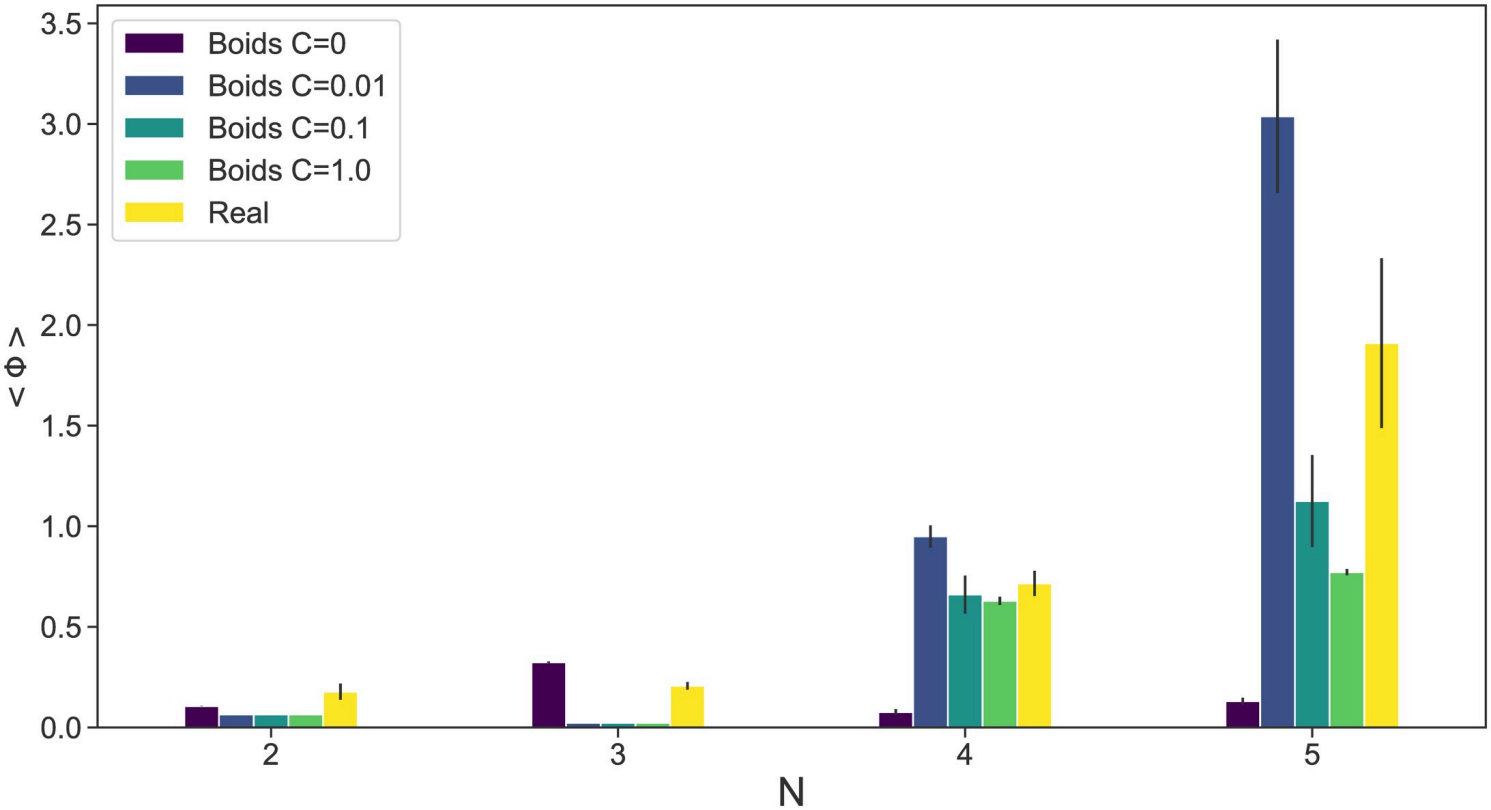
Comparison of the Φ values for certain parameter settings. The values of Φ_Boids_ were calculated at the parameter configurations that maximise the real fish 〈Φ〉_Real_ and averaged for 10 simulations. Error bars indicate the standard deviations.

The 〈Φ〉 values were also calculated in the same manner and averaged for 10 simulations each. We were interested in any difference in the patterns of 〈Φ〉 in the parameter space. We picked up the 〈Φ〉 values for each of the following parameter configurations of the Boids model that maximise the 〈Φ〉 values for the real fish school: Distance = 400 mm, Visual field = 2.0*π* rad for *N* = 2; Distance = 400 mm, Visual field = 2.0*π* rad for *N* = 3; Distance = 800 mm, Visual field = 1.8*π* rad for *N* = 4; and Distance = 700 mm, Visual field = 1.6*π* rad for *N* = 5. One parameter setting could maximise one 〈Φ〉 value but not the other (Fig 10). The 〈Φ〉 distributions of the Boids model were completely different from those of real fish.

**Fig 11:**
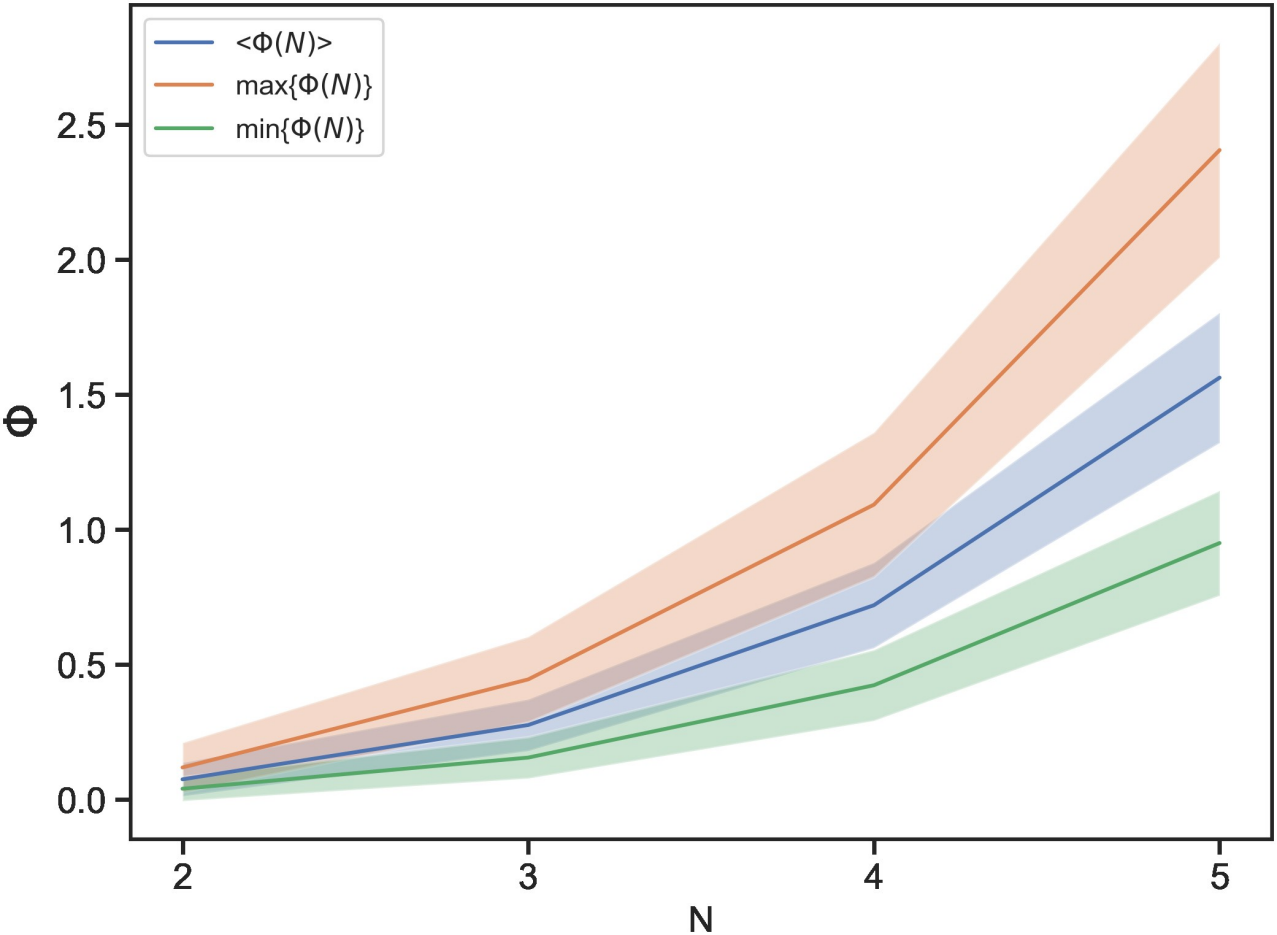
〈Φ〉, maxΦ(*N*), and minΦ(*N*) values of time-homogeneous Markov chains with different values of *N*. The Φ values were calculated for randomly generated 100 transition probability matrices (TPMs). The shadows indicate the standard deviations.

To assess whether the maximisation of the integrated information is essentially dependent on the size of the system or fish behaviour, we investigated Boids models with reduced coupling strength (*C* = 1.0, 0.1, 0.01, 0) (see S10 and S11 Figs). We expected a coupling strength value of *C* = 0 to lead to a 〈Φ〉 of 0 for all values of *N*; however, 〈Φ〉 is non-zero and increases as *N* grows. We suspect this is possibly due to size effects as well as boundary effects, both of which increase as *N* grows. Regarding the size effects, the Φ values of time-homogeneous Markov chains, which are probabilistic systems with random connexions, increase with size (Fig 11). Comparing the real fish schools and the Markov models, it is remarkable that the real fish schools have larger standard deviations for Φ(*N*) (Fig 8) than those of the Markov chains (*σ*(Φ(2)) = 0.03 ± 0.04, *σ*(Φ(3)) = 0.09 ± 0.03, *σ*(Φ(4)) = 0.19 ± 0.06, and *σ*(Φ(5)) = 0.36 ± 0.07). The larger variances of Φ in the real fish schools imply that the real fish have more variety and diversity in their states.

Another factor that raised the Φ(*N*) values for *C* = 0 systems was the use of the binary parameter format, even though the fish move continuously. The Φ(*N*) values would be 0 if the fish were placed randomly at all time steps so that there were no interactions (no boundaries). For example, for a visual field of 2*π*, if one fish is in the visual field of another, the reverse is also true; i.e., the state 1000 is impossible, only 1100 and similar patterns can exist. In such conditions, asymmetric interactions in which one fish is in the ON state and the other remains in the OFF state can never occur. This heterogeneity in TPM results in the non-zero Φ. We call this the boundary effect. Interestingly, the weak interactions *C* = 0.01 that have different distributions compared with *C* = 0 might suppress the boundary effects, resulting in a relatively lower 〈Φ〉. In fact, the model with weaker interactions *C* = 0.01 actually has a distribution that is very similar to that of a real fish school S10 Fig). While the averaged 〈Φ〉 depends on coupling strength, we emphasise that we must compare the distributions to properly discuss the effect of behaviour on integrated information S11 Fig). A clear change in the distributions of 〈Φ〉 occurs when coupling strength is reduced.

## Discussion

Although IIT was first proposed to quantify the degree of consciousness from brain activity, interest in IIT seems to have gradually shifted to using it to determine a system’s causal structure itself [54, 80, 83]. More precisely, the researchers’ concern for the causal structure of the autonomous system was hidden under their ambitious project to quantify human consciousness. This move is commendable because, by doing so, IIT researchers have paved the way for a new measure for any general living system. The concept of causal structure is not used to determine information flow among agents, as in TE. Instead of measuring ‘what the system does’, IIT can measure ‘what the system is’. By imposing an intervention on the system, we can estimate how the system reacts to such external perturbations. Pearl, known as the founder of causal inference, and other researchers have also applied almost the same perturbation method to causal networks [62]. The MIP cut makes integrated information stand apart from other information measures like MI or TE [96].

In this study, we applied IIT 3.0 to real fish schools and compared the results with those for other measures (MI and TE) and another model (Boids) under the same conditions. The simple phenomenon that IIT suggests is an increase in 〈Φ(*N*)〉 values with group size in real and Boids-model fish schools. Furthermore, the coupling strength of the Boids model also changes the 〈Φ(*N*)〉 values. This result agrees well with our intuition for the group integration using IIT 3.0. Our experiment indicates that a relatively weak interaction happens in real fish schools.

From the Φ distributions for all possible combinations of parameters, we found a discontinuity between three- and four-fish schools: the leadership raises the degree of integration above four but not below three. This discontinuity between three and four fish not only exists in the IIT of real fish but also in the IIT of the Boids model. Furthermore, we found that MI and the sum of TE also fail to capture these differences. Note that IIT discriminates between three- and four-fish groups, which is a comparison that is rarely considered in the context of collective animal behaviour, although there are some studies that suggest a difference between two- and three-fish groups in terms of each fish’s interactions with others (i.e. a difference in ‘what the system does’ from an extrinsic perspective) [11, 41, 42].

Finally, we comment on the IIT leadership in the group. The definition of IIT leadership is a single OFF-state in the collective state (e.g. 11110 or 01111) which satisfies the following: (i) matching well with positional leadership (the leader of the group is the head of its group) and (ii) the MIP cut is located between a single OFF-state individual and the rest of the group members.

Prior to providing details of leadership discussion, two objections might still be anticipated. One is that if the MIP cut indicates the weakest connection (interaction) among the leader and the rest of its members, does it mean that the low 〈Φ〉 suggests a relatively independent relation between the leaders and the rest of the members? As discussed earlier, MIP cut is unidirectional and not bi-directional. The low 〈Φ〉 values induced MIP provide no information about opposite causal flows are also weak. Adding to this point, the 〈Φ〉 values of single OFF-state collective states (01111) in five-fish schools show higher values compared with all one states (1111) in four-fish school as in Table 2. Although the group integrity is low when the five-fish schools are at a single OFF-state collective states, their integrity is still sufficiently higher than the highest integrity in four-fish school. Therefore, the first objection can be rejected.

The next objection is (ii) If the opposite information flows are as weak as the MIP cut ones (i.e. both flows have the same weakness), it is hard to suggests the feed-forward relation as we discussed. This objection is difficult to answer because PyPhi provides no information about opposite direction information flows. However, if this assumption is correct, we consider that it is not so critical that our interpretation is incorrect. This assumption is possible when we suppose that the weak interactions never contribute to the group dynamics. This assumption is not always true, especially in a dynamical system. For example, some researchers suggest that the weak interaction provides the stabilisation/destabilisation to the ecosystem [97–99]. Even if small perturbations are added to the system, the degree of perturbations can work as the system’s parameters and make the system qualitatively different through bifurcation. Therefore, the weak interaction itself never suffices to spoil our interpretation of the leadership induced MIP.

The group leader detection result using IIT 3.0 suggests two remarkable propositions for understanding group behaviour. One is that our natural intuition of leadership (i.e. positional leadership) is basically correct under the given causal structure. As in [61], low Φ values represent that the system is a feed-forward system (more precisely, it has a weak recurrent interaction structure). In this sense, the information flow is uni-directional because it is in a low Φs system. Therefore, the low Φs of single-OFF collective states have much lower recurrent interactions than the all-ON collective states.

The lack of recurrent interactions also gives a new insight into the discontinuity between three- and four-fish schools. If the visual field is not 2*π* for two- and three-fish schools, their interactions are almost completely feed-forward in all collective states. The visual field asymmetry never contributes to recurrent interactions. As a result, the leadership is not explicitly discriminated by IIT 3.0; however, if the visual field is not 2*π* for fish schools with four or more fish, their interaction structure becomes more differentiated. Although Φ values are low under the single-OFF condition, the high Φ values of the all-ON condition suggest that a profoundly organised recurrent interaction emerges. Therefore, the discontinuity between three- and four-fish schools can be interpreted in terms of their different information structures.

The other proposition is that the MIP may detect the division of roles in collective behaviour (i.e. the weakest link divides the group into related aggregations). If we can extend this method to larger fish schools, IIT 3.0 would be able to determine the hidden division of the roles of a given collective behaviour. Furthermore, this role may not be revealed by other information measures.

In this study, we avoided going deeper into the problem of timescale (we only used a relatively small timescale, which is roughly equal to a general fish’s reaction time). Over longer timescales, other patterns of continuity and discontinuity may be found. Increasing the number of individuals may also give other results. However, the present practical computational limit of IIT 3.0 is around seven or eight individuals or neurons [72]. Hence, some approximations will be needed to implement further analysis. Another area we did not address is the network structure. We assumed an all-connected network including self-loops in this study because all fish come into contact with each other throughout the event. As we hinted in S6 Fig, the network structures that exclude self-loops might be more suitable for finding the division in terms of MIP. Furthermore, some studies suggest that the network structure of real schools of fish is radically different from the Boids model and that they instead create a stable network called the *α*-lattice [100, 101]. This type of network may prevent the increases in Φ observed in the Boids model.

## Materials and methods

### Ethics statement

This study was carried out in strict accordance with the recommendations in the Guide for the Care and Use of Laboratory Animals of the National Institutes of Health. The protocol was approved by the Committee on the Ethics of Animal Experiments of the University of Tsukuba (Permit Number: 14-386). All efforts were made to minimize suffering.

### Computation of Φ

All computations, in this paper, were performed using the PyPhi software package with the CUT_ONE_APPROXIMATION option for Φ.

### Experimental settings

We studied *ayus* (*Plecoglossus altivelis*), also known as sweetfish, which live throughout and are widely farmed in Japan. Juvenile *ayus* (approximately 7-14 *cm* in body length) display typical schooling behaviour, though adult *ayus* tend to show territorial behaviour in environments where fish density is low. We purchased juveniles from Tarumiyoushoku (Kasumigaura, Ibaraki, Japan) and housed them in a controlled laboratory. Approximately 150 fish lived in a 0.8 *m*^3^ tank of continuously filtered and recycled fresh water with a temperature maintained at 16.4°C, and were fed commercial food pellets. Immediately before each experiment was conducted, randomly chosen fish were separated to form a school of each size and were moved to an experimental arena without pre-training. The experimental arena consisted of a 3×3*m*^2^ shallow white tank. The water depth was approximately 15 *cm* so that schools would be approximately two-dimensional. The fish were recorded with an overhead grey-scale video camera (Library GE 60; Library Co. Ltd., Tokyo, Japan) at a spatial resolution of 640 ×480 pixels and a temporal resolution of 120 frames per second.

### Data summary

The data are summarised in Table 3.

Parameters are the key to determining the dynamics of the model. In the present study, the model parameters were set to simulate the real experimental data shown in Table 3. The average distances were approximately 80 to 140 mm, so we set *O* = 120 mm, *R* = 10 mm (the body length), and *A* = ∞. Thus, the fish schools should not part less than 140 mm. This setting was necessary for the school to become separated by the boundary conditions, which mimic and reflect the real data. The amplitudes of the noise was set to be proportional to the average angle change so each agent would have a different level of noise. The parameter settings are shown in Table 4.

### Definition of ON and OFF states for each parameter

We define a function for each parameter that returns either 0 (OFF) or 1 (ON) for a given input value. Generally, we denote a function as $F_{i}^{t}\left(\cdot \right)$, where *F* is the name of the function, *i* is the index of the individual and *t* is the time. The arguments of the function can be either in the position vectors ***x***_*i*_(*t*) or the velocity vectors ***v***_*i*_(*t*) of each individual at time *t*. In general, the dimensions of these vectors are *d* ≤ 3; the experimental setup used here gives *d* = 2. The number of individuals is *n*.

#### Parameter settings

- Distance function $D_{i}^{t}\left(x_{1}\left(t\right),x_{2}\left(t\right),\cdots ,x_{n}\left(t\right)\right)$: $R^{d}\times R^{d}\times \cdots \times R^{d}\overset{}{\to }\left\{0,1\right\}$
  For each individual *i* we obtain a set $S_{i}^{t}=\left\{j|d\left(x_{i}\left(t\right),x_{j}\left(t\right)\right)<\zeta ,j\neq i\right\}$ of all other individuals within a specified distance *ζ*. Here *d*(***x***, ***y***) gives the Euclidean distance between ***x*** and ***y***. Then, $D_{i}^{t}\left(x_{1}\left(t\right),x_{2}\left(t\right),\ldots ,x_{n}\left(t\right)\right)=1$ when $|S_{i}^{t}|>0$ and is 0 otherwise, where |*S*| denotes the number of elements of a set *S*.
- Visual field function $B_{i}^{t}\left(x_{1}\left(t\right),x_{2}\left(t\right),\ldots ,x_{n}\left(t\right),v_{1}\left(t\right),v_{2}\left(t\right),\cdots ,v_{n}\left(t\right)\right):R^{d}\times R^{d}\times \cdots \times R^{d}\overset{}{\to }\left\{0,1\right\}$
  For each individual we form the set $O_{i}^{t}=\{j|\text{arg}(v_{i}(t),x_{i}(t)-x_{j}(t))<\eta ,j\neq i$} of all other individuals whose velocity vectors point in a direction within an angle *η* of that of the focal individual. The function arg(***x***_1_(*t*), ***x***_2_(*t*)) gives the angle between two vectors. Then, $B_{i}^{t}\left(x_{1}\left(t\right),x_{2}\left(t\right),\ldots ,x_{n}\left(t\right),v_{1}\left(t\right),v_{2}\left(t\right),\cdots ,v_{n}\left(t\right)\right)=1$ when $|O_{i}^{t}|>0$ and is 0 otherwise.
- Turning rate function $T_{i}^{t}\left(v_{i}\left(t\right),v_{i}\left(t-\Delta t\right)\right):R^{d}\times R^{d}\overset{}{\to }\left\{0,1\right\}$
  The turning rate function returns 1 when an individual’s turning rate exceeds a specified threshold*δ*. That is, $T_{i}^{t}\left(v_{i}\left(t\right),v_{i}\left(t-\Delta t\right)\right)=1$ when arg(***v***_*i*_(*t*), ***v***_*i*_(*t* − Δ*t*)) ≥ *δ* and is 0 otherwise. The time step used in this paper is Δ*t* = 0.05, Δ*t* = 0.1 or Δ*t* = 0.2 s.
  To obtain the states of the fish school, we take a conjunction of this result, that is, $D_{i}^{t}\left(x_{1}\left(t\right),x_{2}\left(t\right),\cdots ,x_{n}\left(t\right)\right)\wedge B_{i}^{t}\left(v_{1}\left(t\right),v_{2}\left(t\right),\cdots ,v_{n}\left(t\right)\right)\wedge T_{i}^{t}\left(v_{i}\left(t\right),v_{i}\left(t-\Delta t\right)\right)$ for each individual *i*. The conjunction is given as ∧: {0, 1}^2^ → {0, 1} where 1 ∧ 1 = 1 and is 0 otherwise. Thus the state of each individual *i* at time *t* is *s*_*i*_(*t*; *ζ*, *η*, *δ*) ∈ {0, 1} which depends on the triplet of parameter values (*ζ*, *η*, *δ*). The state of the school at time *t* is then a vector *s*(*t*) = (*s*_1_(*t*), *s*_2_(*t*), …, *s*_*n*_(*t*)) ∈ {0, 1}^*n*^, where the parameter dependence has been omitted for simplicity.

### Short summary of integrated information Φ

Integrated information theory models a system *S* by the discrete time multivariate stochastic process

$$\begin{array}{c} p(X_{0},X_{\Delta t},\ldots ,X_{t},X_{t+\Delta t},\ldots ,X_{T}) \end{array}$$ (7)

which fulfils the Markov property

$$\begin{array}{c} p\left(X_{0},X_{\Delta t},\ldots ,X_{t},X_{t+\Delta t},\ldots ,X_{T}\right)=p\left(X_{0}\right)\prod_{t=\Delta t}^{T}p\left(X_{t}|X_{t-\Delta t}\right) \end{array}$$ (8)

Such a discrete dynamical system *S* is defined by a directed graph of interconnected nodes (in this study, we assumed a complete graph.) and its TPM. The TPM specifies the conditional probability distribution *p*(*X*_*t*_ ∣ *X*_*t*−Δ*t*_). Each state vector *X*_*t*_ comprises binary variables $x_{t_{i}}$, *i* = 1, 2, …, *n*(*n* ∈ *N*).

A joint distribution *p*_cause−effect_ is defined as

$$\begin{array}{c} p_{\text{cause}-\text{effect}}\left(X_{t-\Delta t},X_{t}\right)≔p_{u}\left(X_{t-\Delta t}\right)p_{\text{effect}}\left(X_{t}|X_{t-\Delta t}\right) \end{array}$$ (9)

The marginal distribution *p*_*u*_(*X*_*t*−Δ*t*_) is a uniform distribution to give the maximum entropy distribution.

From the joint probability above, the backward transitional probability distribution

$$\begin{array}{c} p_{\text{effect}}\left(X_{t-\Delta t}|X_{t}\right)≔\frac{p_{\text{cause}-\text{effect}}\left(X_{t-\Delta t},X_{t}\right)}{\sum_{X_{t-\Delta t}}p_{\text{cause}-\text{effect}}\left(X_{t-\Delta t},X_{t}\right)} \end{array}$$ (10)

and the forward transitional probability distribution

$$\begin{array}{c} p_{\text{cause}}\left(X_{t}|X_{t-\Delta t}\right)≔p\left(\left(X_{t}|X_{t-\Delta t}\right)\right) \end{array}$$ (11)

are constructed and referred to as the *cause repertoire* and the *effect repertoire* of state *X*_*t*_, respectively. The *cause repertoire* and the *effect repertoire* are calculated for a set of nodes within the subsystem, or a *mechanism* *M* ⊆ *S*, over another set of nodes within the subsystem, or a *purview* of the mechanism.

After assessing the information of a mechanism over a purview, we next consider its *integrated information φ*_cause−effect_ of a set of system elements in a state X. The integrated information is defined as

$$\begin{array}{c} \phi_{\text{cause}-\text{effect}}≔\text{min}\left\{\phi_{\text{effect}},\phi_{\text{cause}}\right\} \end{array}$$ (12)

$$\begin{array}{c} \phi_{\text{effect}}≔\underset{i\in I}{\text{min}}\left\{D(p_{\text{effect}}\|p_{\text{effect}}^{\left(i\right)})\right\} \end{array}$$ (13)

$$\begin{array}{c} \phi_{\text{cause}}≔\underset{i\in I}{\text{min}}\left\{D(p_{\text{cause}}\|p_{\text{cause}}^{\left(i\right)})\right\} \end{array}$$ (14)

where the system is decomposed in all possible ways into *I*.

The integrated information *φ* is assessed by quantifying the extent to which the cause and effect repertoires of the mechanism–purview pair can be reduced to the repertoires of its parts. The amount of irreducibility of a mechanism over a purview with respect to a partition is quantified as the divergence between the unpartitioned repertoire *p* and the partitioned repertoire *p*^(*i*)^. The partition that yields the minimum irreducibility is called the *minimum-information partition* (MIP). The integrated information *φ* of a mechanism-purview pair is defined as the divergence between the unpartitioned repertoire and the repertoires partitioned by the MIP. The maximum *φ* value is then searched for over all possible purviews to determine the *maximally-irreducible cause* and *maximally-irreducible effect* specified by a mechanism.

$$\begin{array}{c} \varphi_{\text{cause}}^{\text{max}}≔\underset{j\in C}{\text{max}}\left\{\varphi_{\text{cause}}^{j}\right\},\varphi_{\text{effect}}^{\text{max}}≔\underset{j\in C}{\text{max}}\left\{\varphi_{\text{effect}}^{j}\right\} \end{array}$$ (15)

where *C* = 2^*N*^ − 1. (In this paper, we adopted a ‘cut one’ approximation that only evaluates 2*N* bipartitions severing the edges from a single node to the rest of the network).

The *φ* value of the concept as a whole or the maximally integrated cause–effect information is the minimum of the maximally integrated cause information *φ*_cause_ and maximally integrated effect information *φ*_effect_.

$$\begin{array}{c} \varphi_{\text{cause}-\text{effect}}^{\text{max}}≔\text{min}\left\{\varphi_{\text{cause}}^{\text{max}},\;\varphi_{\text{effect}}^{\text{max}}\right\} \end{array}$$ (16)

If the mechanism’s maximally-irreducible cause has *φ*_cause_ > 0 and its maximally-irreducible effect has *φ*_effect_ > 0, (equivalently $\phi_{\text{cause}-\text{effect}}^{\text{max}}>0$), then the mechanism is said to specify a *concept*.

We then compute the *cause–effect structure*, which is the set of all concepts specified by the subsystem characterising all of the causal constraints intrinsic to the physical system, by simply iterating the computation of the concepts over all mechanisms M∈P(S), where P(S) is the power set of subsystem nodes.

Integrated conceptual information Φ (also known as *big phi*), which is a measure of the system’s strong/integration irreducibility, is assessed by partitioning the set of elements into subsets with unidirectional cuts. The unidirectional bipartitions *P*_→_ = {*S*^(1)^;*S*^(2)^} of physical system *S* are performed by partitioning the subsystem into two parts *S*^(1)^ and *S*^(2)^ and cutting the edges from one part *S*^(1)^ to another *S*^(2)^ (the connections are substituted with noise). We then calculate the cause–effect structure of the partitioned system $C(S^{P_{\to }})$ and compare it to *C*(*S*) to evaluate the difference made by the partition. MIP, which is a search over all possible directed partitions, is then performed to identify the one that makes the least difference to the cause–effect structure. Integrated conceptual information Φ measures the irreducibility of a cause–effect structure by quantifying the difference the MIP makes to the concepts and their *φ* values of the system.

$$\begin{array}{c} \Phi =\underset{P_{\to }}{\text{min}}\;D(C\left(S\right),C\left(S^{P_{\to }}\right)) \end{array}$$ (17)

The difference *D* between two cause–effect structures is evaluated by an extended version of the earth mover’s distance, which is the cost of transforming one cause–effect structure *C*(*S*) into another $C(S^{P_{\to }})$ in concept space.

## Supporting information

S1 Fig Heat map of 〈Φ〉 for the rest of the parameter settings in Fig 2, where Δ*t* = 0.05 s. (PDF)

S2 Fig Heat map of 〈Σ*φ*〉 in Fig 2 for the other parameter settings. (PDF)

S3 Fig Heat map of the mean 〈Φ〉 for several parameter settings in Fig 2, where Δ*t* = 0.10 s. (PDF)

S4 Fig Heat map of the mean 〈Φ〉 of main complex and no self-loop condition for several parameter settings in Fig 2, where Δ*t* = 0.05 s. (PDF)

S5 Fig Snapshot of the trajectory at *t* = 100, *t* = 225, and *t* = 763 when the IIT leadership emerges in Fig 6. The head of the group corresponds to the IIT leader.
(PDF)

S6 Fig Match rate of the IIT leader and the MIP cut with self-loops. (JPG)

S7 Fig Mean *σ*^2^(Φ) in Fig 2. (PDF)

S8 Fig Comparison of trajectories of real fish and results of the Boids model for *T* = 20, 000 time steps. (PDF)

S9 Fig Heatmaps of the mean MI and mean TE for Boids model results with C = 1.0. (PDF)

S10 Fig Heatmaps of 〈Φ〉 for Boids model results with different coupling strengths. (PDF)

S11 Fig Comparison of the averaged 〈Φ〉. The values of Φ were averaged over the distance–visual field parameter space and again averaged for 10 simulations and all real data. Error bars indicate the standard deviations.
(PDF)

S1 Table Transition probability matrix (TPM) used in Fig 6. (XLSX)
