# Supplementary figures and images for: Finding continuity and discontinuity in fish schools via integrated information theory
Source: PLoS One. 2020 Feb 27;15(2):e0229573. doi: 10.1371/journal.pone.0229573 (PMC7046263; doi:10.1371/journal.pone.0229573)

Distance - Turning Rate : fixed FV =  $2.0\pi$  rad

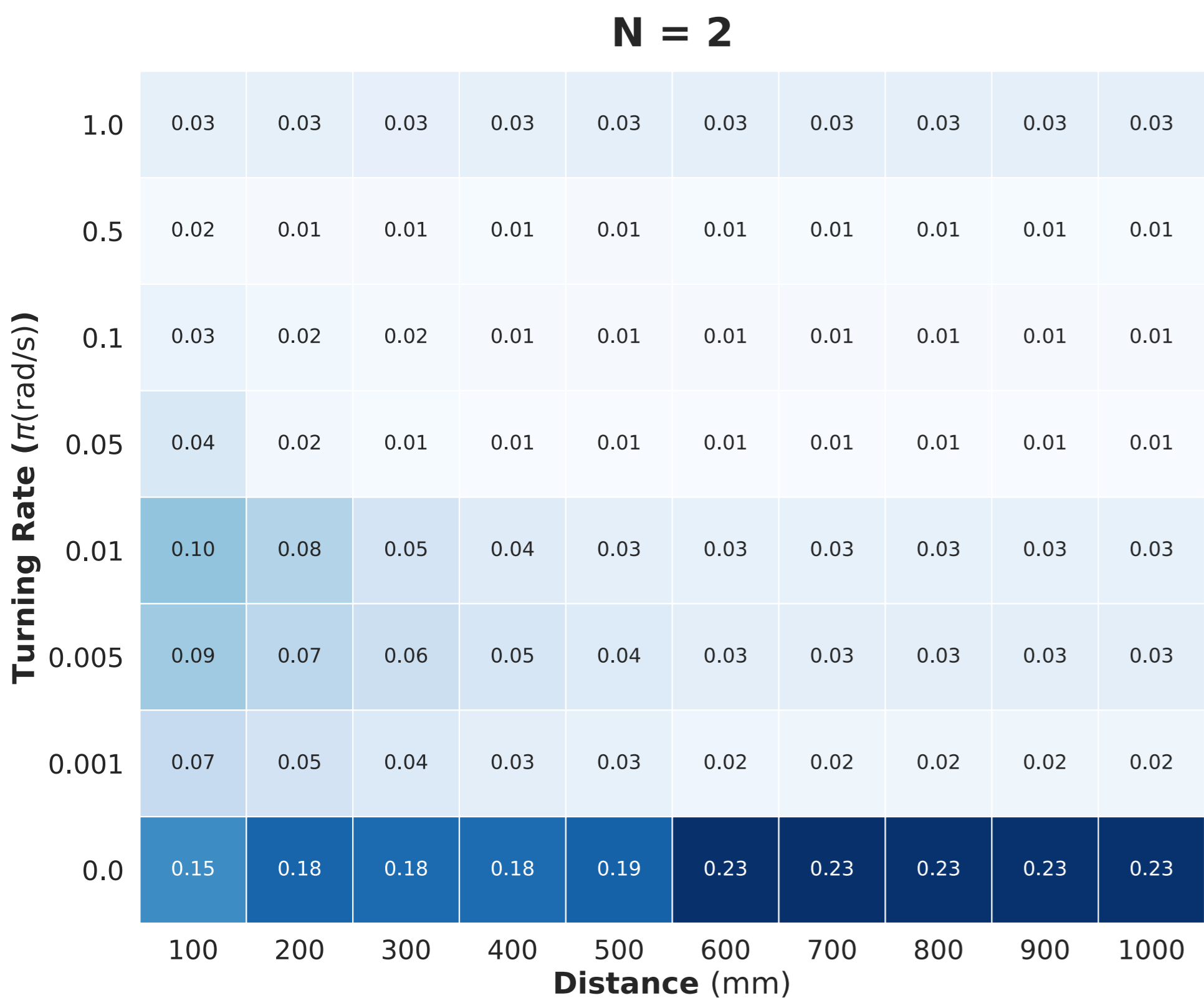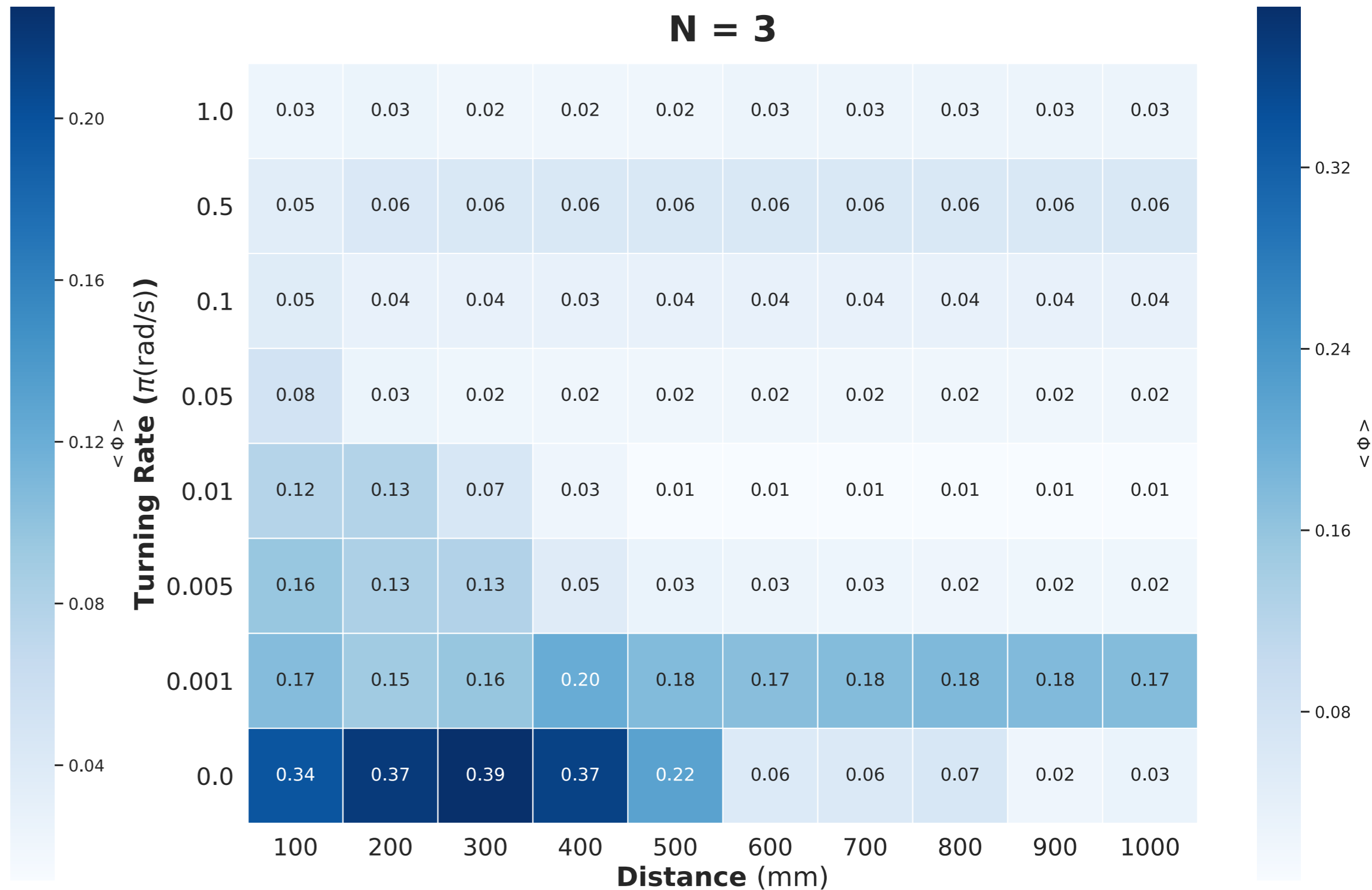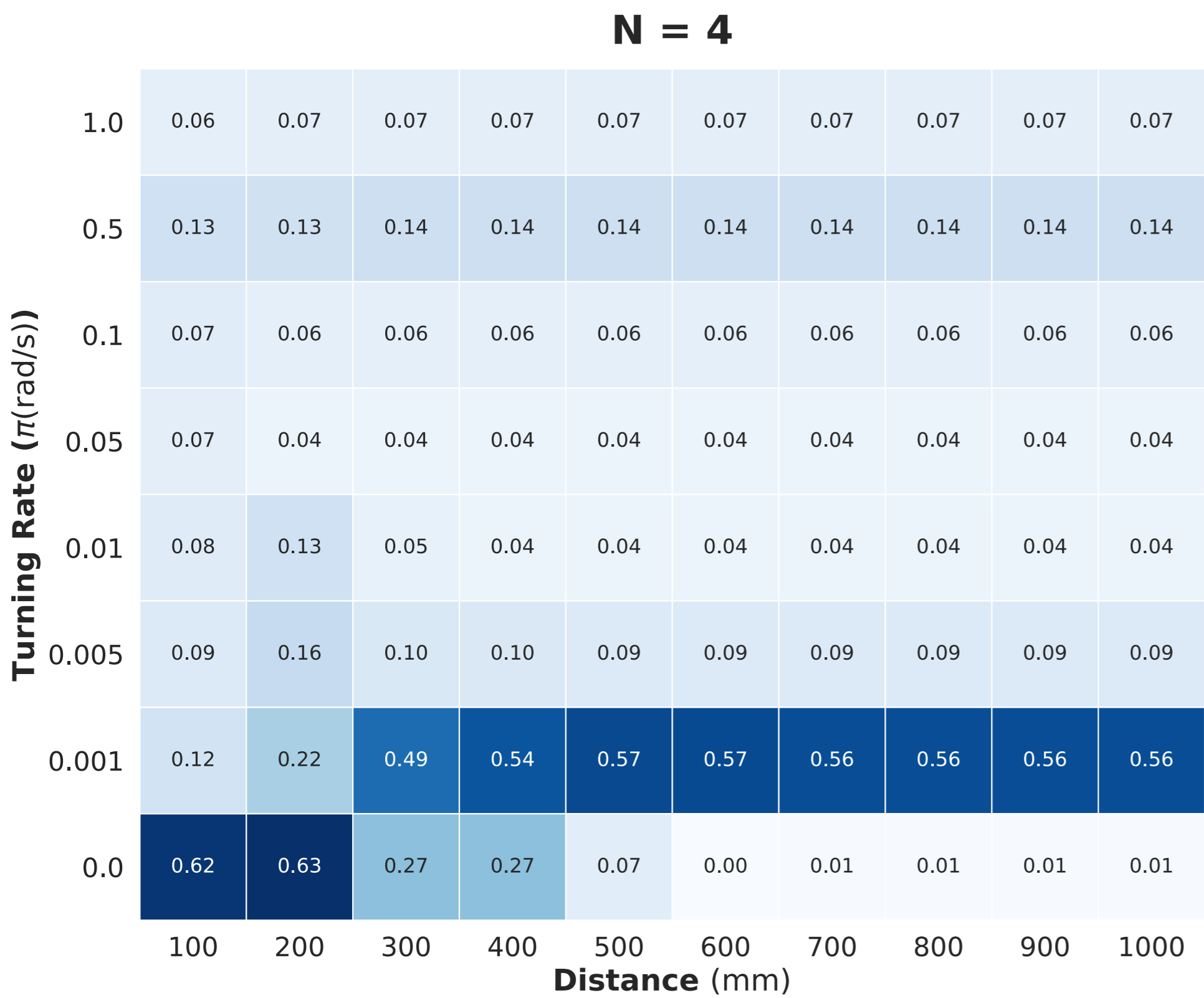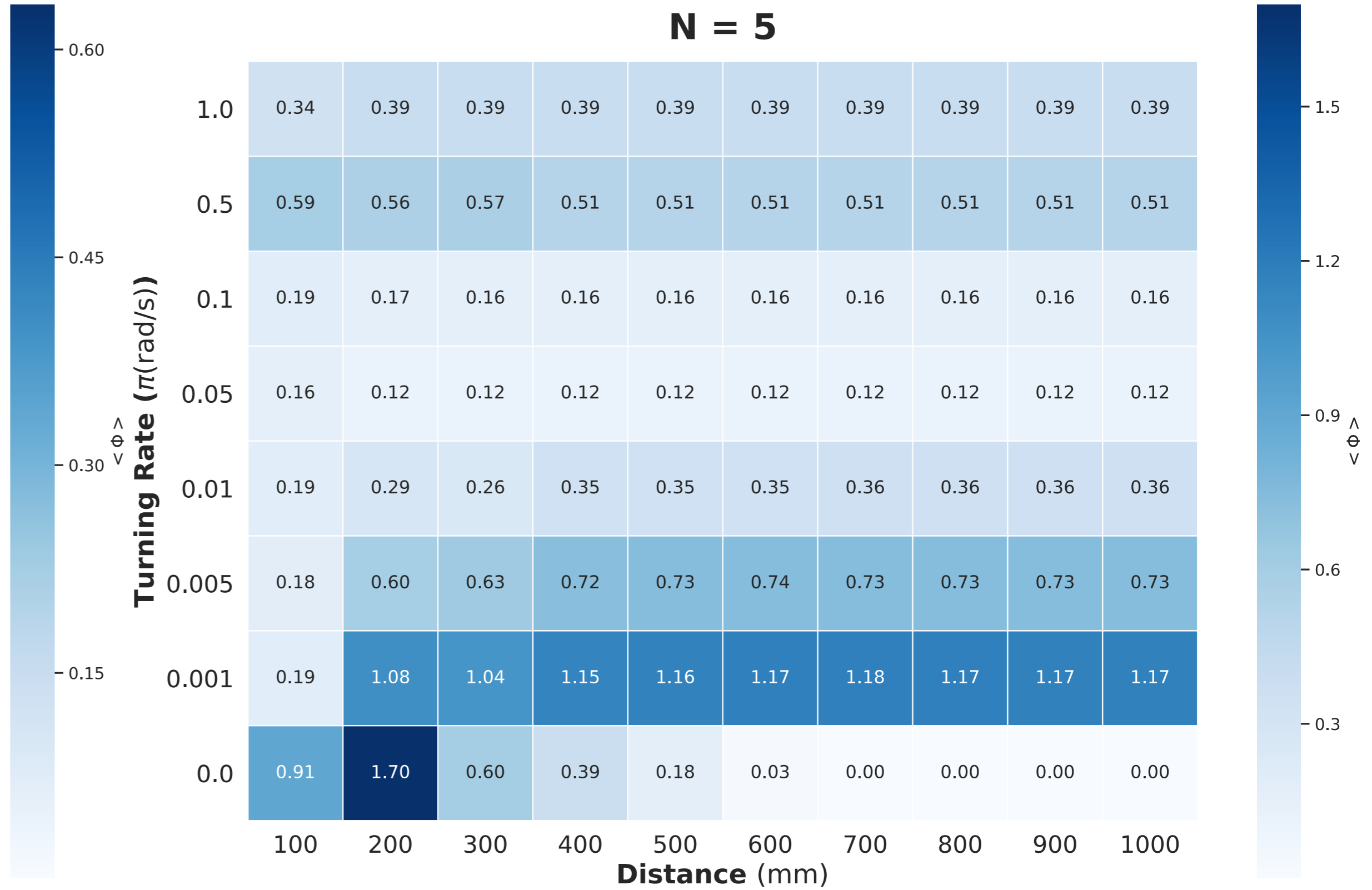

Degree - Turning Rate: fixed Dis = 1000 mm

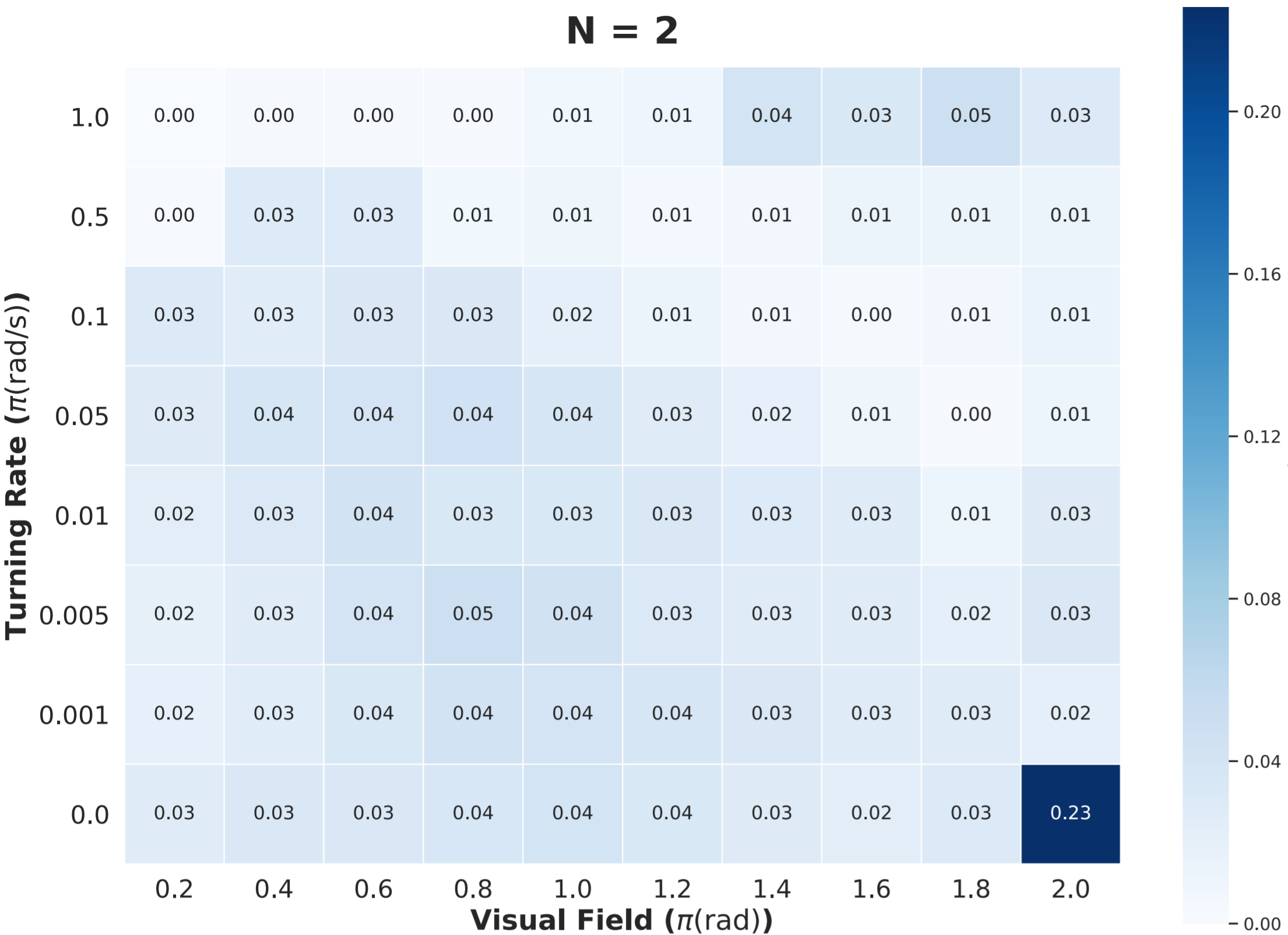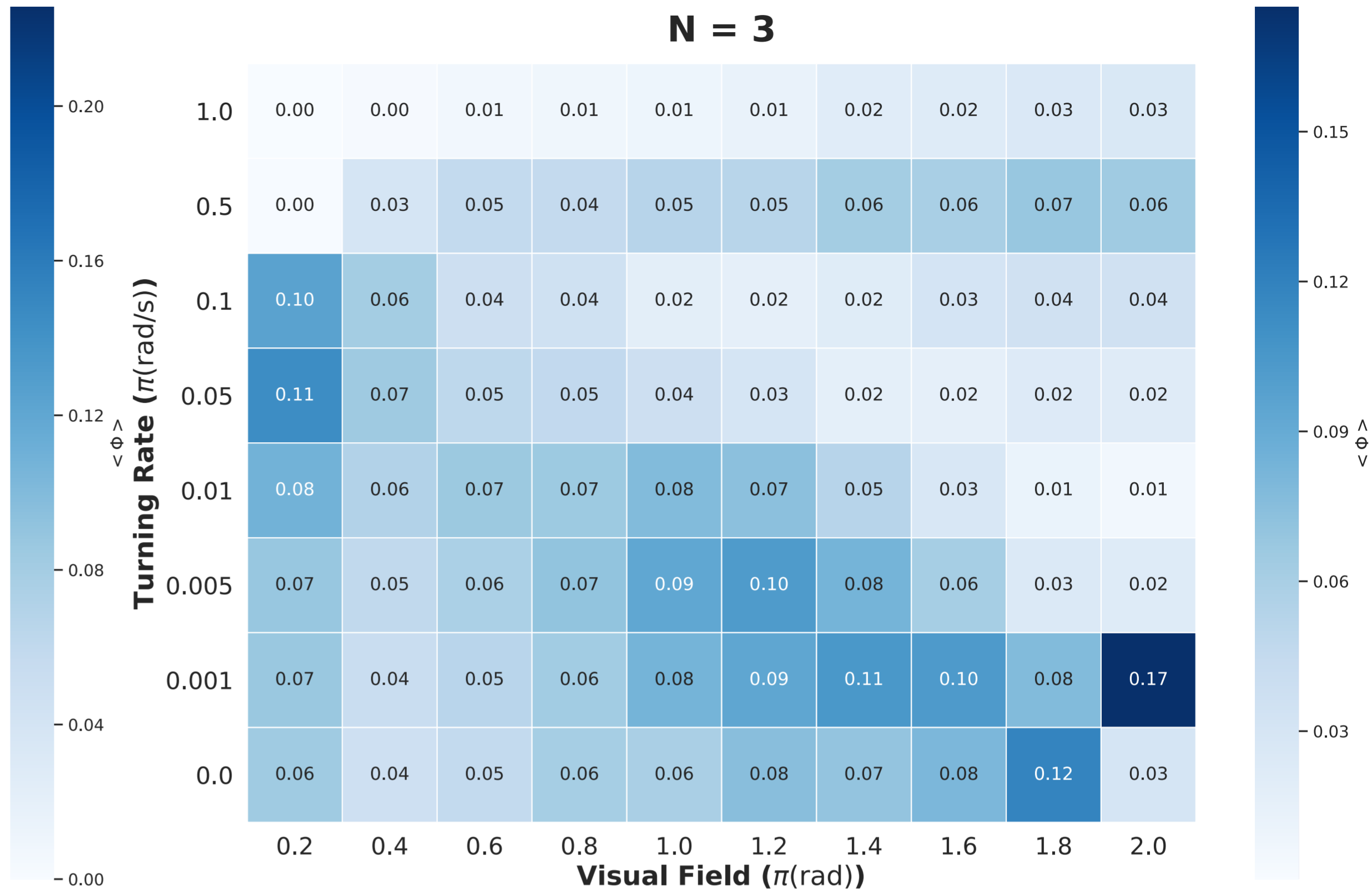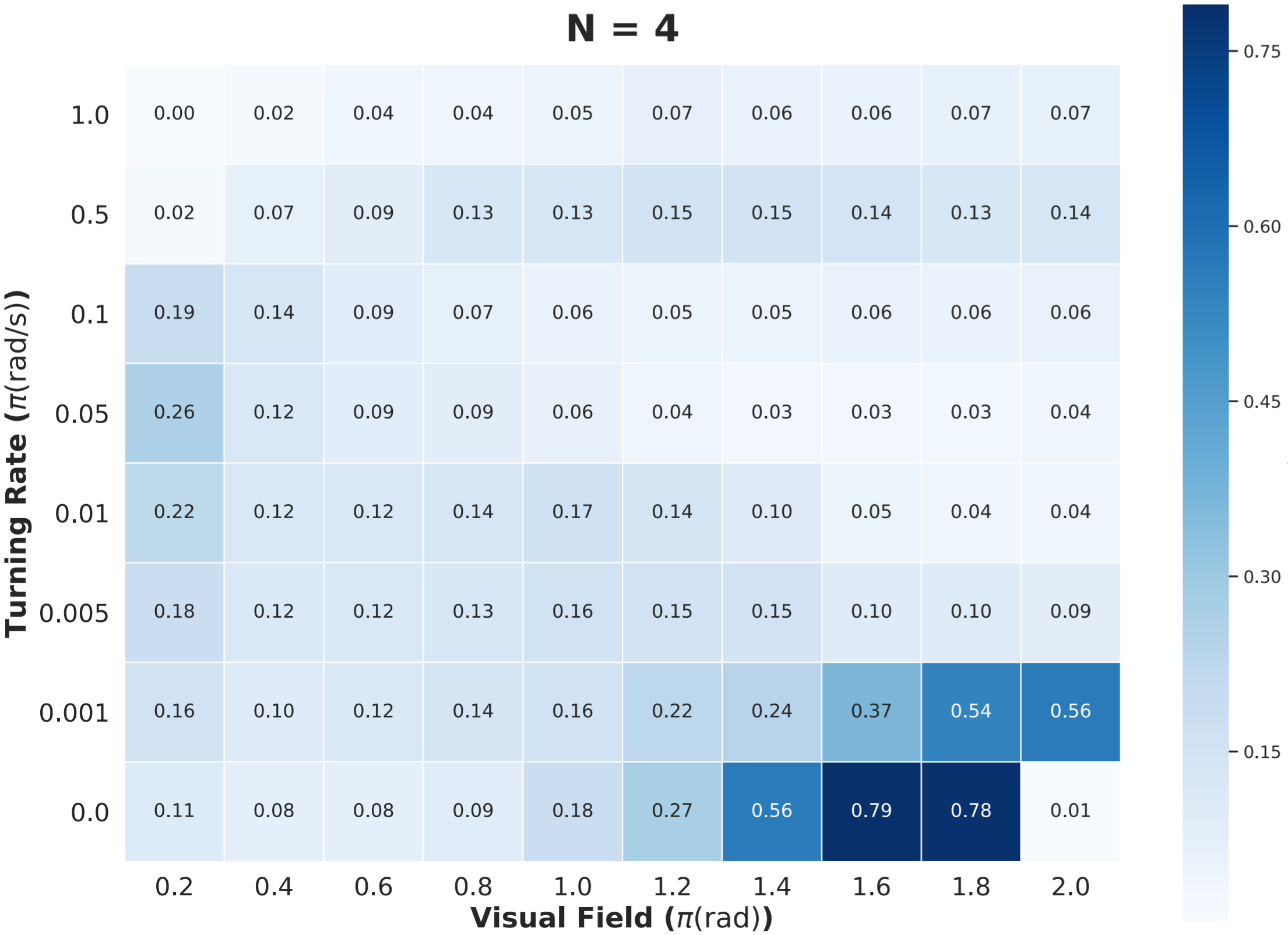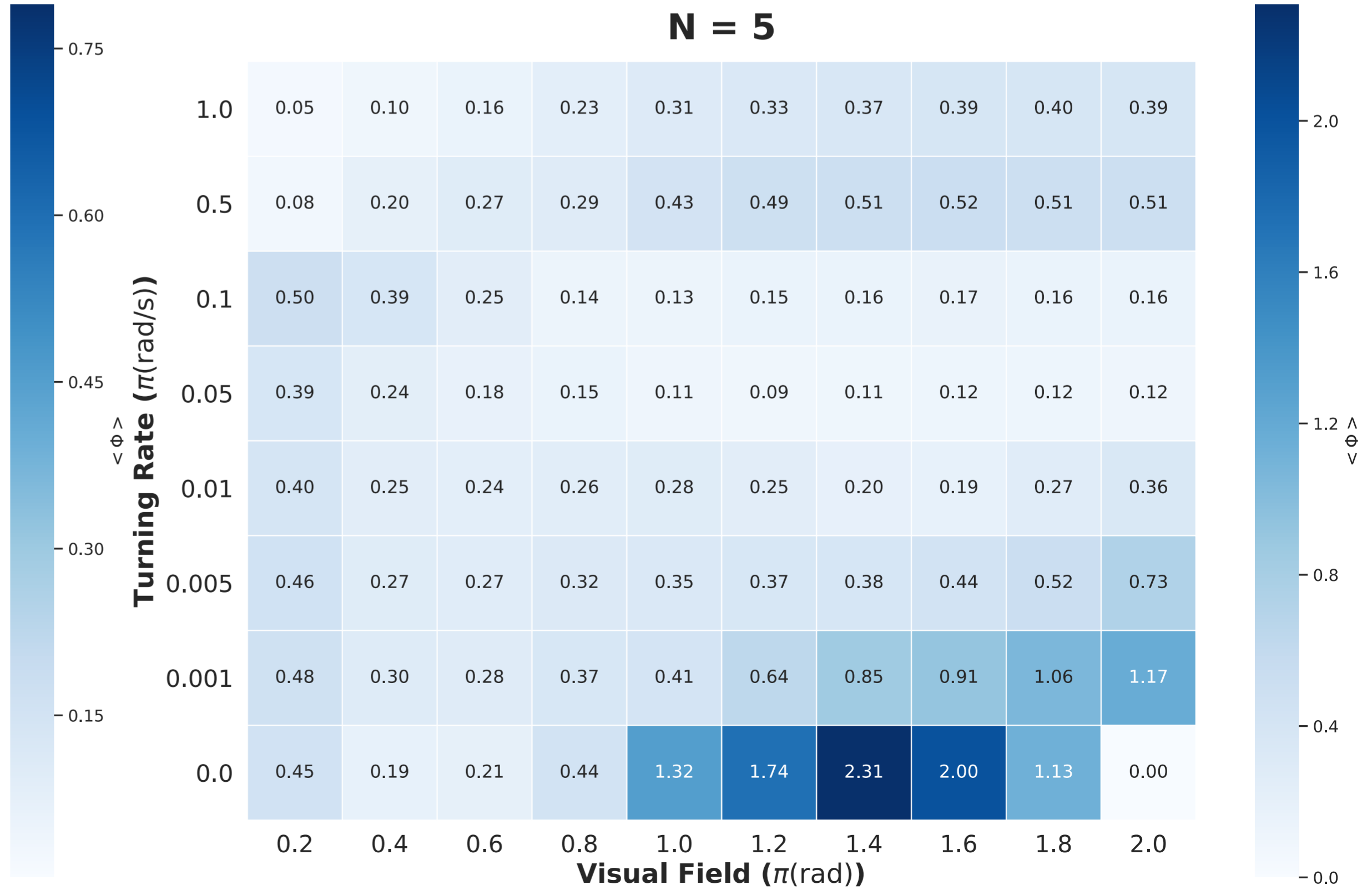

Supplement: S1 Fig — (PDF) [file pone.0229573.s001.pdf]

# Main Complex

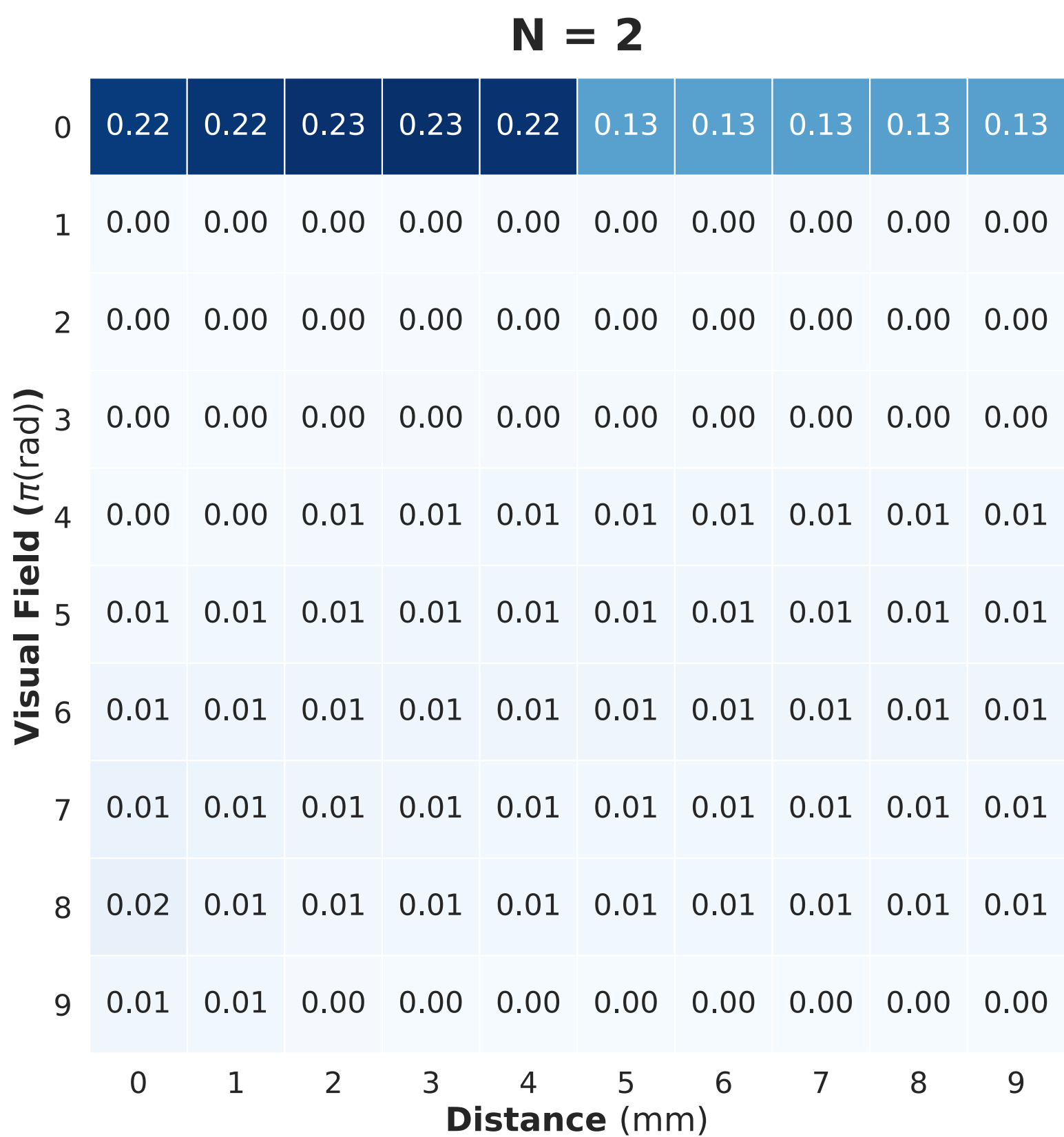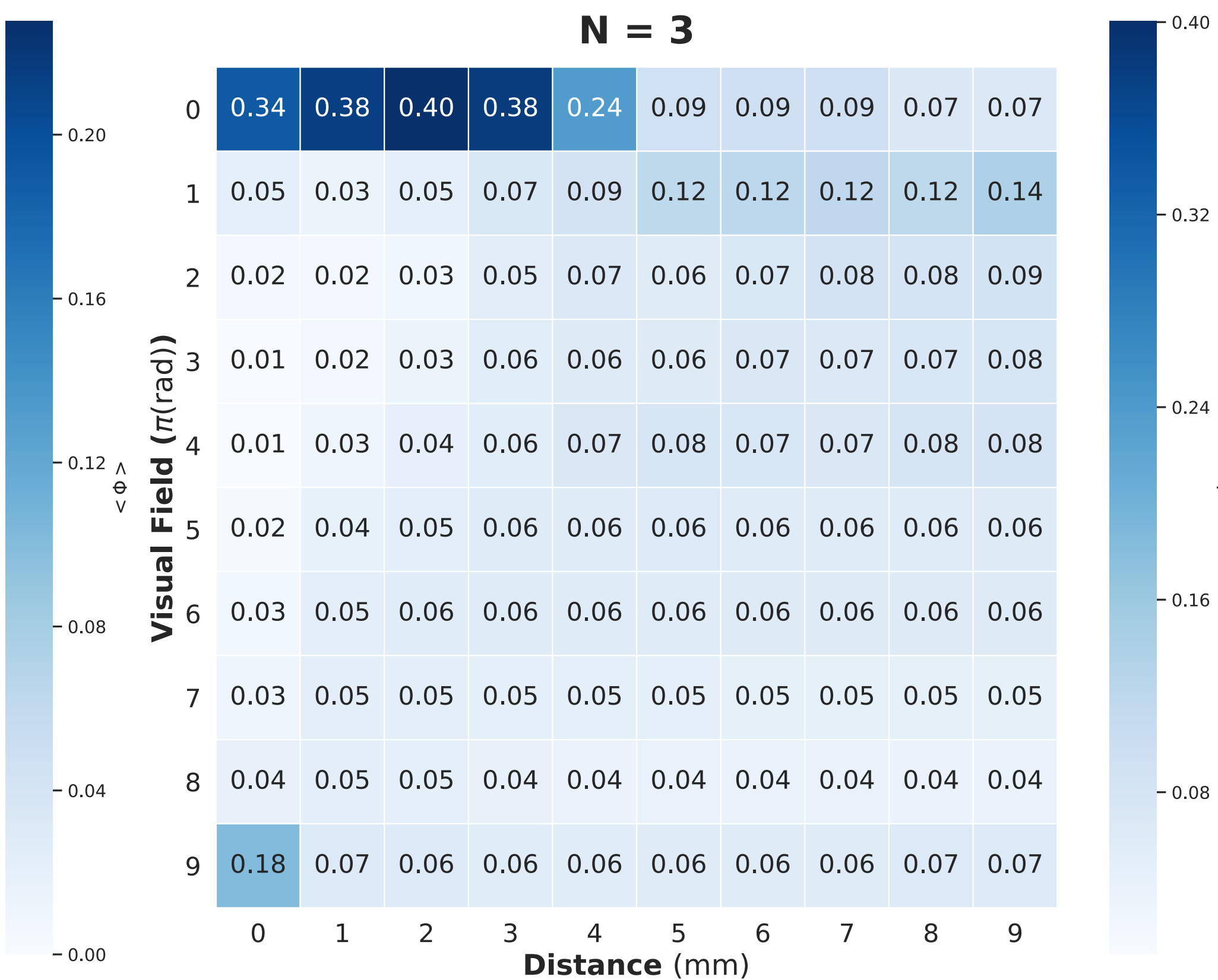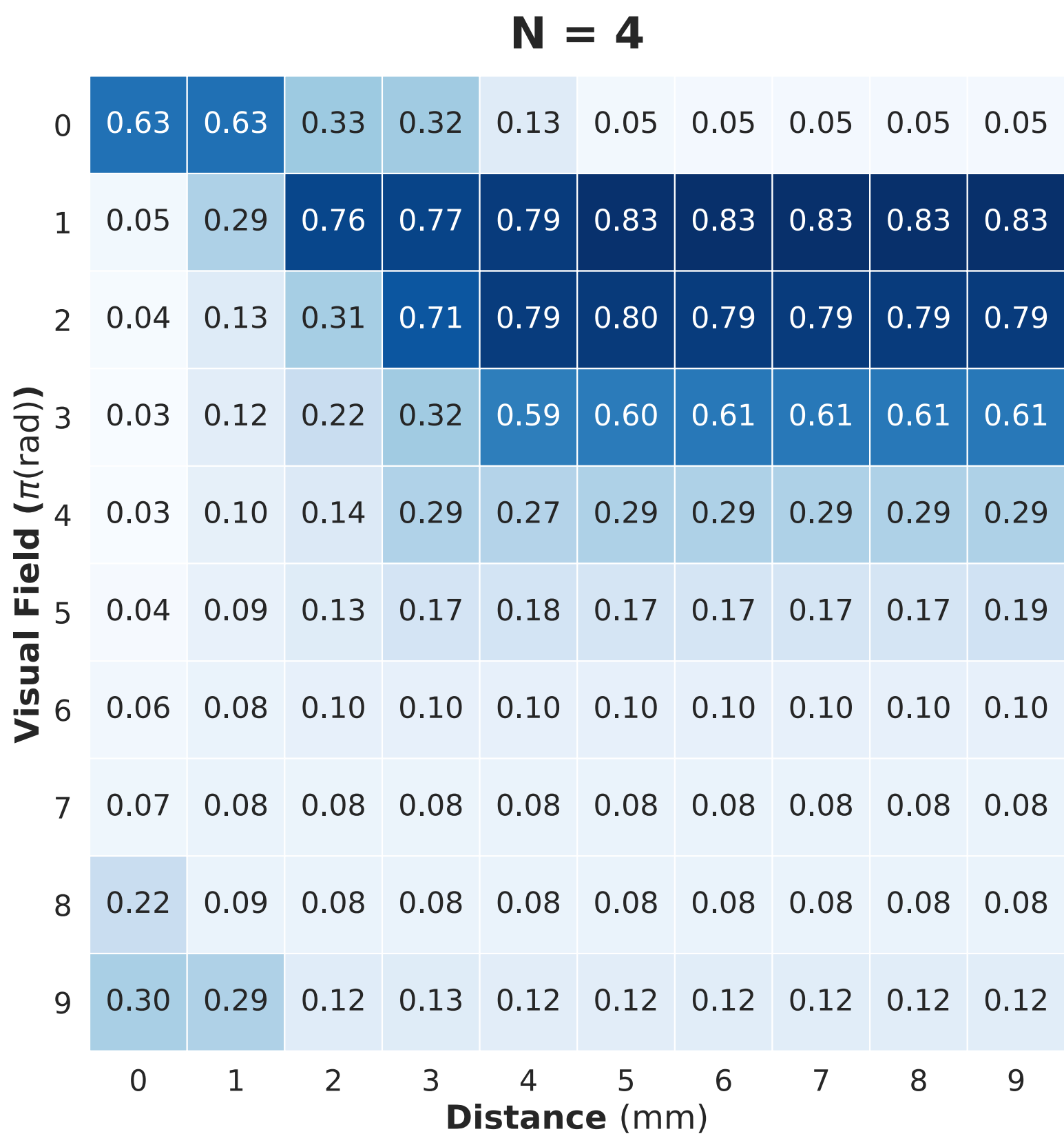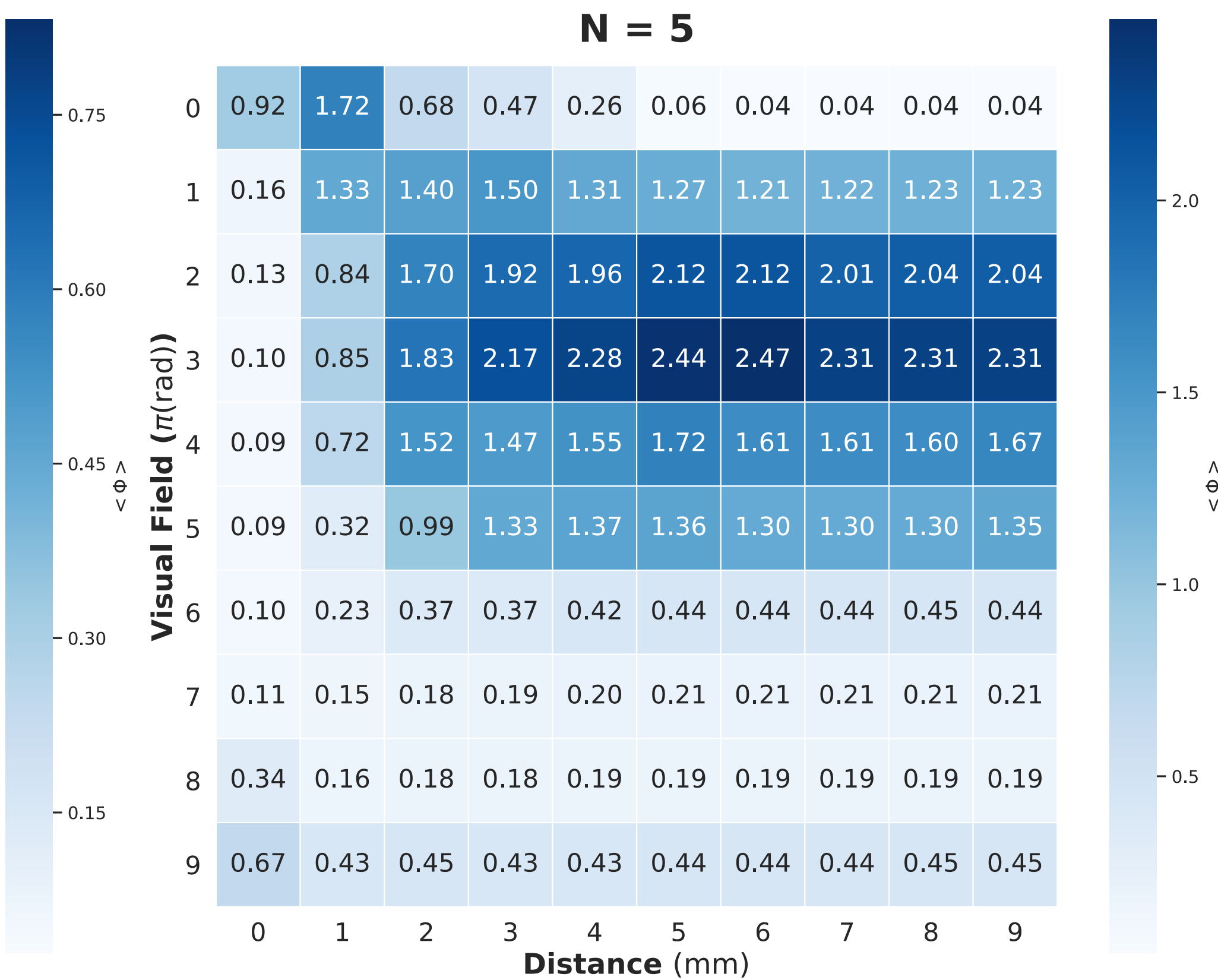

## Without Self-Loop

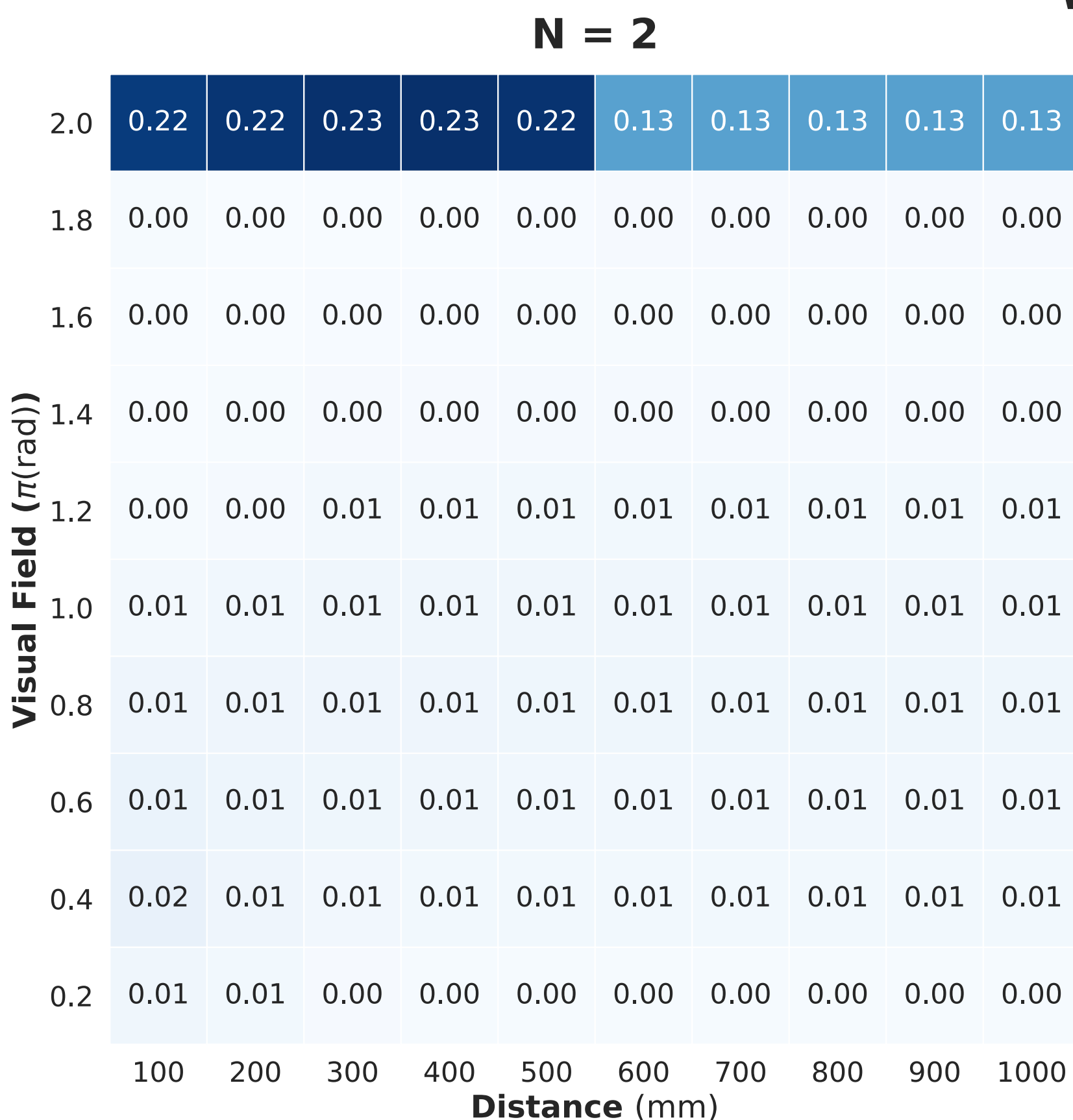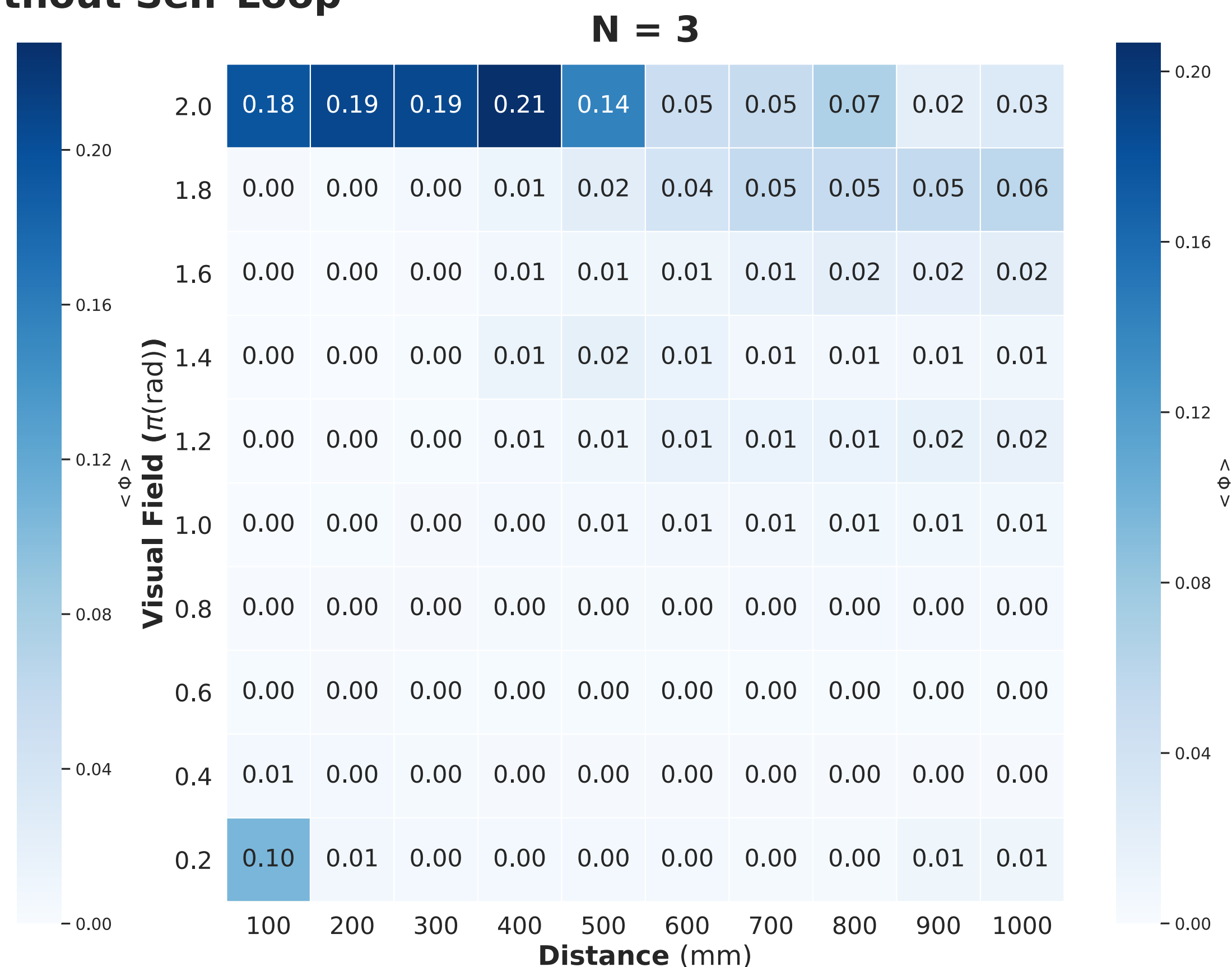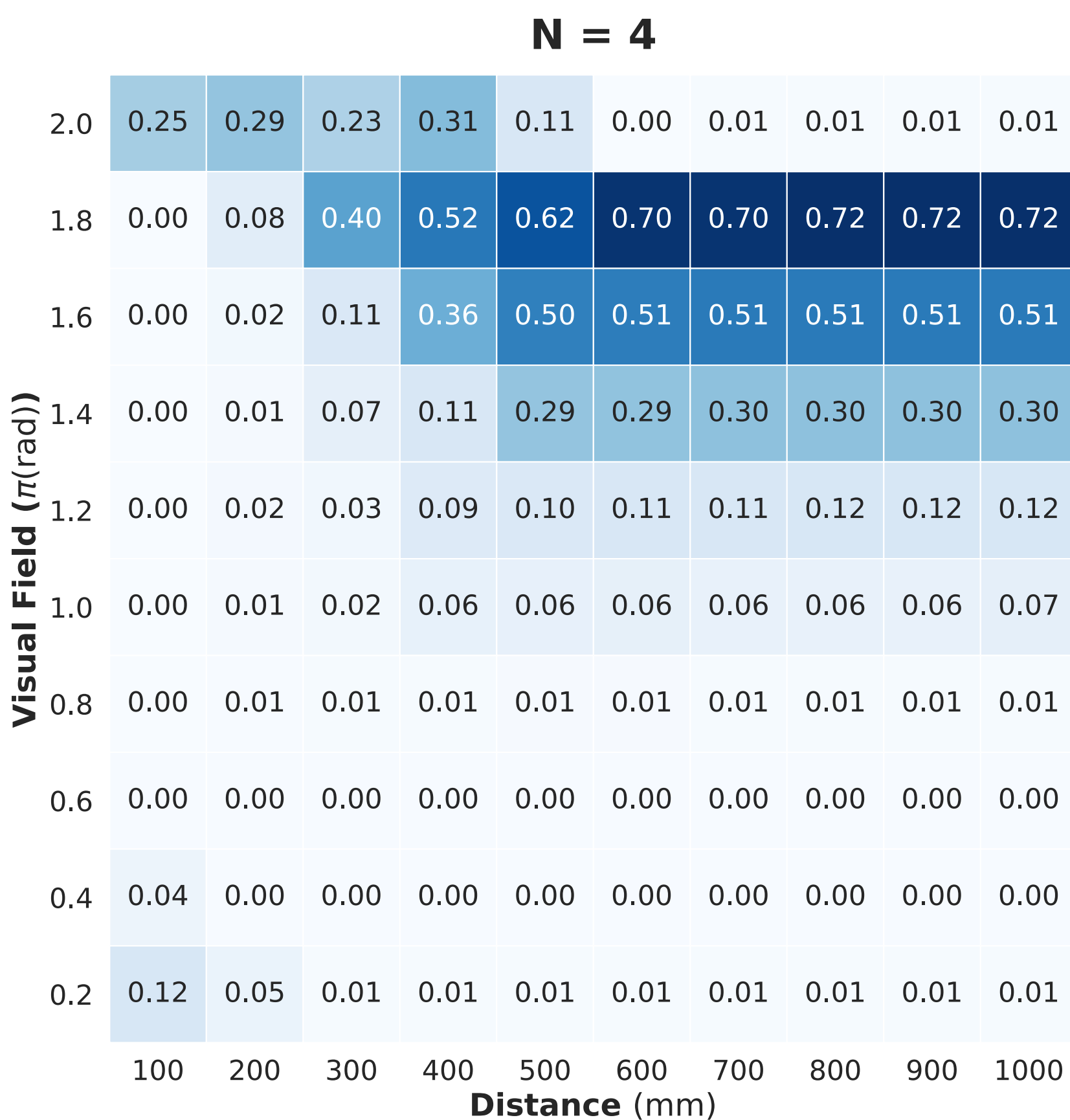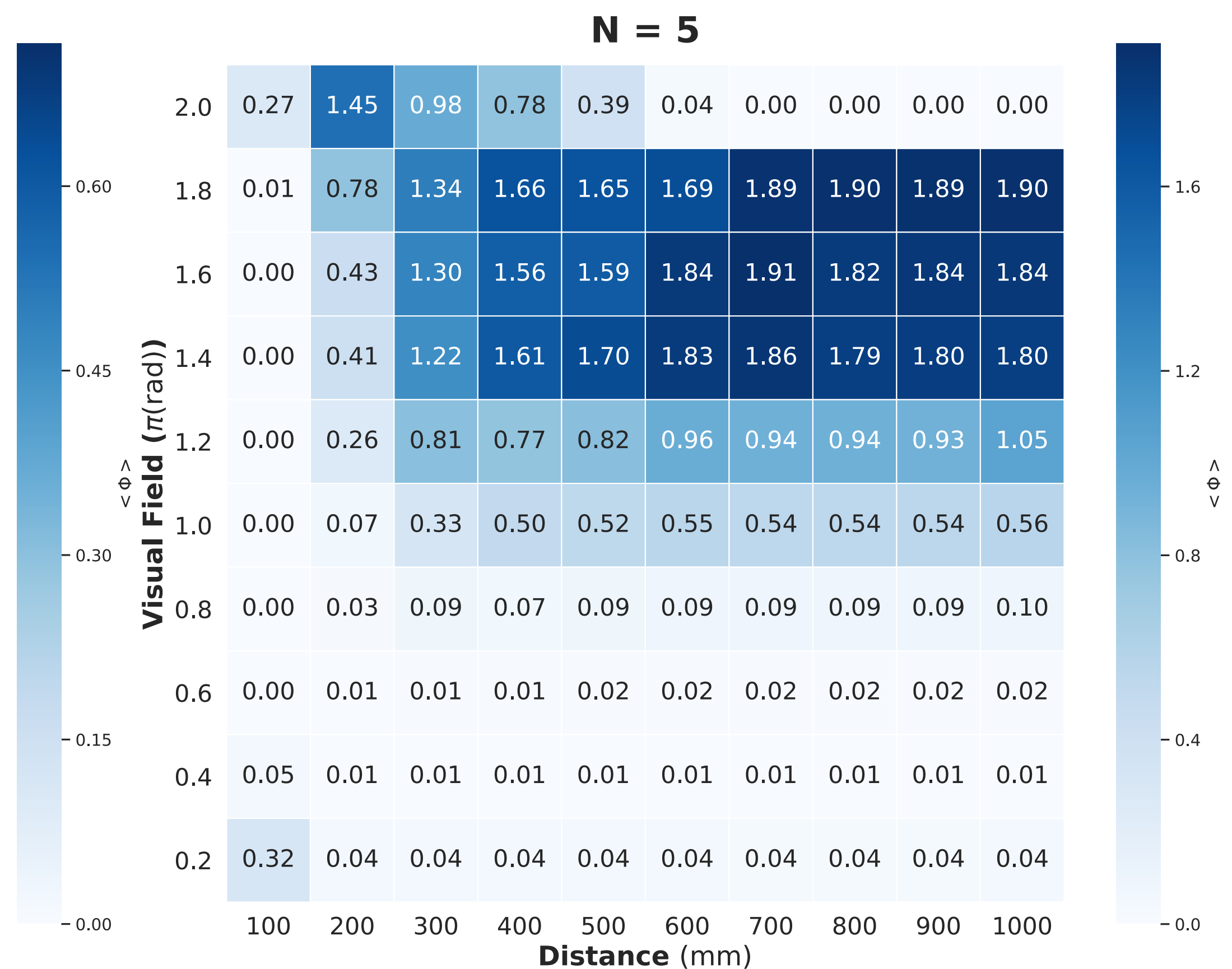

Supplement: S4 Fig — (PDF) [file pone.0229573.s004.pdf]

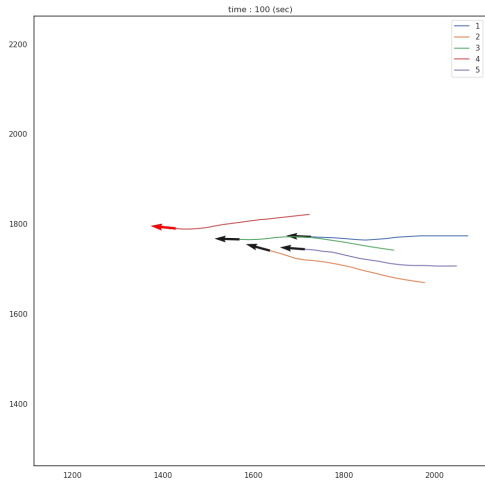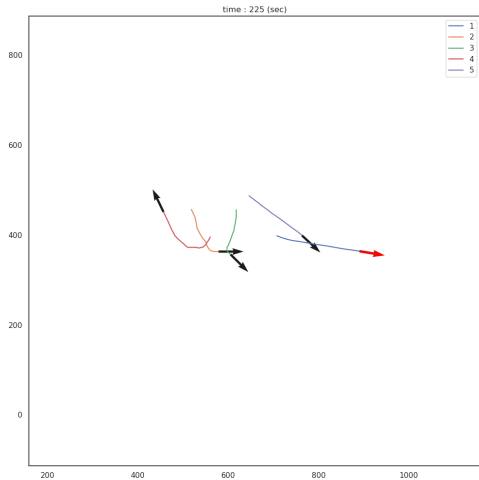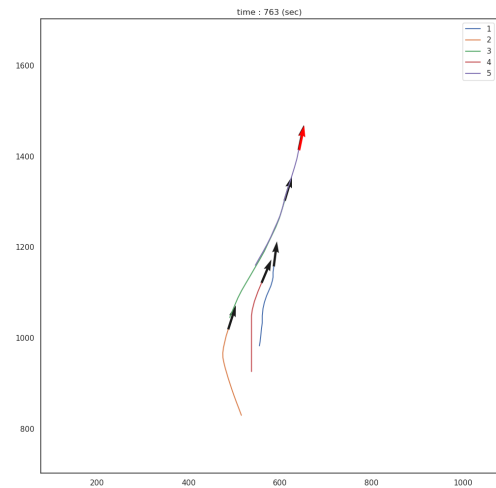

Supplement: S5 Fig — The head of the group corresponds to the IIT leader. (PDF) [file pone.0229573.s005.pdf]

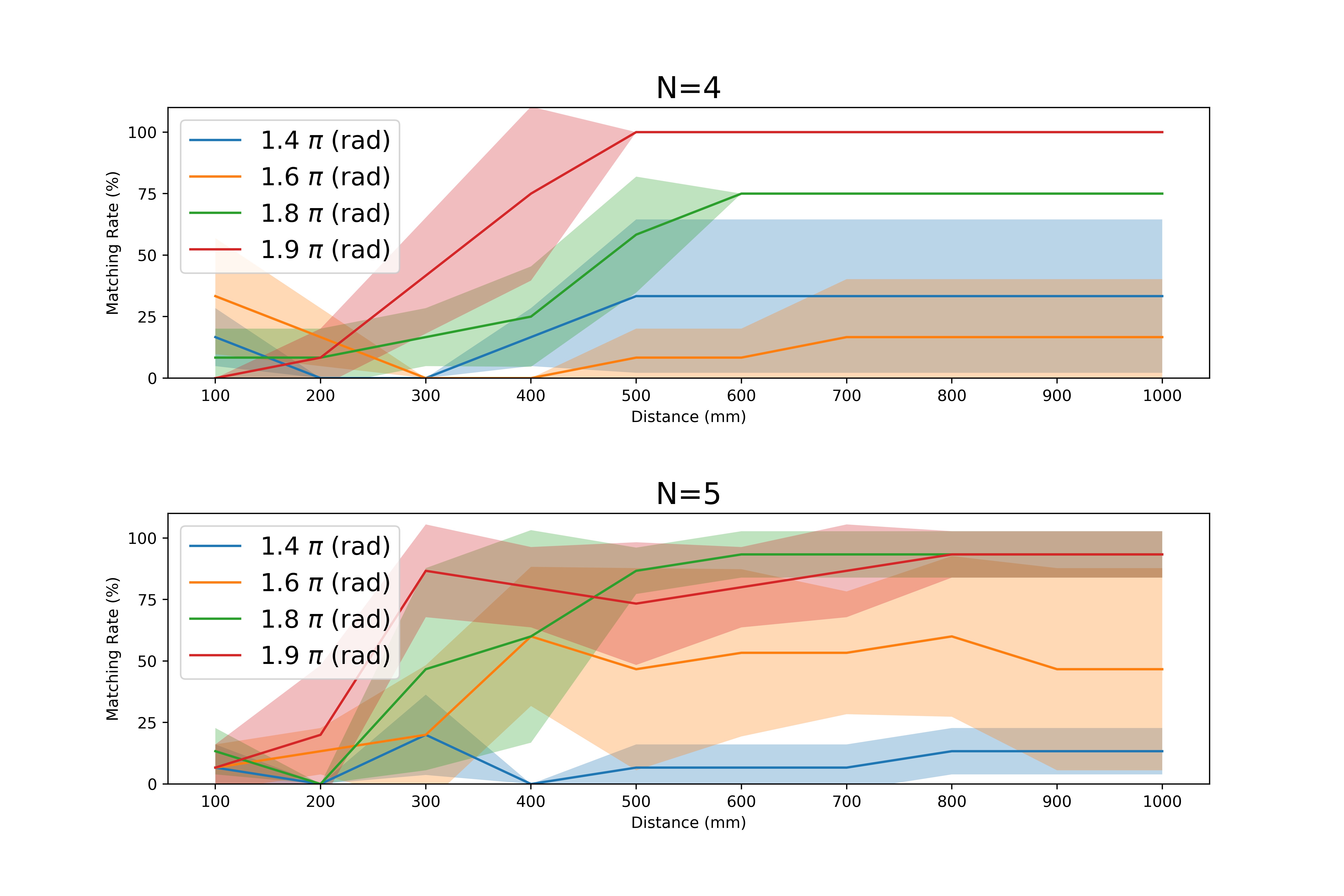

Supplement: S6 Fig — (JPG) [file pone.0229573.s006.jpg]

# Boid Fish Trajectory

$N = 2$

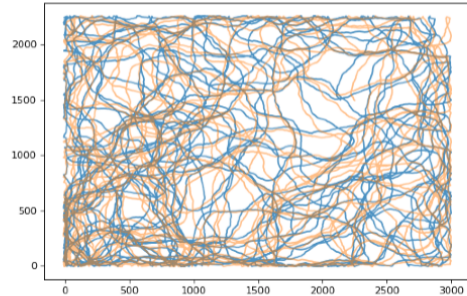

$N = 3$

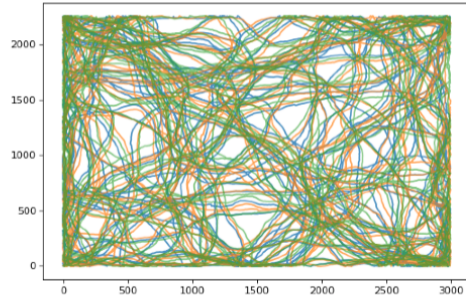

$N = 4$

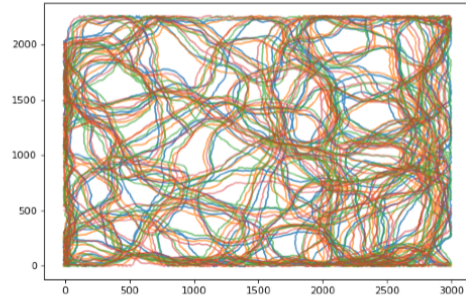

$N = 5$

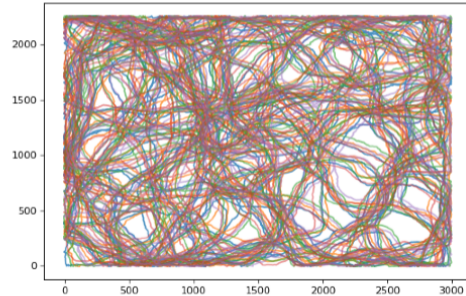

# Real Fish Trajectory

$N = 2$

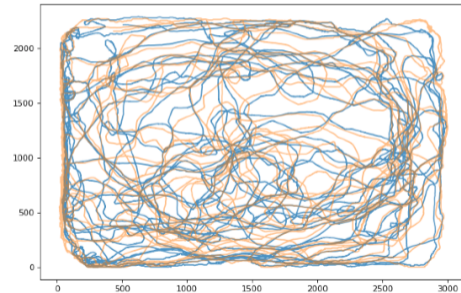

$N = 3$

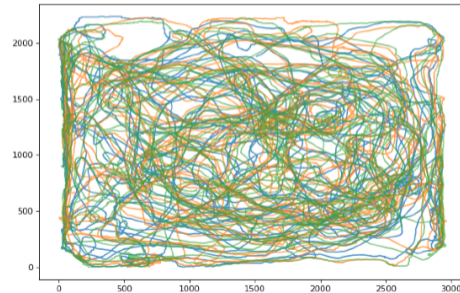

$N = 4$

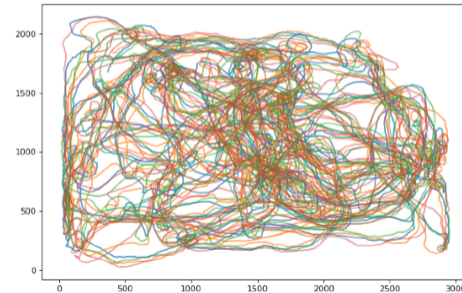

$N = 5$

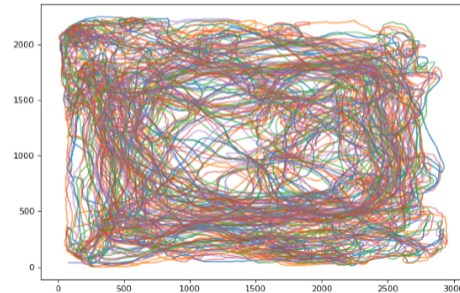

Supplement: S8 Fig — (PDF) [file pone.0229573.s008.pdf]

## Mean mutual information for Boids with $C=1.0$

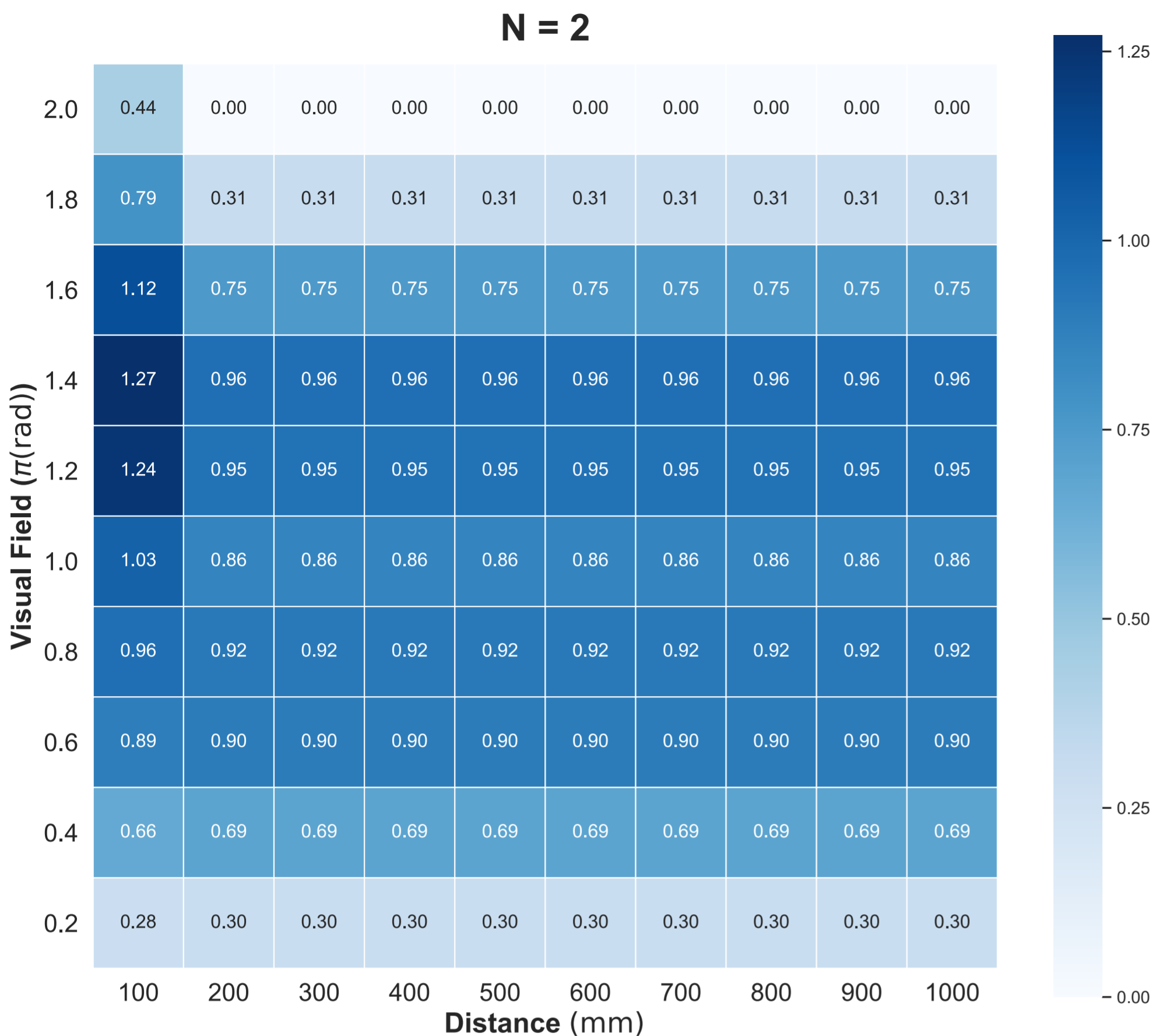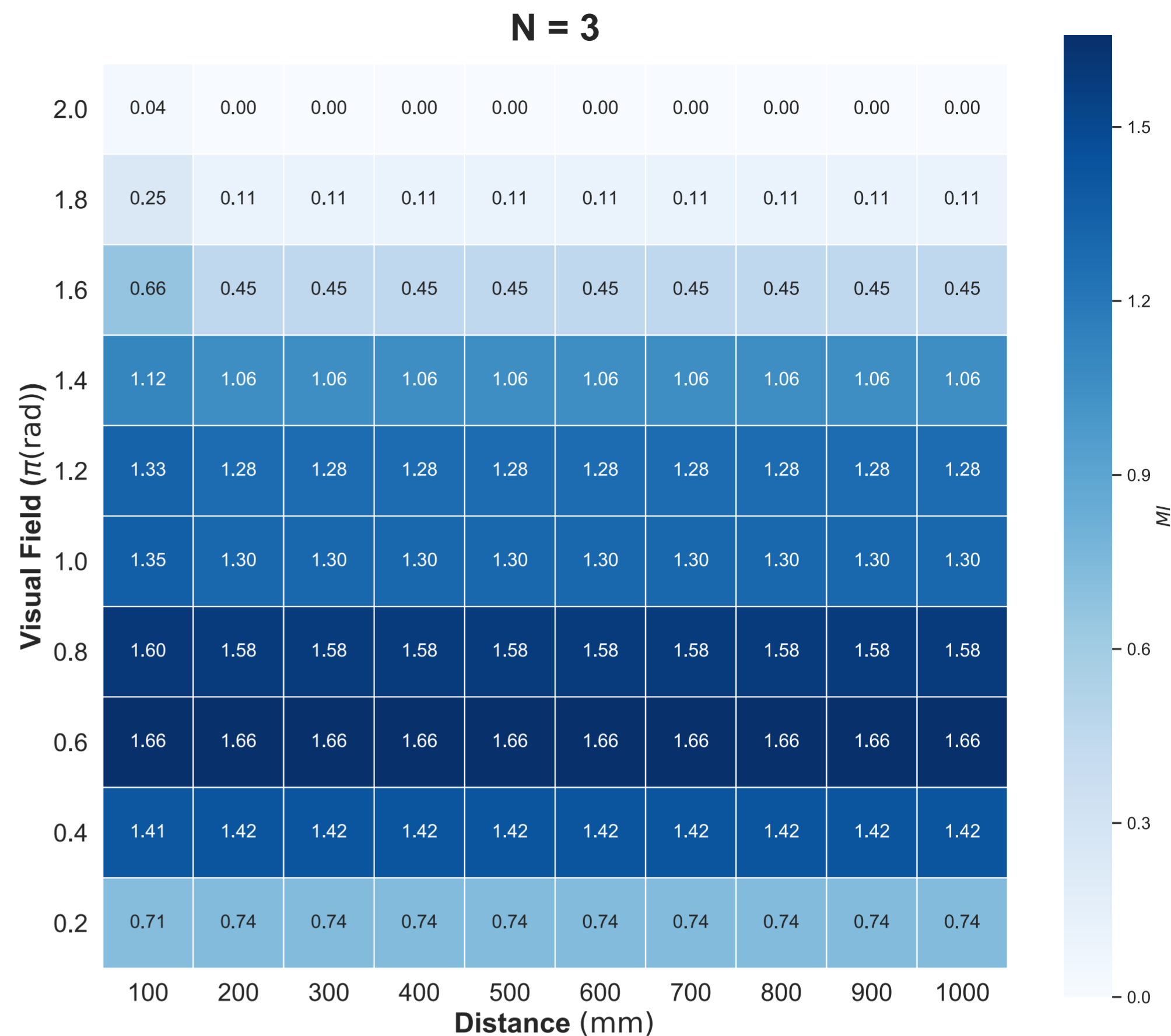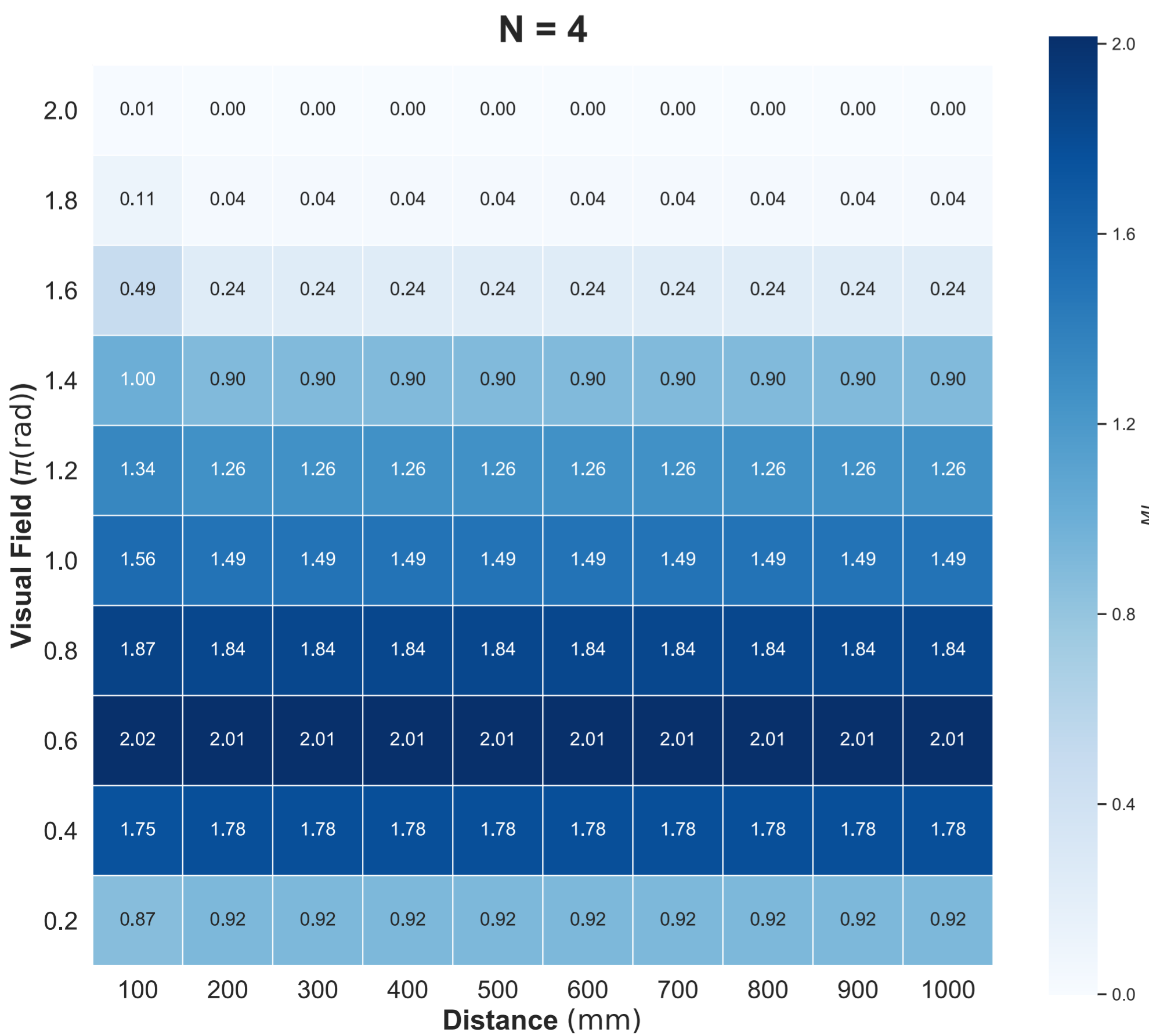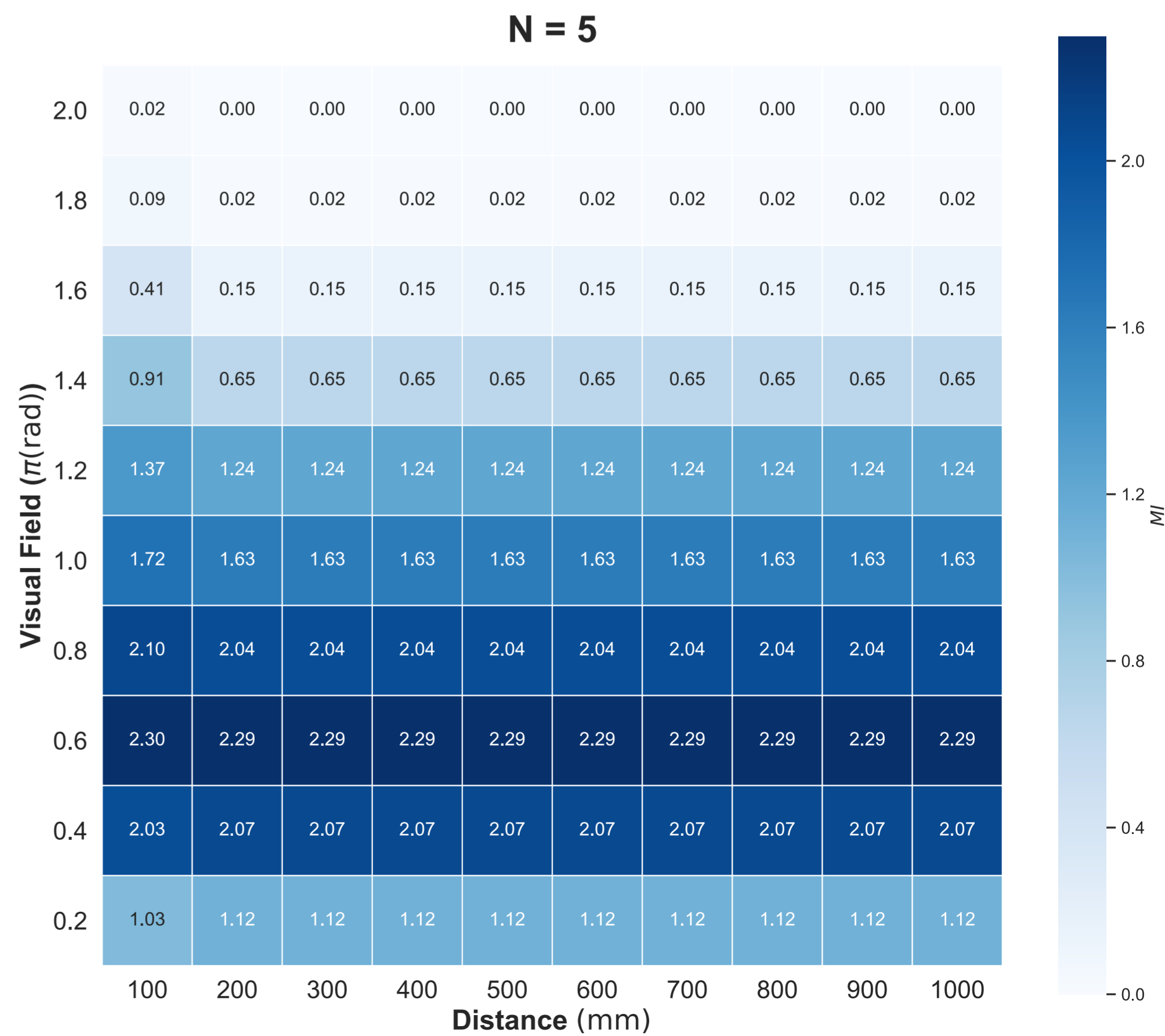

## Mean transfer entropy for Boids with C=1.0

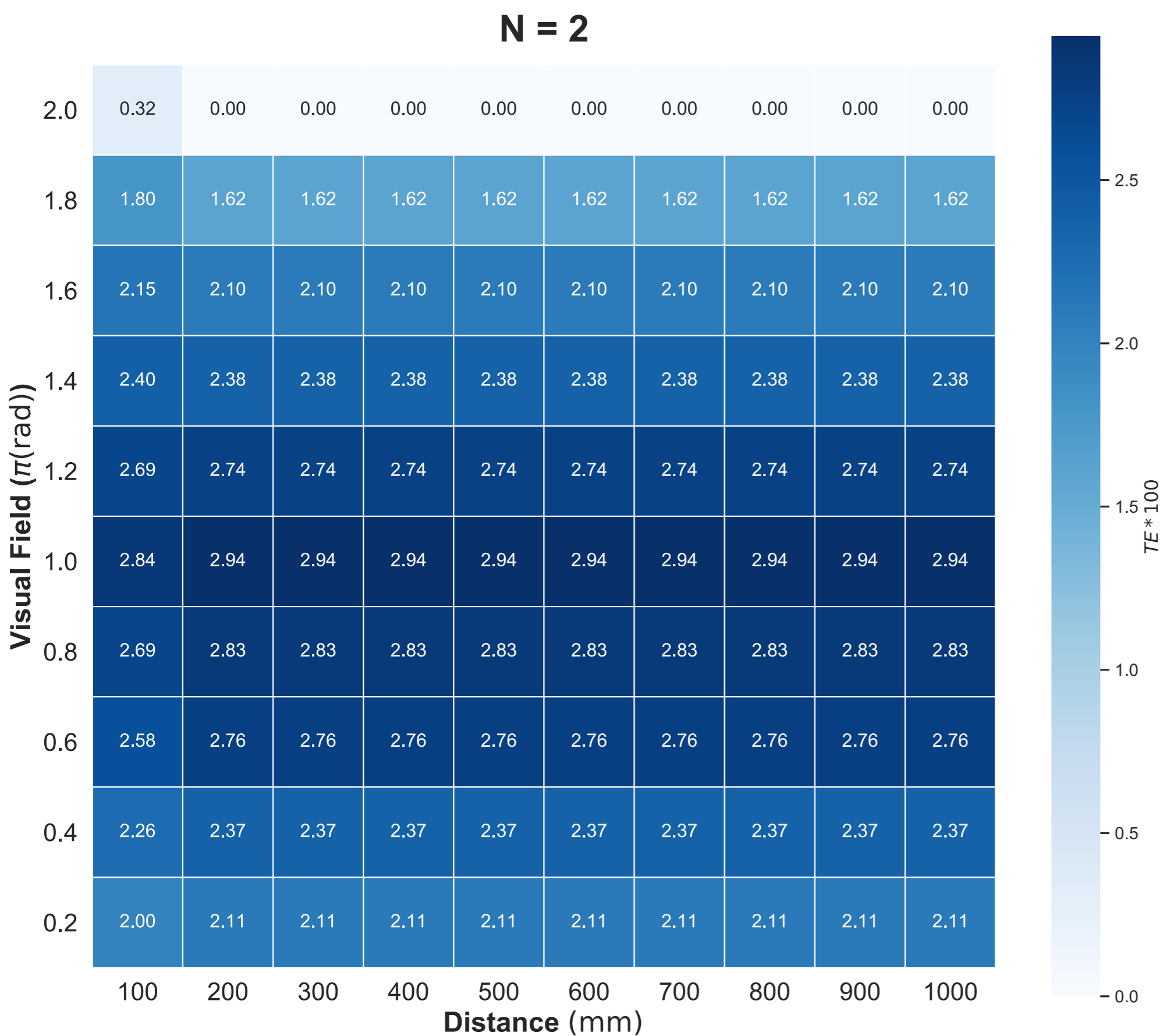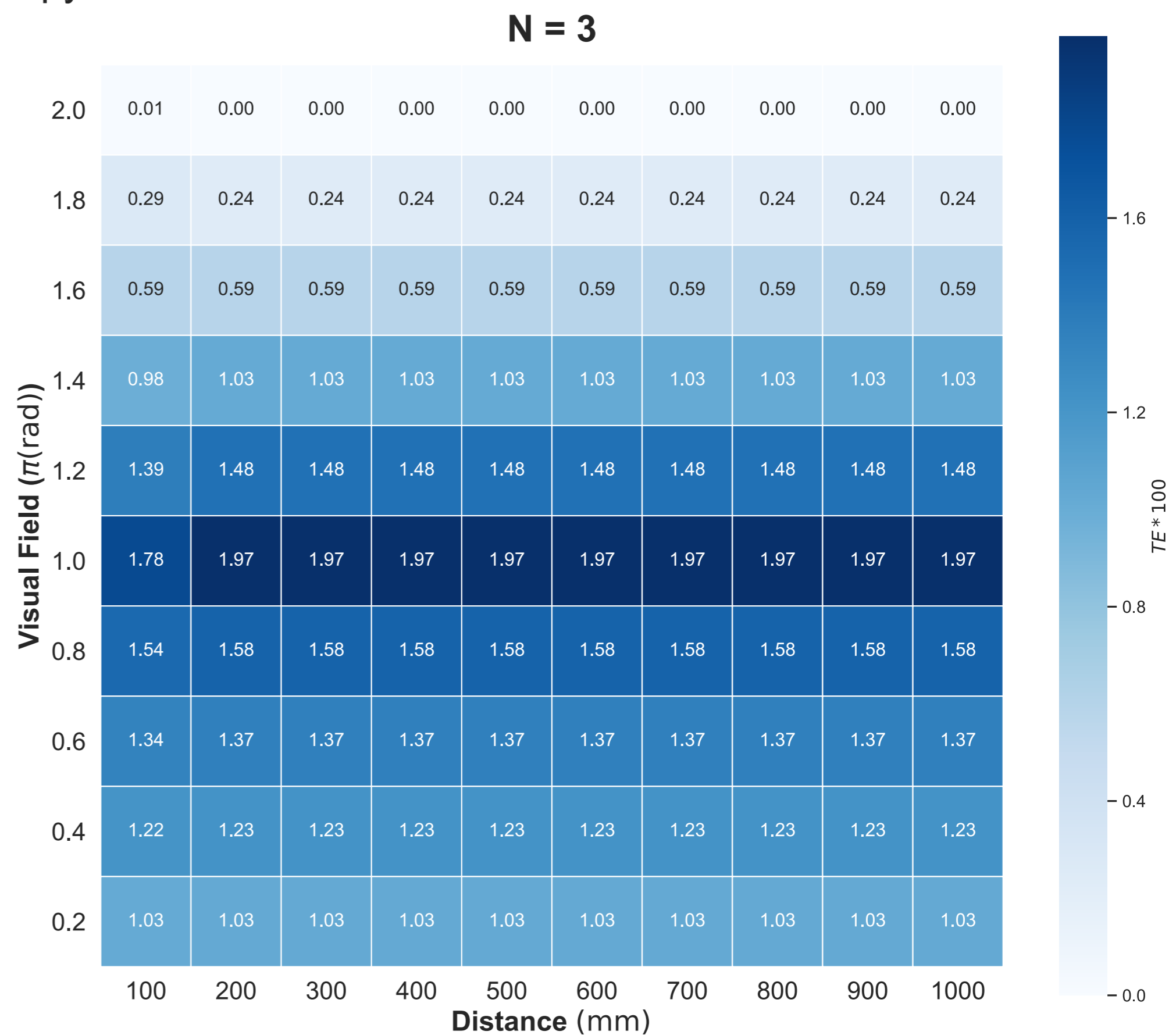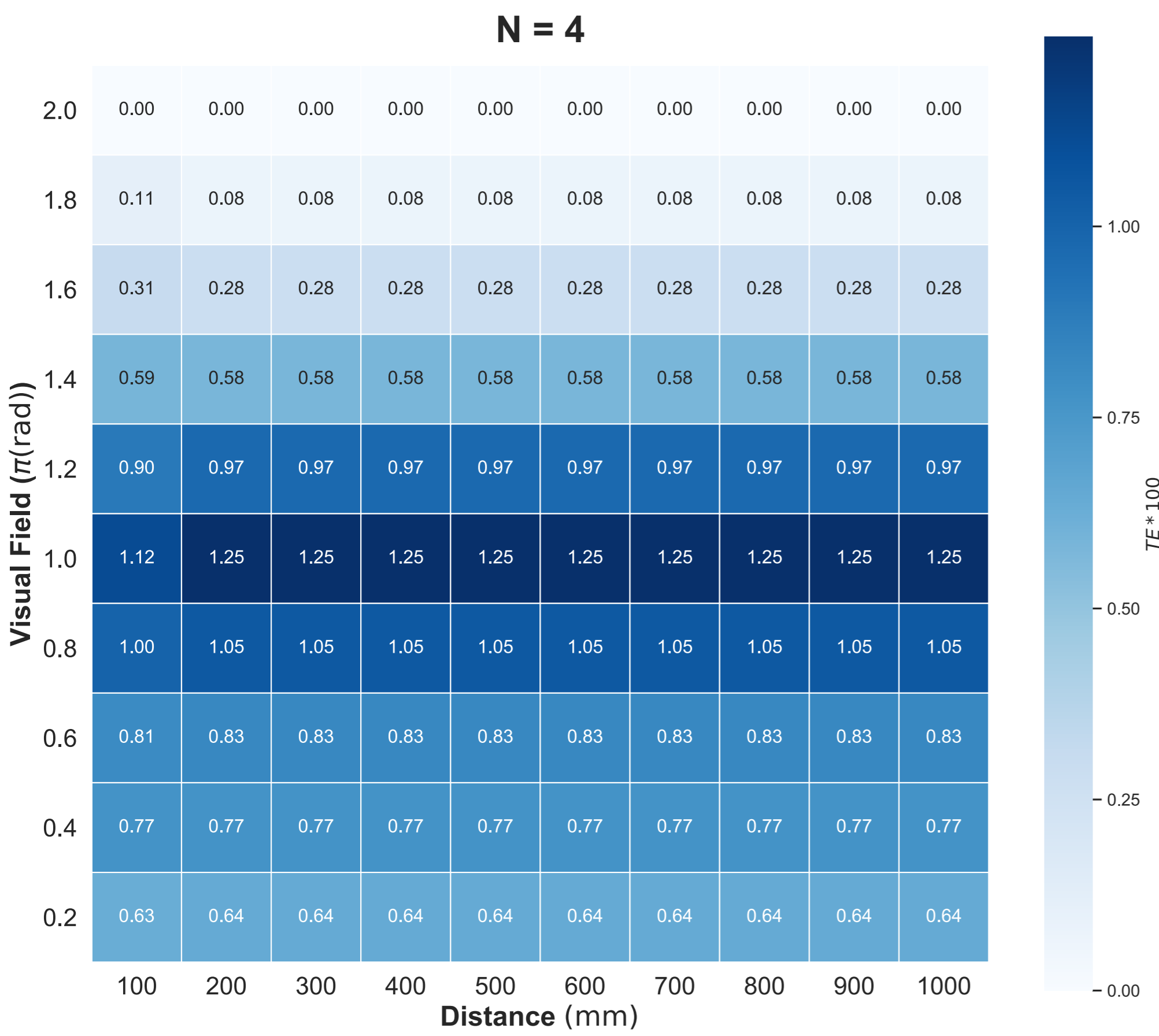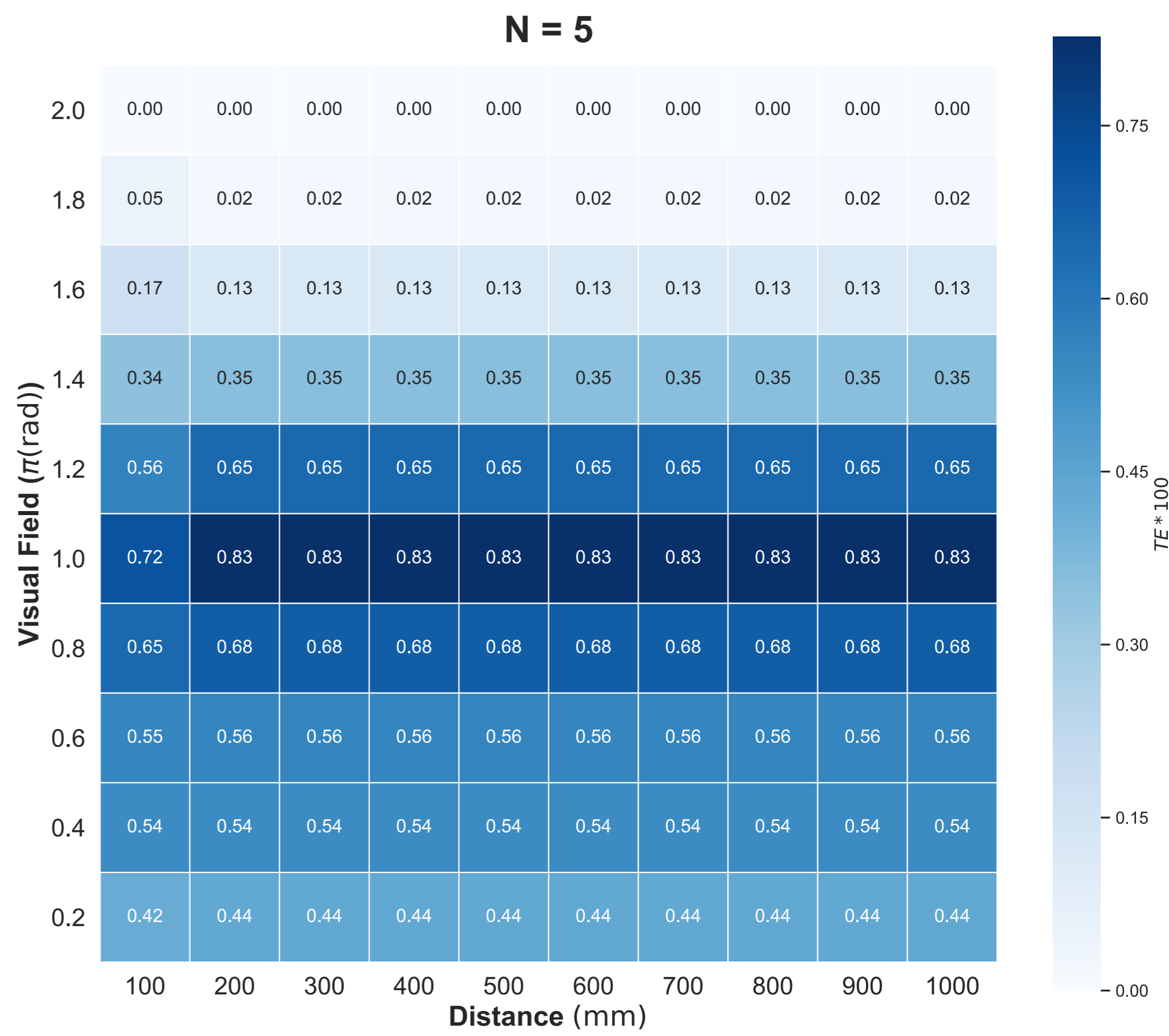

Supplement: S9 Fig — (PDF) [file pone.0229573.s009.pdf]

$$C = 0$$

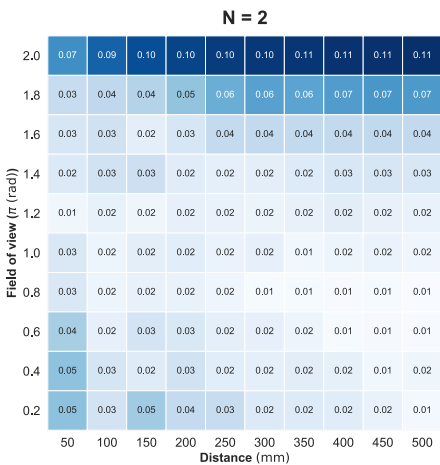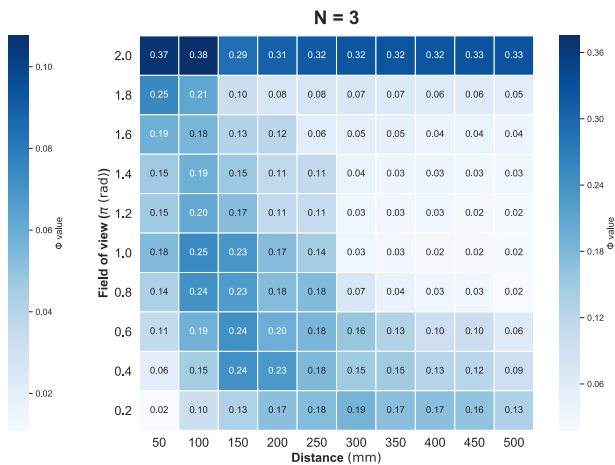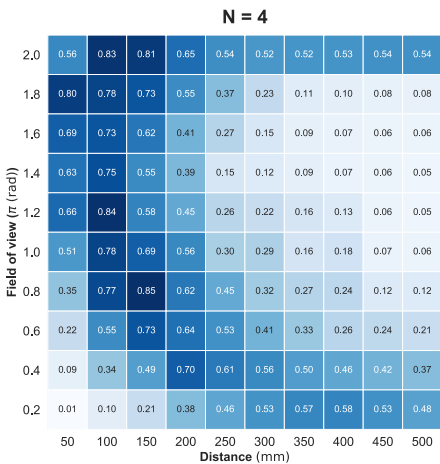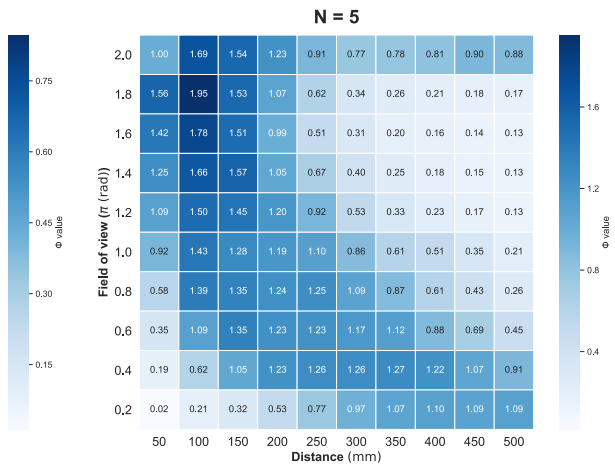

**C = 0.01**

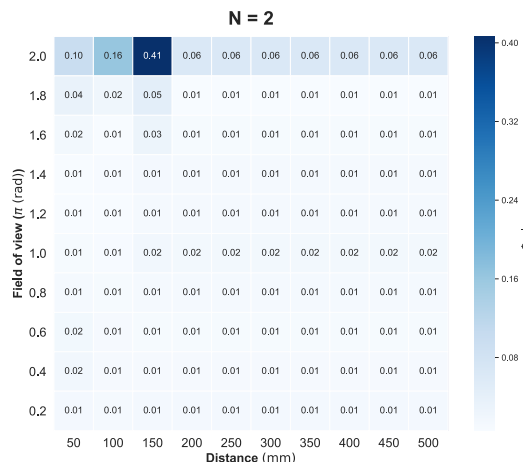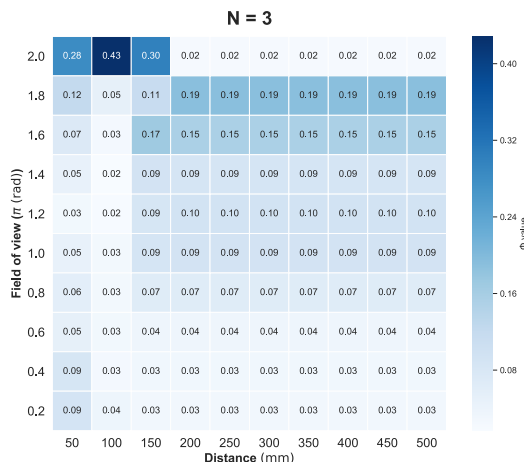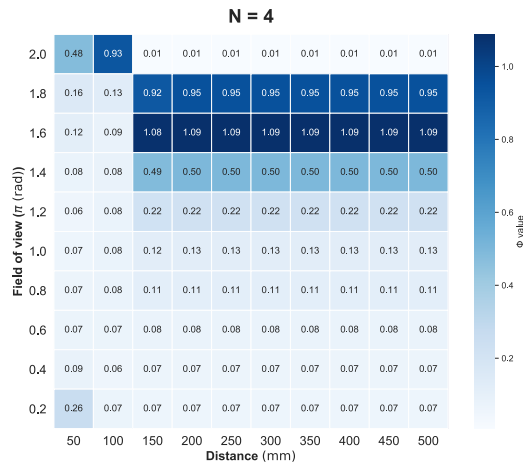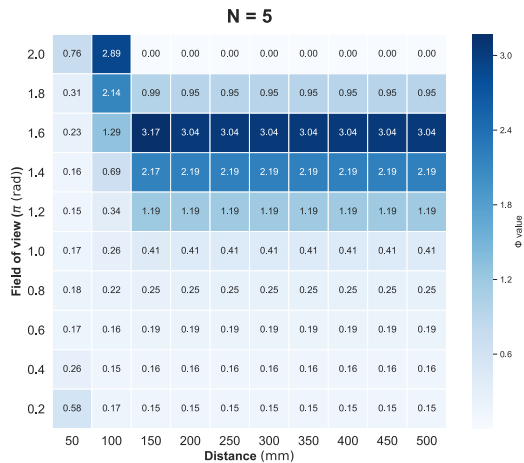

$$C = 0.1$$

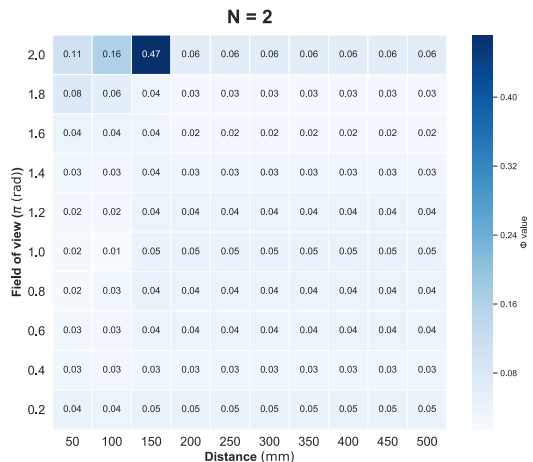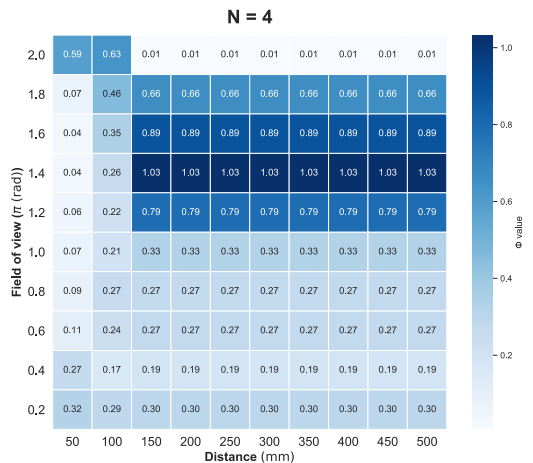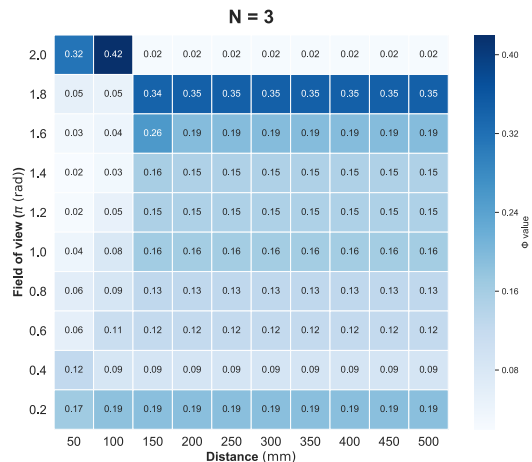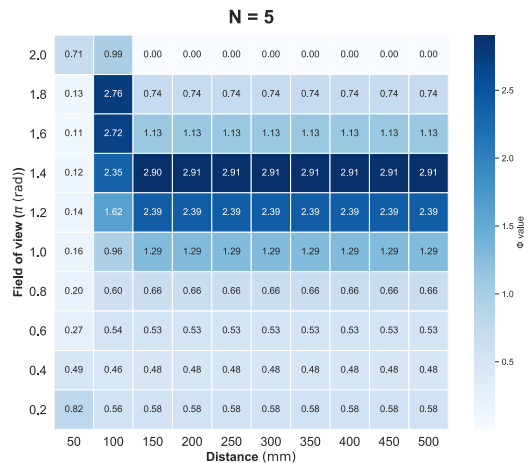

# C = 1.0

## N = 2

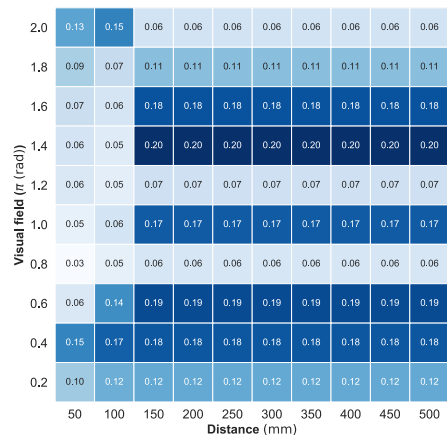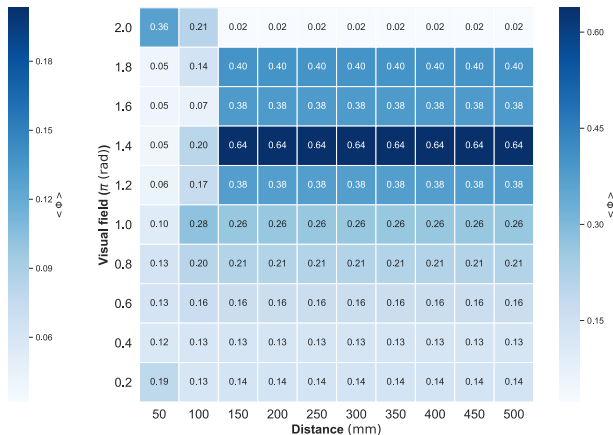

## N = 4

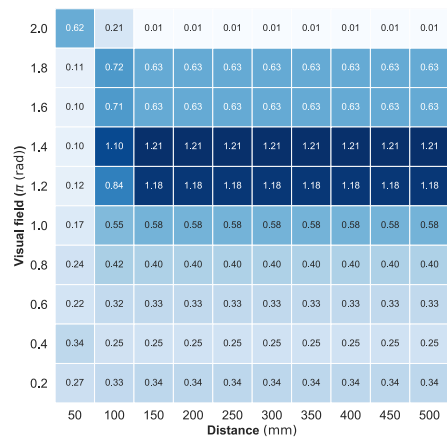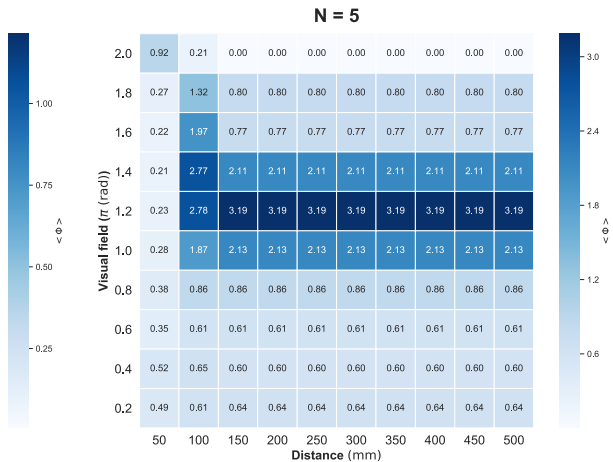

Supplement: S10 Fig — (PDF) [file pone.0229573.s010.pdf]

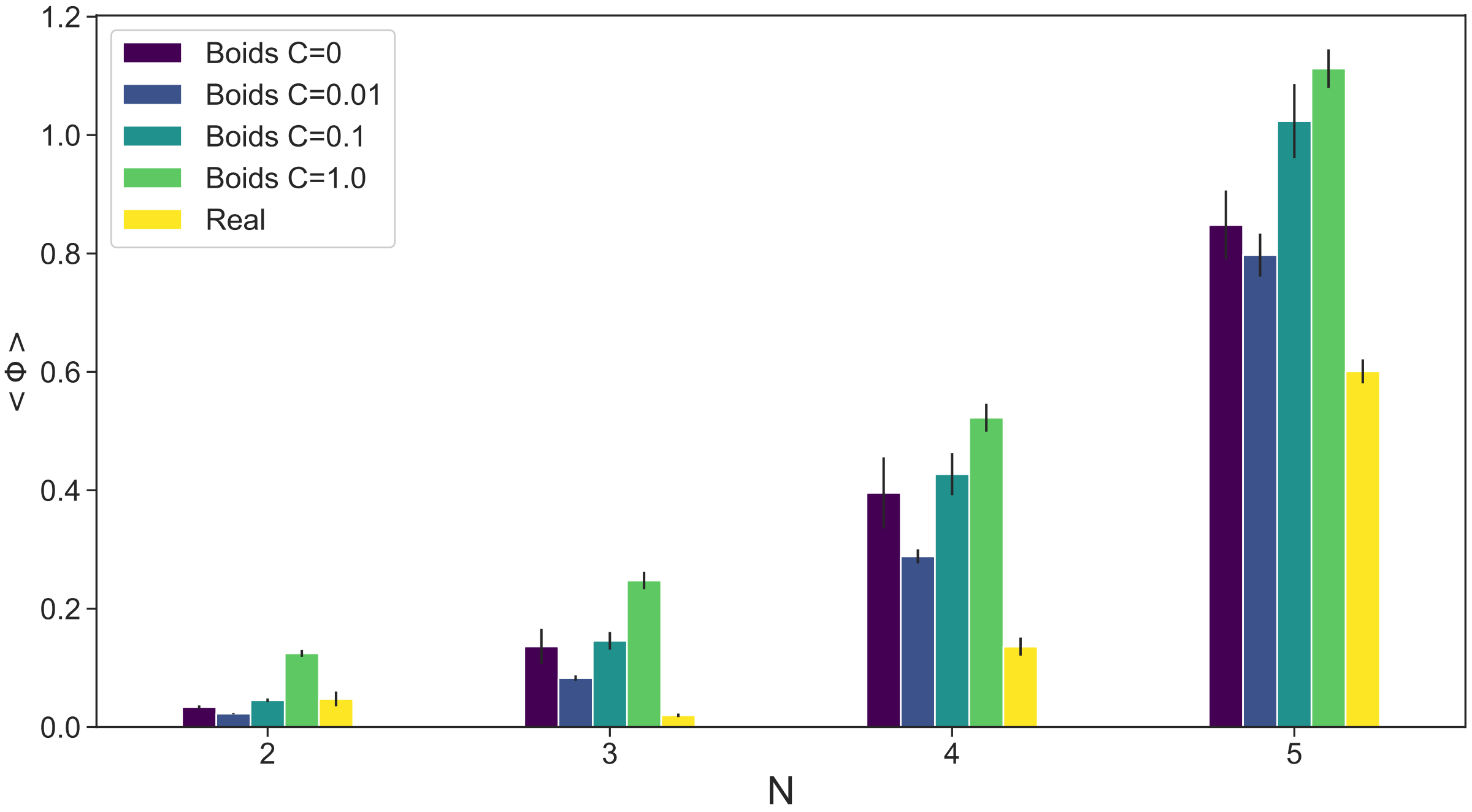

Supplement: S11 Fig — The values of Φ were averaged over the distance–visual field parameter space and again averaged for 10 simulations and all real data. Error bars indicate the standard deviations. (PDF) [file pone.0229573.s011.pdf]
