# Supplementary material for: Finding continuity and discontinuity in fish schools via integrated information theory
Source: PLoS One. 2020 Feb 27;15(2):e0229573. doi: 10.1371/journal.pone.0229573 (PMC7046263; doi:10.1371/journal.pone.0229573)
Supplement: S2 Fig — (PDF) [file pone.0229573.s002.pdf]

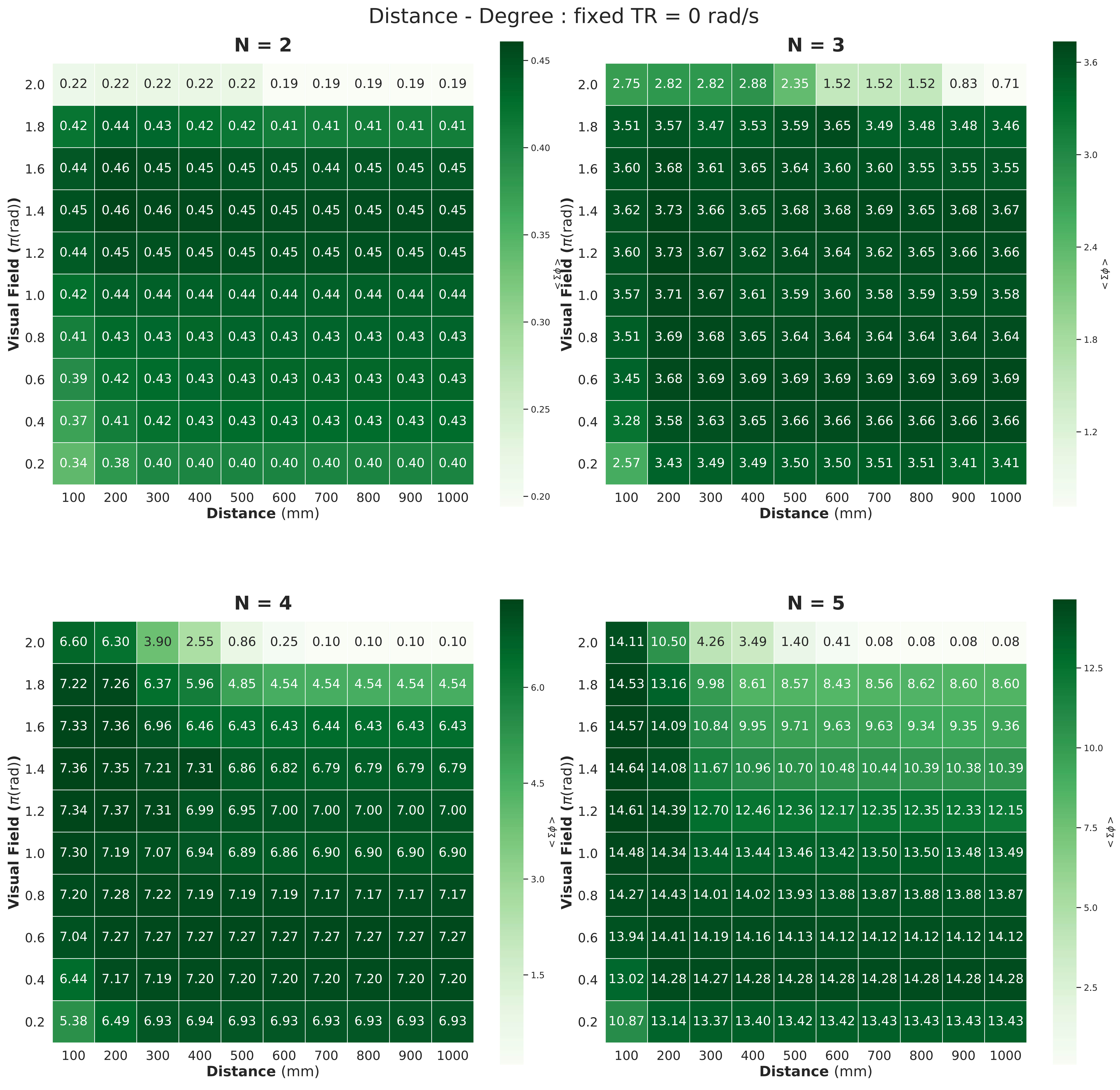
Distance - Degree : fixed TR = 0 rad/s
N = 2
Visual Field (π(rad))
Distance (mm)
<Σϕ>
2.0 0.22 0.22 0.22 0.22 0.22 0.19 0.19 0.19 0.19 0.19
1.8 0.42 0.44 0.43 0.42 0.42 0.41 0.41 0.41 0.41 0.41
1.6 0.44 0.46 0.45 0.45 0.45 0.45 0.44 0.45 0.45 0.45
1.4 0.45 0.46 0.46 0.45 0.45 0.45 0.45 0.45 0.45 0.45
1.2 0.44 0.45 0.45 0.45 0.45 0.45 0.45 0.45 0.45 0.45
1.0 0.42 0.44 0.44 0.44 0.44 0.44 0.44 0.44 0.44 0.44
0.8 0.41 0.43 0.43 0.43 0.43 0.43 0.43 0.43 0.43 0.43
0.6 0.39 0.42 0.43 0.43 0.43 0.43 0.43 0.43 0.43 0.43
0.4 0.37 0.41 0.42 0.43 0.43 0.43 0.43 0.43 0.43 0.43
0.2 0.34 0.38 0.40 0.40 0.40 0.40 0.40 0.40 0.40 0.40
100 200 300 400 500 600 700 800 900 1000
0.45 0.40 0.35 0.30 0.25 0.20
N = 3
Visual Field (π(rad))
Distance (mm)
<Σϕ>
2.0 2.75 2.82 2.82 2.88 2.35 1.52 1.52 1.52 0.83 0.71
1.8 3.51 3.57 3.47 3.53 3.59 3.65 3.49 3.48 3.48 3.46
1.6 3.60 3.68 3.61 3.65 3.64 3.60 3.60 3.55 3.55 3.55
1.4 3.62 3.73 3.66 3.65 3.68 3.68 3.69 3.65 3.68 3.67
1.2 3.60 3.73 3.67 3.62 3.64 3.64 3.62 3.65 3.66 3.66
1.0 3.57 3.71 3.67 3.61 3.59 3.60 3.58 3.59 3.59 3.58
0.8 3.51 3.69 3.68 3.65 3.64 3.64 3.64 3.64 3.64 3.64
0.6 3.45 3.68 3.69 3.69 3.69 3.69 3.69 3.69 3.69 3.69
0.4 3.28 3.58 3.63 3.65 3.66 3.66 3.66 3.66 3.66 3.66
0.2 2.57 3.43 3.49 3.49 3.50 3.50 3.51 3.51 3.41 3.41
100 200 300 400 500 600 700 800 900 1000
3.6 3.0 2.4 1.8 1.2
N = 4
Visual Field (π(rad))
Distance (mm)
<Σϕ>
2.0 6.60 6.30 3.90 2.55 0.86 0.25 0.10 0.10 0.10 0.10
1.8 7.22 7.26 6.37 5.96 4.85 4.54 4.54 4.54 4.54 4.54
1.6 7.33 7.36 6.96 6.46 6.43 6.43 6.44 6.43 6.43 6.43
1.4 7.36 7.35 7.21 7.31 6.86 6.82 6.79 6.79 6.79 6.79
1.2 7.34 7.37 7.31 6.99 6.95 7.00 7.00 7.00 7.00 7.00
1.0 7.30 7.19 7.07 6.94 6.89 6.86 6.90 6.90 6.90 6.90
0.8 7.20 7.28 7.22 7.19 7.19 7.19 7.17 7.17 7.17 7.17
0.6 7.04 7.27 7.27 7.27 7.27 7.27 7.27 7.27 7.27 7.27
0.4 6.44 7.17 7.19 7.20 7.20 7.20 7.20 7.20 7.20 7.20
0.2 5.38 6.49 6.93 6.94 6.93 6.93 6.93 6.93 6.93 6.93
100 200 300 400 500 600 700 800 900 1000
6.0 4.5 3.0 1.5
N = 5
Visual Field (π(rad))
Distance (mm)
<Σϕ>
2.0 14.11 10.50 4.26 3.49 1.40 0.41 0.08 0.08 0.08 0.08
1.8 14.53 13.16 9.98 8.61 8.57 8.43 8.56 8.62 8.60 8.60
1.6 14.57 14.09 10.84 9.95 9.71 9.63 9.63 9.34 9.35 9.36
1.4 14.64 14.08 11.67 10.96 10.70 10.48 10.44 10.39 10.38 10.39
1.2 14.61 14.39 12.70 12.46 12.36 12.17 12.35 12.35 12.33 12.15
1.0 14.48 14.34 13.44 13.44 13.46 13.42 13.50 13.50 13.48 13.49
0.8 14.27 14.43 14.01 14.02 13.93 13.88 13.87 13.88 13.88 13.87
0.6 13.94 14.41 14.19 14.16 14.13 14.12 14.12 14.12 14.12 14.12
0.4 13.02 14.28 14.27 14.28 14.28 14.28 14.28 14.28 14.28 14.28
0.2 10.87 13.14 13.37 13.40 13.42 13.42 13.43 13.43 13.43 13.43
100 200 300 400 500 600 700 800 900 1000
12.5 10.0 7.5 5.0 2.5

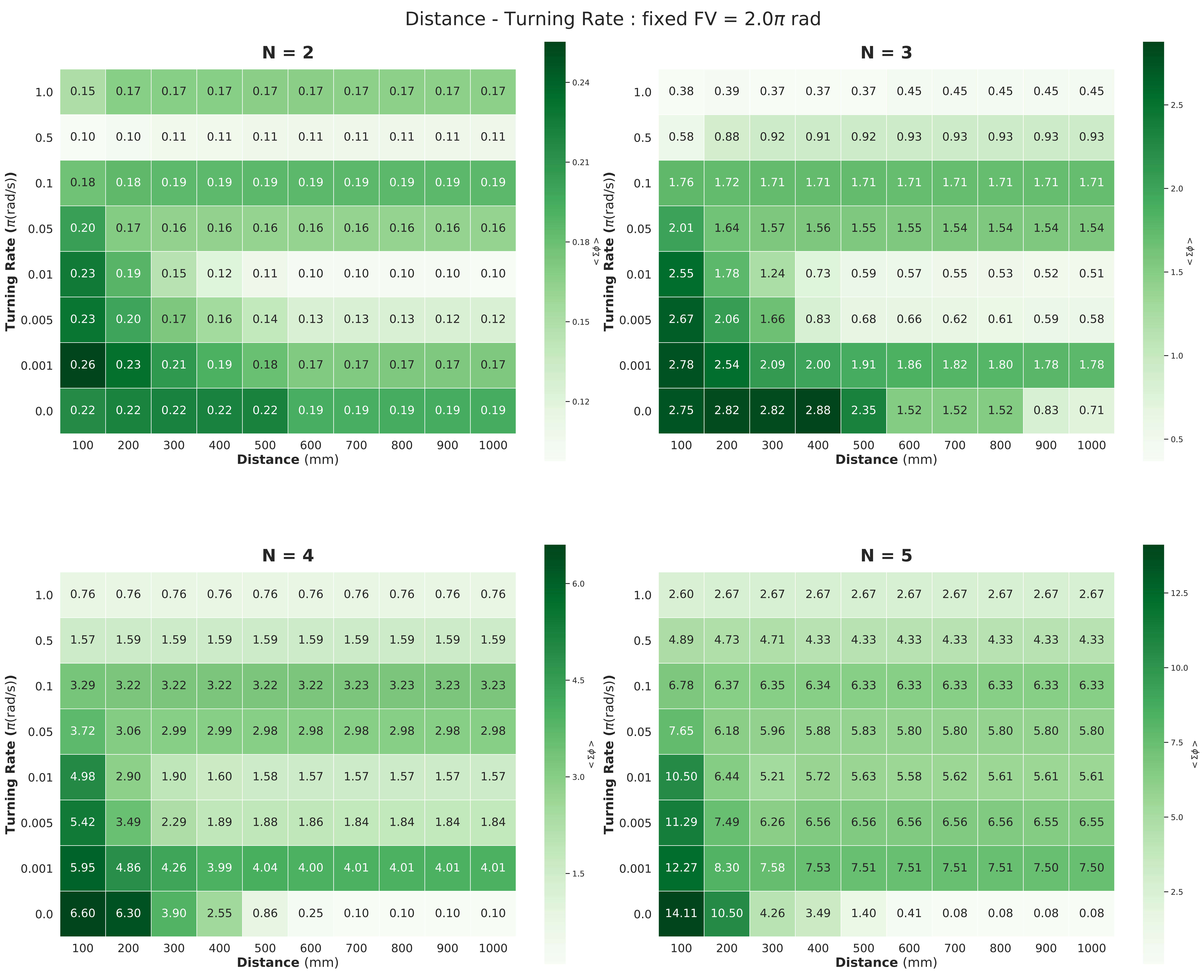

Distance - Turning Rate : fixed FV = 2.0π rad
N = 2
1.0 0.15 0.17 0.17 0.17 0.17 0.17 0.17 0.17 0.17 0.17
0.5 0.10 0.10 0.11 0.11 0.11 0.11 0.11 0.11 0.11 0.11
0.1 0.18 0.18 0.19 0.19 0.19 0.19 0.19 0.19 0.19 0.19
0.05 0.20 0.17 0.16 0.16 0.16 0.16 0.16 0.16 0.16 0.16
0.01 0.23 0.19 0.15 0.12 0.11 0.10 0.10 0.10 0.10 0.10
0.005 0.23 0.20 0.17 0.16 0.14 0.13 0.13 0.13 0.12 0.12
0.001 0.26 0.23 0.21 0.19 0.18 0.17 0.17 0.17 0.17 0.17
0.0 0.22 0.22 0.22 0.22 0.22 0.19 0.19 0.19 0.19 0.19
100 200 300 400 500 600 700 800 900 1000
Turning Rate (π(rad/s))
Distance (mm)
0.24 0.21 0.18 0.15 0.12
< Σφ >
N = 3
1.0 0.38 0.39 0.37 0.37 0.37 0.45 0.45 0.45 0.45 0.45
0.5 0.58 0.88 0.92 0.91 0.92 0.93 0.93 0.93 0.93 0.93
0.1 1.76 1.72 1.71 1.71 1.71 1.71 1.71 1.71 1.71 1.71
0.05 2.01 1.64 1.57 1.56 1.55 1.55 1.54 1.54 1.54 1.54
0.01 2.55 1.78 1.24 0.73 0.59 0.57 0.55 0.53 0.52 0.51
0.005 2.67 2.06 1.66 0.83 0.68 0.66 0.62 0.61 0.59 0.58
0.001 2.78 2.54 2.09 2.00 1.91 1.86 1.82 1.80 1.78 1.78
0.0 2.75 2.82 2.82 2.88 2.35 1.52 1.52 1.52 0.83 0.71
100 200 300 400 500 600 700 800 900 1000
Turning Rate (π(rad/s))
Distance (mm)
2.5 2.0 1.5 1.0 0.5
< Σφ >
N = 4
1.0 0.76 0.76 0.76 0.76 0.76 0.76 0.76 0.76 0.76 0.76
0.5 1.57 1.59 1.59 1.59 1.59 1.59 1.59 1.59 1.59 1.59
0.1 3.29 3.22 3.22 3.22 3.22 3.22 3.23 3.23 3.23 3.23
0.05 3.72 3.06 2.99 2.99 2.98 2.98 2.98 2.98 2.98 2.98
0.01 4.98 2.90 1.90 1.60 1.58 1.57 1.57 1.57 1.57 1.57
0.005 5.42 3.49 2.29 1.89 1.88 1.86 1.84 1.84 1.84 1.84
0.001 5.95 4.86 4.26 3.99 4.04 4.00 4.01 4.01 4.01 4.01
0.0 6.60 6.30 3.90 2.55 0.86 0.25 0.10 0.10 0.10 0.10
100 200 300 400 500 600 700 800 900 1000
Turning Rate (π(rad/s))
Distance (mm)
6.0 4.5 3.0 1.5
< Σφ >
N = 5
1.0 2.60 2.67 2.67 2.67 2.67 2.67 2.67 2.67 2.67 2.67
0.5 4.89 4.73 4.71 4.33 4.33 4.33 4.33 4.33 4.33 4.33
0.1 6.78 6.37 6.35 6.34 6.33 6.33 6.33 6.33 6.33 6.33
0.05 7.65 6.18 5.96 5.88 5.83 5.80 5.80 5.80 5.80 5.80
0.01 10.50 6.44 5.21 5.72 5.63 5.58 5.62 5.61 5.61 5.61
0.005 11.29 7.49 6.26 6.56 6.56 6.56 6.56 6.56 6.55 6.55
0.001 12.27 8.30 7.58 7.53 7.51 7.51 7.51 7.51 7.50 7.50
0.0 14.11 10.50 4.26 3.49 1.40 0.41 0.08 0.08 0.08 0.08
100 200 300 400 500 600 700 800 900 1000
Turning Rate (π(rad/s))
Distance (mm)
12.5 10.0 7.5 5.0 2.5
< Σφ >

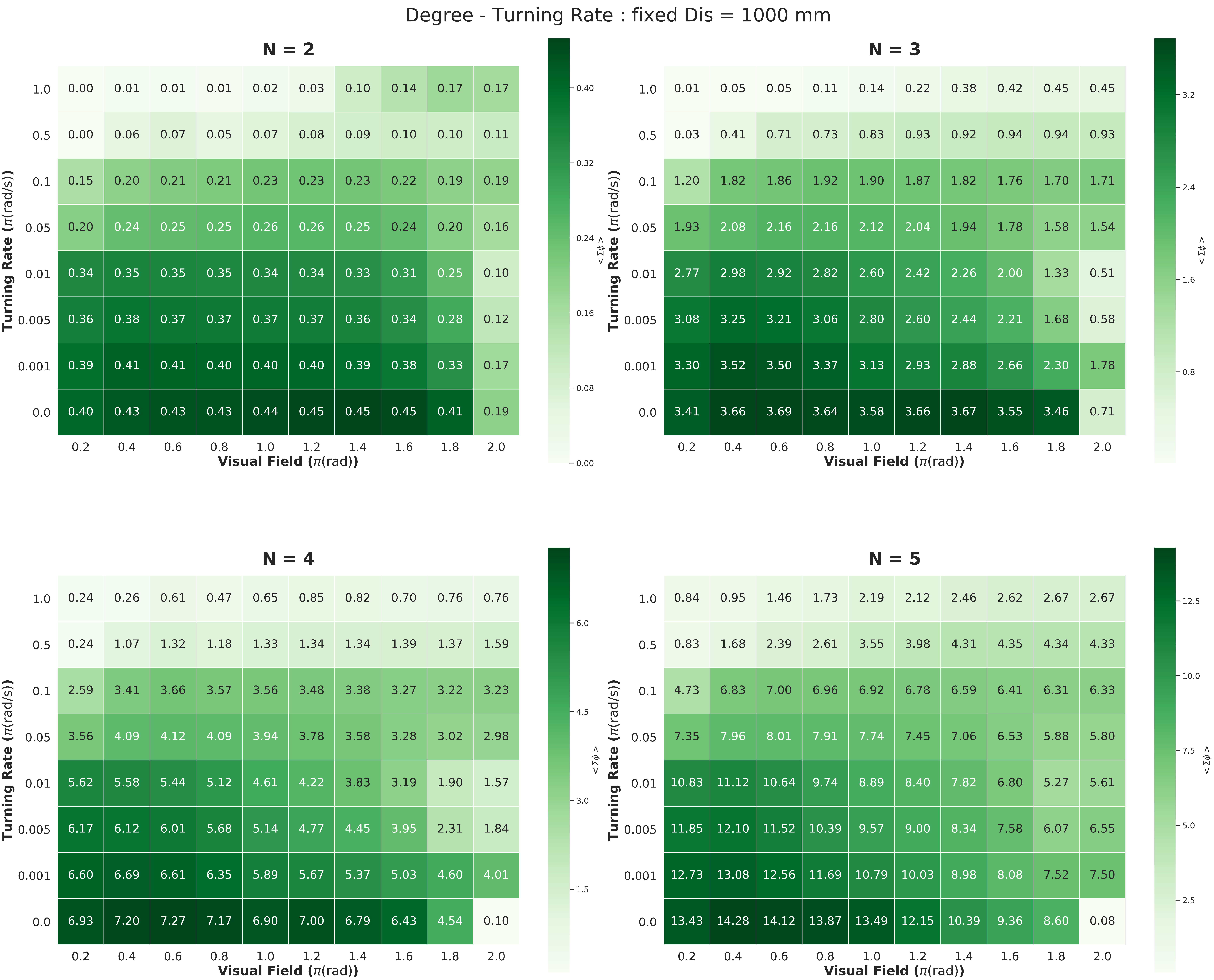
Degree - Turning Rate : fixed Dis = 1000 mm
N = 2
1.0 0.00 0.01 0.01 0.01 0.02 0.03 0.10 0.14 0.17 0.17
0.5 0.00 0.06 0.07 0.05 0.07 0.08 0.09 0.10 0.10 0.11
0.1 0.15 0.20 0.21 0.21 0.23 0.23 0.23 0.22 0.19 0.19
0.05 0.20 0.24 0.25 0.25 0.26 0.26 0.25 0.24 0.20 0.16
0.01 0.34 0.35 0.35 0.35 0.34 0.34 0.33 0.31 0.25 0.10
0.005 0.36 0.38 0.37 0.37 0.37 0.37 0.36 0.34 0.28 0.12
0.001 0.39 0.41 0.41 0.40 0.40 0.40 0.39 0.38 0.33 0.17
0.0 0.40 0.43 0.43 0.43 0.44 0.45 0.45 0.45 0.41 0.19
0.2 0.4 0.6 0.8 1.0 1.2 1.4 1.6 1.8 2.0
Turning Rate (π(rad/s))
Visual Field (π(rad))
0.00 0.08 0.16 0.24 0.32 0.40
< Σφ >
N = 3
1.0 0.01 0.05 0.05 0.11 0.14 0.22 0.38 0.42 0.45 0.45
0.5 0.03 0.41 0.71 0.73 0.83 0.93 0.92 0.94 0.94 0.93
0.1 1.20 1.82 1.86 1.92 1.90 1.87 1.82 1.76 1.70 1.71
0.05 1.93 2.08 2.16 2.16 2.12 2.04 1.94 1.78 1.58 1.54
0.01 2.77 2.98 2.92 2.82 2.60 2.42 2.26 2.00 1.33 0.51
0.005 3.08 3.25 3.21 3.06 2.80 2.60 2.44 2.21 1.68 0.58
0.001 3.30 3.52 3.50 3.37 3.13 2.93 2.88 2.66 2.30 1.78
0.0 3.41 3.66 3.69 3.64 3.58 3.66 3.67 3.55 3.46 0.71
0.2 0.4 0.6 0.8 1.0 1.2 1.4 1.6 1.8 2.0
Turning Rate (π(rad/s))
Visual Field (π(rad))
0.8 1.6 2.4 3.2
< Σφ >
N = 4
1.0 0.24 0.26 0.61 0.47 0.65 0.85 0.82 0.70 0.76 0.76
0.5 0.24 1.07 1.32 1.18 1.33 1.34 1.34 1.39 1.37 1.59
0.1 2.59 3.41 3.66 3.57 3.56 3.48 3.38 3.27 3.22 3.23
0.05 3.56 4.09 4.12 4.09 3.94 3.78 3.58 3.28 3.02 2.98
0.01 5.62 5.58 5.44 5.12 4.61 4.22 3.83 3.19 1.90 1.57
0.005 6.17 6.12 6.01 5.68 5.14 4.77 4.45 3.95 2.31 1.84
0.001 6.60 6.69 6.61 6.35 5.89 5.67 5.37 5.03 4.60 4.01
0.0 6.93 7.20 7.27 7.17 6.90 7.00 6.79 6.43 4.54 0.10
0.2 0.4 0.6 0.8 1.0 1.2 1.4 1.6 1.8 2.0
Turning Rate (π(rad/s))
Visual Field (π(rad))
1.5 3.0 4.5 6.0
< Σφ >
N = 5
1.0 0.84 0.95 1.46 1.73 2.19 2.12 2.46 2.62 2.67 2.67
0.5 0.83 1.68 2.39 2.61 3.55 3.98 4.31 4.35 4.34 4.33
0.1 4.73 6.83 7.00 6.96 6.92 6.78 6.59 6.41 6.31 6.33
0.05 7.35 7.96 8.01 7.91 7.74 7.45 7.06 6.53 5.88 5.80
0.01 10.83 11.12 10.64 9.74 8.89 8.40 7.82 6.80 5.27 5.61
0.005 11.85 12.10 11.52 10.39 9.57 9.00 8.34 7.58 6.07 6.55
0.001 12.73 13.08 12.56 11.69 10.79 10.03 8.98 8.08 7.52 7.50
0.0 13.43 14.28 14.12 13.87 13.49 12.15 10.39 9.36 8.60 0.08
0.2 0.4 0.6 0.8 1.0 1.2 1.4 1.6 1.8 2.0
Turning Rate (π(rad/s))
Visual Field (π(rad))
2.5 5.0 7.5 10.0 12.5
< Σφ >
