# Supplementary material for: Finding continuity and discontinuity in fish schools via integrated information theory
Source: PLoS One. 2020 Feb 27;15(2):e0229573. doi: 10.1371/journal.pone.0229573 (PMC7046263; doi:10.1371/journal.pone.0229573)
Supplement: S7 Fig — (PDF) [file pone.0229573.s007.pdf]

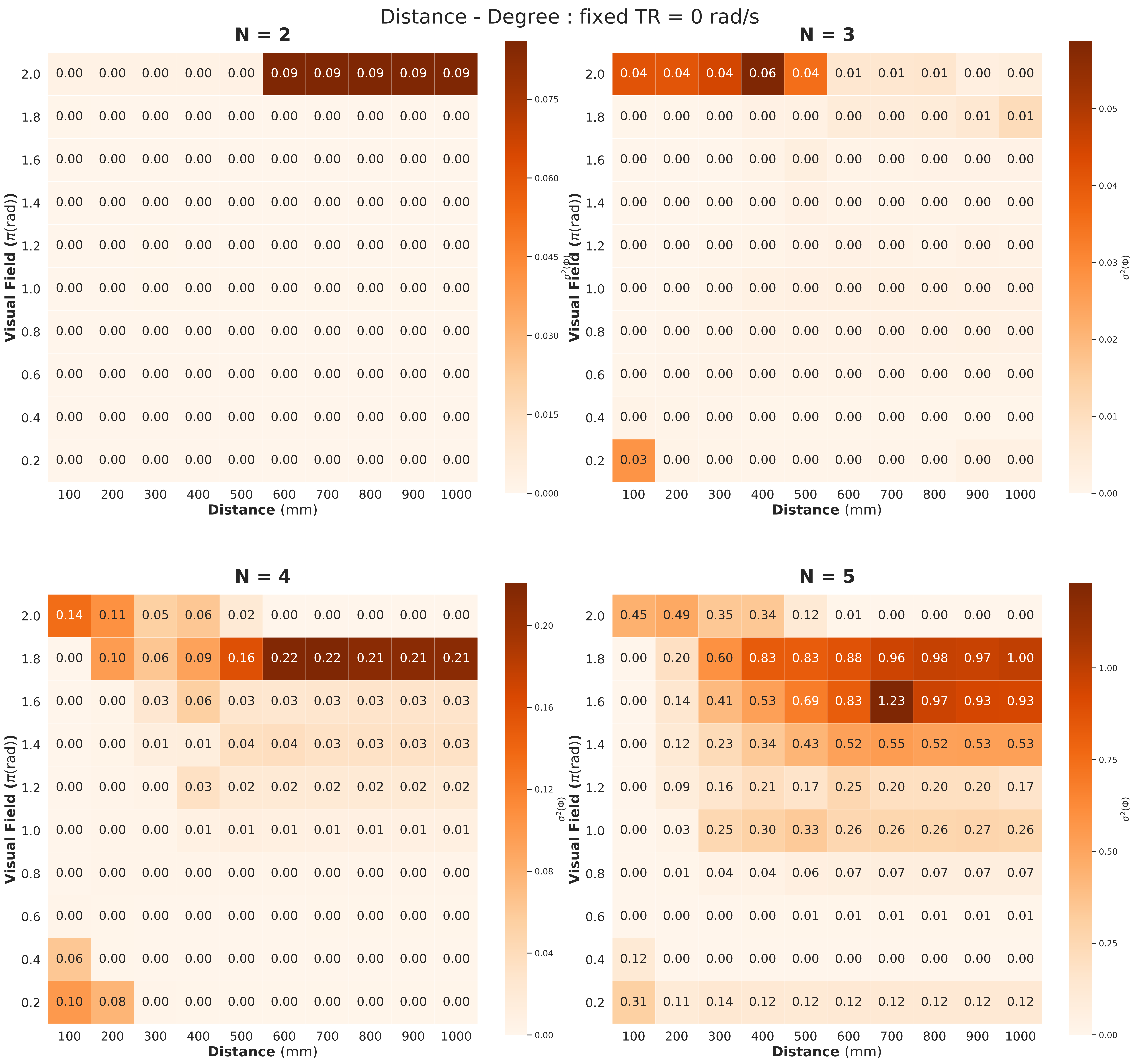

Distance - Degree : fixed TR = 0 rad/s
N = 2
2.0 0.00 0.00 0.00 0.00 0.00 0.09 0.09 0.09 0.09 0.09
1.8 0.00 0.00 0.00 0.00 0.00 0.00 0.00 0.00 0.00 0.00
1.6 0.00 0.00 0.00 0.00 0.00 0.00 0.00 0.00 0.00 0.00
1.4 0.00 0.00 0.00 0.00 0.00 0.00 0.00 0.00 0.00 0.00
1.2 0.00 0.00 0.00 0.00 0.00 0.00 0.00 0.00 0.00 0.00
1.0 0.00 0.00 0.00 0.00 0.00 0.00 0.00 0.00 0.00 0.00
0.8 0.00 0.00 0.00 0.00 0.00 0.00 0.00 0.00 0.00 0.00
0.6 0.00 0.00 0.00 0.00 0.00 0.00 0.00 0.00 0.00 0.00
0.4 0.00 0.00 0.00 0.00 0.00 0.00 0.00 0.00 0.00 0.00
0.2 0.00 0.00 0.00 0.00 0.00 0.00 0.00 0.00 0.00 0.00
100 200 300 400 500 600 700 800 900 1000
Distance (mm)
Visual Field (π(rad))
0.000 0.015 0.030 0.045 0.060 0.075
σ²(Φ)
N = 3
2.0 0.04 0.04 0.04 0.06 0.04 0.01 0.01 0.01 0.00 0.00
1.8 0.00 0.00 0.00 0.00 0.00 0.00 0.00 0.00 0.01 0.01
1.6 0.00 0.00 0.00 0.00 0.00 0.00 0.00 0.00 0.00 0.00
1.4 0.00 0.00 0.00 0.00 0.00 0.00 0.00 0.00 0.00 0.00
1.2 0.00 0.00 0.00 0.00 0.00 0.00 0.00 0.00 0.00 0.00
1.0 0.00 0.00 0.00 0.00 0.00 0.00 0.00 0.00 0.00 0.00
0.8 0.00 0.00 0.00 0.00 0.00 0.00 0.00 0.00 0.00 0.00
0.6 0.00 0.00 0.00 0.00 0.00 0.00 0.00 0.00 0.00 0.00
0.4 0.00 0.00 0.00 0.00 0.00 0.00 0.00 0.00 0.00 0.00
0.2 0.03 0.00 0.00 0.00 0.00 0.00 0.00 0.00 0.00 0.00
100 200 300 400 500 600 700 800 900 1000
Distance (mm)
Visual Field (π(rad))
0.00 0.01 0.02 0.03 0.04 0.05
σ²(Φ)
N = 4
2.0 0.14 0.11 0.05 0.06 0.02 0.00 0.00 0.00 0.00 0.00
1.8 0.00 0.10 0.06 0.09 0.16 0.22 0.22 0.21 0.21 0.21
1.6 0.00 0.00 0.03 0.06 0.03 0.03 0.03 0.03 0.03 0.03
1.4 0.00 0.00 0.01 0.01 0.04 0.04 0.03 0.03 0.03 0.03
1.2 0.00 0.00 0.00 0.03 0.02 0.02 0.02 0.02 0.02 0.02
1.0 0.00 0.00 0.00 0.01 0.01 0.01 0.01 0.01 0.01 0.01
0.8 0.00 0.00 0.00 0.00 0.00 0.00 0.00 0.00 0.00 0.00
0.6 0.00 0.00 0.00 0.00 0.00 0.00 0.00 0.00 0.00 0.00
0.4 0.06 0.00 0.00 0.00 0.00 0.00 0.00 0.00 0.00 0.00
0.2 0.10 0.08 0.00 0.00 0.00 0.00 0.00 0.00 0.00 0.00
100 200 300 400 500 600 700 800 900 1000
Distance (mm)
Visual Field (π(rad))
0.00 0.04 0.08 0.12 0.16 0.20
σ²(Φ)
N = 5
2.0 0.45 0.49 0.35 0.34 0.12 0.01 0.00 0.00 0.00 0.00
1.8 0.00 0.20 0.60 0.83 0.83 0.88 0.96 0.98 0.97 1.00
1.6 0.00 0.14 0.41 0.53 0.69 0.83 1.23 0.97 0.93 0.93
1.4 0.00 0.12 0.23 0.34 0.43 0.52 0.55 0.52 0.53 0.53
1.2 0.00 0.09 0.16 0.21 0.17 0.25 0.20 0.20 0.20 0.17
1.0 0.00 0.03 0.25 0.30 0.33 0.26 0.26 0.26 0.27 0.26
0.8 0.00 0.01 0.04 0.04 0.06 0.07 0.07 0.07 0.07 0.07
0.6 0.00 0.00 0.00 0.00 0.01 0.01 0.01 0.01 0.01 0.01
0.4 0.12 0.00 0.00 0.00 0.00 0.00 0.00 0.00 0.00 0.00
0.2 0.31 0.11 0.14 0.12 0.12 0.12 0.12 0.12 0.12 0.12
100 200 300 400 500 600 700 800 900 1000
Distance (mm)
Visual Field (π(rad))
0.00 0.25 0.50 0.75 1.00
σ²(Φ)

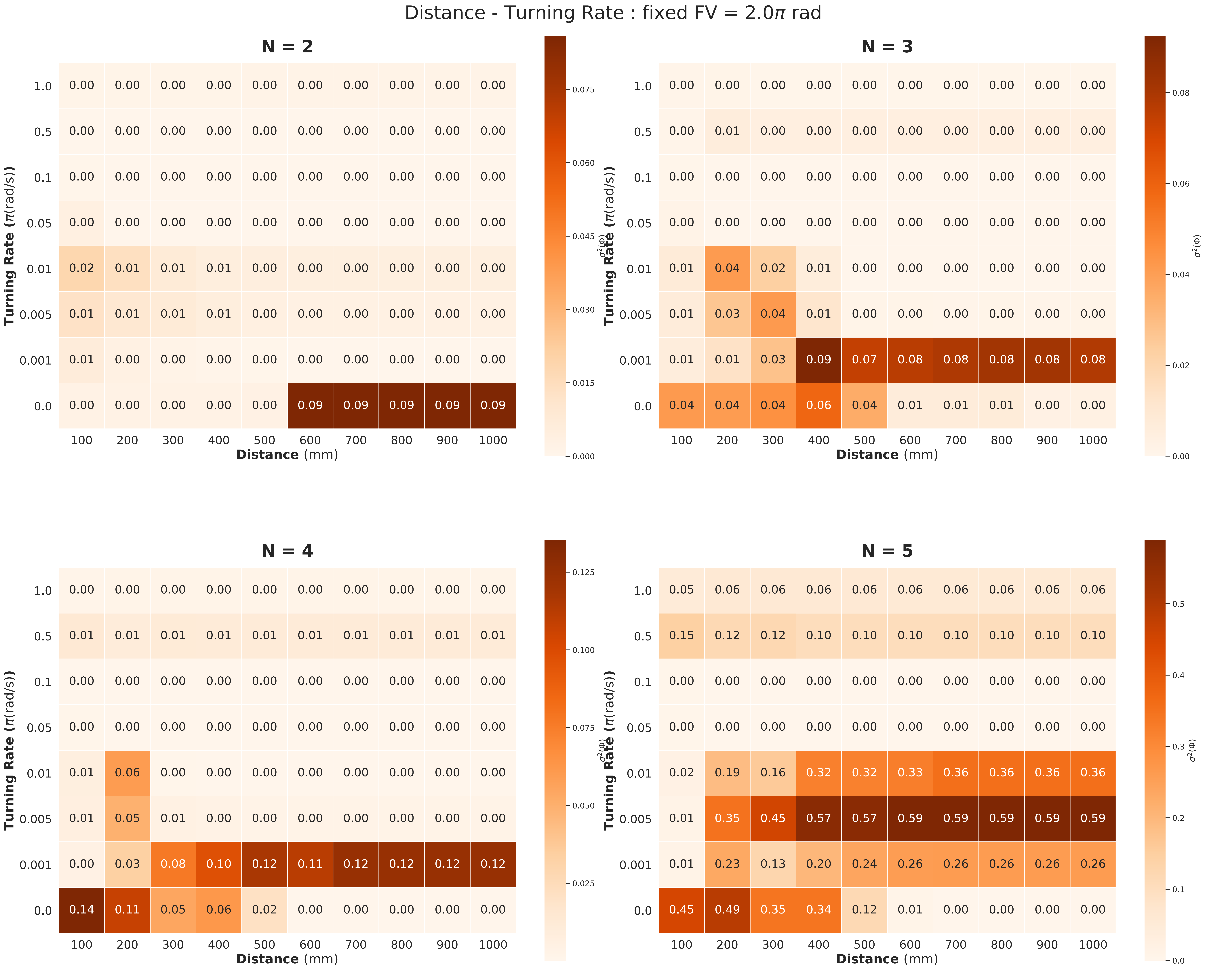
Distance - Turning Rate : fixed FV = 2.0π rad
N = 2
1.0 0.00 0.00 0.00 0.00 0.00 0.00 0.00 0.00 0.00 0.00
0.5 0.00 0.00 0.00 0.00 0.00 0.00 0.00 0.00 0.00 0.00
0.1 0.00 0.00 0.00 0.00 0.00 0.00 0.00 0.00 0.00 0.00
0.05 0.00 0.00 0.00 0.00 0.00 0.00 0.00 0.00 0.00 0.00
0.01 0.02 0.01 0.01 0.01 0.00 0.00 0.00 0.00 0.00 0.00
0.005 0.01 0.01 0.01 0.01 0.00 0.00 0.00 0.00 0.00 0.00
0.001 0.01 0.00 0.00 0.00 0.00 0.00 0.00 0.00 0.00 0.00
0.0 0.00 0.00 0.00 0.00 0.00 0.09 0.09 0.09 0.09 0.09
100 200 300 400 500 600 700 800 900 1000
Distance (mm)
Turning Rate (π(rad/s))
σ²(Φ)
0.000 0.015 0.030 0.045 0.060 0.075
N = 3
1.0 0.00 0.00 0.00 0.00 0.00 0.00 0.00 0.00 0.00 0.00
0.5 0.00 0.01 0.00 0.00 0.00 0.00 0.00 0.00 0.00 0.00
0.1 0.00 0.00 0.00 0.00 0.00 0.00 0.00 0.00 0.00 0.00
0.05 0.00 0.00 0.00 0.00 0.00 0.00 0.00 0.00 0.00 0.00
0.01 0.01 0.04 0.02 0.01 0.00 0.00 0.00 0.00 0.00 0.00
0.005 0.01 0.03 0.04 0.01 0.00 0.00 0.00 0.00 0.00 0.00
0.001 0.01 0.01 0.03 0.09 0.07 0.08 0.08 0.08 0.08 0.08
0.0 0.04 0.04 0.04 0.06 0.04 0.01 0.01 0.01 0.00 0.00
100 200 300 400 500 600 700 800 900 1000
Distance (mm)
Turning Rate (π(rad/s))
σ²(Φ)
0.00 0.02 0.04 0.06 0.08
N = 4
1.0 0.00 0.00 0.00 0.00 0.00 0.00 0.00 0.00 0.00 0.00
0.5 0.01 0.01 0.01 0.01 0.01 0.01 0.01 0.01 0.01 0.01
0.1 0.00 0.00 0.00 0.00 0.00 0.00 0.00 0.00 0.00 0.00
0.05 0.00 0.00 0.00 0.00 0.00 0.00 0.00 0.00 0.00 0.00
0.01 0.01 0.06 0.00 0.00 0.00 0.00 0.00 0.00 0.00 0.00
0.005 0.01 0.05 0.01 0.00 0.00 0.00 0.00 0.00 0.00 0.00
0.001 0.00 0.03 0.08 0.10 0.12 0.11 0.12 0.12 0.12 0.12
0.0 0.14 0.11 0.05 0.06 0.02 0.00 0.00 0.00 0.00 0.00
100 200 300 400 500 600 700 800 900 1000
Distance (mm)
Turning Rate (π(rad/s))
σ²(Φ)
0.025 0.050 0.075 0.100 0.125
N = 5
1.0 0.05 0.06 0.06 0.06 0.06 0.06 0.06 0.06 0.06 0.06
0.5 0.15 0.12 0.12 0.10 0.10 0.10 0.10 0.10 0.10 0.10
0.1 0.00 0.00 0.00 0.00 0.00 0.00 0.00 0.00 0.00 0.00
0.05 0.00 0.00 0.00 0.00 0.00 0.00 0.00 0.00 0.00 0.00
0.01 0.02 0.19 0.16 0.32 0.32 0.33 0.36 0.36 0.36 0.36
0.005 0.01 0.35 0.45 0.57 0.57 0.59 0.59 0.59 0.59 0.59
0.001 0.01 0.23 0.13 0.20 0.24 0.26 0.26 0.26 0.26 0.26
0.0 0.45 0.49 0.35 0.34 0.12 0.01 0.00 0.00 0.00 0.00
100 200 300 400 500 600 700 800 900 1000
Distance (mm)
Turning Rate (π(rad/s))
σ²(Φ)
0.0 0.1 0.2 0.3 0.4 0.5

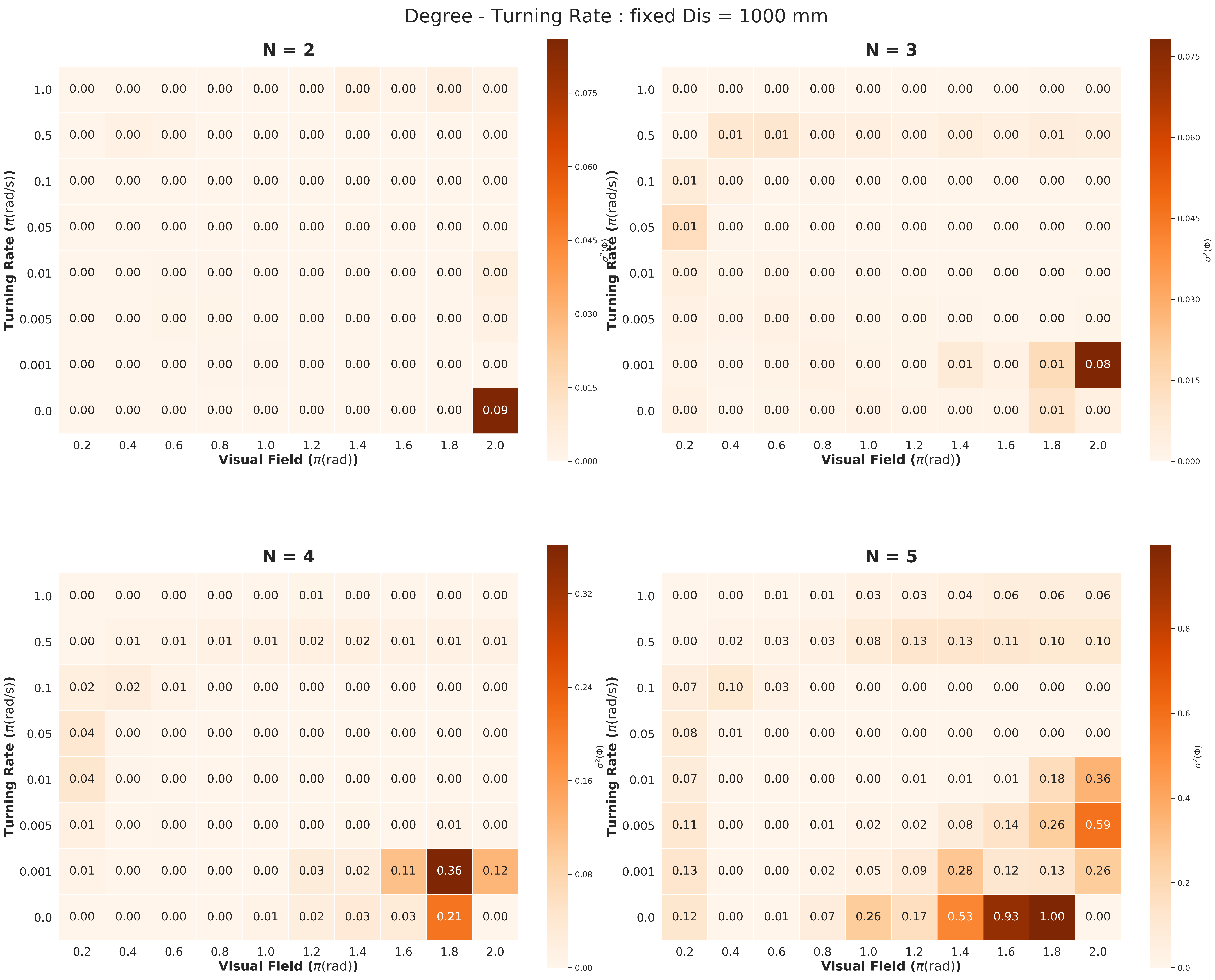
Degree - Turning Rate : fixed Dis = 1000 mm
N = 2
1.0 0.00 0.00 0.00 0.00 0.00 0.00 0.00 0.00 0.00 0.00
0.5 0.00 0.00 0.00 0.00 0.00 0.00 0.00 0.00 0.00 0.00
0.1 0.00 0.00 0.00 0.00 0.00 0.00 0.00 0.00 0.00 0.00
0.05 0.00 0.00 0.00 0.00 0.00 0.00 0.00 0.00 0.00 0.00
0.01 0.00 0.00 0.00 0.00 0.00 0.00 0.00 0.00 0.00 0.00
0.005 0.00 0.00 0.00 0.00 0.00 0.00 0.00 0.00 0.00 0.00
0.001 0.00 0.00 0.00 0.00 0.00 0.00 0.00 0.00 0.00 0.00
0.0 0.00 0.00 0.00 0.00 0.00 0.00 0.00 0.00 0.00 0.09
0.2 0.4 0.6 0.8 1.0 1.2 1.4 1.6 1.8 2.0
Turning Rate (π(rad/s))
Visual Field (π(rad))
0.000 0.015 0.030 0.045 0.060 0.075
σ²(Φ)
N = 3
1.0 0.00 0.00 0.00 0.00 0.00 0.00 0.00 0.00 0.00 0.00
0.5 0.00 0.01 0.01 0.00 0.00 0.00 0.00 0.00 0.01 0.00
0.1 0.01 0.00 0.00 0.00 0.00 0.00 0.00 0.00 0.00 0.00
0.05 0.01 0.00 0.00 0.00 0.00 0.00 0.00 0.00 0.00 0.00
0.01 0.00 0.00 0.00 0.00 0.00 0.00 0.00 0.00 0.00 0.00
0.005 0.00 0.00 0.00 0.00 0.00 0.00 0.00 0.00 0.00 0.00
0.001 0.00 0.00 0.00 0.00 0.00 0.00 0.01 0.00 0.01 0.08
0.0 0.00 0.00 0.00 0.00 0.00 0.00 0.00 0.00 0.01 0.00
0.2 0.4 0.6 0.8 1.0 1.2 1.4 1.6 1.8 2.0
Turning Rate (π(rad/s))
Visual Field (π(rad))
0.000 0.015 0.030 0.045 0.060 0.075
σ²(Φ)
N = 4
1.0 0.00 0.00 0.00 0.00 0.00 0.01 0.00 0.00 0.00 0.00
0.5 0.00 0.01 0.01 0.01 0.01 0.02 0.02 0.01 0.01 0.01
0.1 0.02 0.02 0.01 0.00 0.00 0.00 0.00 0.00 0.00 0.00
0.05 0.04 0.00 0.00 0.00 0.00 0.00 0.00 0.00 0.00 0.00
0.01 0.04 0.00 0.00 0.00 0.00 0.00 0.00 0.00 0.00 0.00
0.005 0.01 0.00 0.00 0.00 0.00 0.00 0.00 0.00 0.01 0.00
0.001 0.01 0.00 0.00 0.00 0.00 0.03 0.02 0.11 0.36 0.12
0.0 0.00 0.00 0.00 0.00 0.01 0.02 0.03 0.03 0.21 0.00
0.2 0.4 0.6 0.8 1.0 1.2 1.4 1.6 1.8 2.0
Turning Rate (π(rad/s))
Visual Field (π(rad))
0.00 0.08 0.16 0.24 0.32
σ²(Φ)
N = 5
1.0 0.00 0.00 0.01 0.01 0.03 0.03 0.04 0.06 0.06 0.06
0.5 0.00 0.02 0.03 0.03 0.08 0.13 0.13 0.11 0.10 0.10
0.1 0.07 0.10 0.03 0.00 0.00 0.00 0.00 0.00 0.00 0.00
0.05 0.08 0.01 0.00 0.00 0.00 0.00 0.00 0.00 0.00 0.00
0.01 0.07 0.00 0.00 0.00 0.00 0.01 0.01 0.01 0.18 0.36
0.005 0.11 0.00 0.00 0.01 0.02 0.02 0.08 0.14 0.26 0.59
0.001 0.13 0.00 0.00 0.02 0.05 0.09 0.28 0.12 0.13 0.26
0.0 0.12 0.00 0.01 0.07 0.26 0.17 0.53 0.93 1.00 0.00
0.2 0.4 0.6 0.8 1.0 1.2 1.4 1.6 1.8 2.0
Turning Rate (π(rad/s))
Visual Field (π(rad))
0.0 0.2 0.4 0.6 0.8
σ²(Φ)
